# Activation, Structure, Biosynthesis and Bioactivity of Glidobactin‐like Proteasome Inhibitors from *Photorhabdus laumondii*

**DOI:** 10.1002/cbic.202100014

**Published:** 2021-03-03

**Authors:** Lei Zhao, Camille Le Chapelain, Alexander O. Brachmann, Marcel Kaiser, Michael Groll, Helge B. Bode

**Affiliations:** ^1^ Molecular Biotechnology Department of Biosciences Goethe University Frankfurt 60438 Frankfurt am Main Germany; ^2^ Institute of Botany Jiangsu Province and Chinese Academy of Sciences 210014 Nanjing P. R. China; ^3^ Center for Integrated Protein Science Munich (CIPSM) Department of Chemistry Technical University of Munich 85748 Garching Germany; ^4^ Swiss Tropical and Public Health Institute 4002 Basel Switzerland; ^5^ Buchmann Institute for Molecular Life Sciences (BMLS) Goethe University Frankfurt 60438 Frankfurt am Main Germany; ^6^ Senckenberg Gesellschaft für Naturforschung 60325 Frankfurt am Main Germany; ^7^ Department of Natural Products in Organismic Interactions Max-Planck-Institute for Terrestrial Microbiology 35043 Marburg Germany

**Keywords:** biosynthesis, drug design, glidobactins, proteasome inhibitors, structure–activity relationships

## Abstract

The glidobactin‐like natural products (GLNPs) glidobactin A and cepafungin I have been reported to be potent proteasome inhibitors and are regarded as promising candidates for anticancer drug development. Their biosynthetic gene cluster (BGC) *plu1881–1877* is present in entomopathogenic *Photorhabdus laumondii* but silent under standard laboratory conditions. Here we show the largest subset of GLNPs, which are produced and identified after activation of the silent BGC in the native host and following heterologous expression of the BGC in *Escherichia coli*. Their chemical diversity results from a relaxed substrate specificity and flexible product release in the assembly line of GLNPs. Crystal structure analysis of the yeast proteasome in complex with new GLNPs suggests that the degree of unsaturation and the length of the aliphatic tail are critical for their bioactivity. The results in this study provide the basis to engineer the BGC for the generation of new GLNPs and to optimize these natural products resulting in potential drugs for cancer therapy.

## Graphical abstract

**The silent treatment**: Glidobactin‐like proteasome inhibitors are regarded as promising candidates for anticancer drug development. We report the largest subset of glidobactin‐like proteasome inhibitors, which are produced and identified after the silent biosynthetic gene cluster *plu1881–1877* is activated in the native host *P. laumondii*.

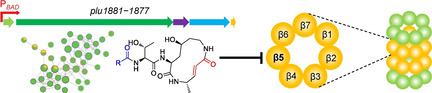

## Introduction

The ubiquitin‐proteasome system (UPS) is the main nonlysosomal protein degradation system responsible for the degradation of damaged, misfolded and excess proteins in all eukaryotic cells.[^1^, ^2^] It plays a crucial role in the dynamic regulation of protein turnover, which is essential for cell cycle, apoptosis, regulation of gene expression and other cellular functions.[^3^, ^4^] The eukaryotic 20S proteasome core particle (CP), a barrel‐shaped multicatalytic protease, represents the catalytic core of the UPS.[^1^, ^5^] The CP is composed of two identical outer α‐rings and two identical inner β‐rings with total 28 subunits that are arranged in α_1‐7_β_1‐7_β_1‐7_α_1‐7_ form.[^6^, ^7^] Three catalytic subunits β1, β2 and β5 present in each of the inner rings confer distinct caspase‐like (CL), trypsin‐like (TL) and chymotrypsin‐like (ChTL) proteolytic activities, respectively, with the active site threonine at their N‐termini.[^7^, ^8^, ^9^] Given the vital role in many cellular processes, the inhibition of the CP constitutes a promising target for the treatment of diverse diseases.^[10]^ Cancer cells in particular are sensitive to the inhibition, because they generally have higher levels of proteasome activity than normal cells, presumably due to their increased metabolism and higher levels of oxidative stress, cytokines, and growth factors.^[2]^ Thus, CP inhibitors can be an important class of drugs for cancer therapy and have received considerable attention in the past few decades.[^11^, ^12^] Currently, three inhibitors, bortezomib, carfilzomib and ixazomib, have been approved by the FDA for treating multiple myeloma.[^10^, ^11^] The primary mode of action of these drugs is the inhibition of the N‐terminal threonine of the ChTL β5 catalytic subunit.^[13]^ Despite the therapeutic advances, new proteasome inhibitors still need to be developed considering the drug resistance, severe side effects and the treatment of other tumors.[^14^, ^15^]

**Figure 1:**
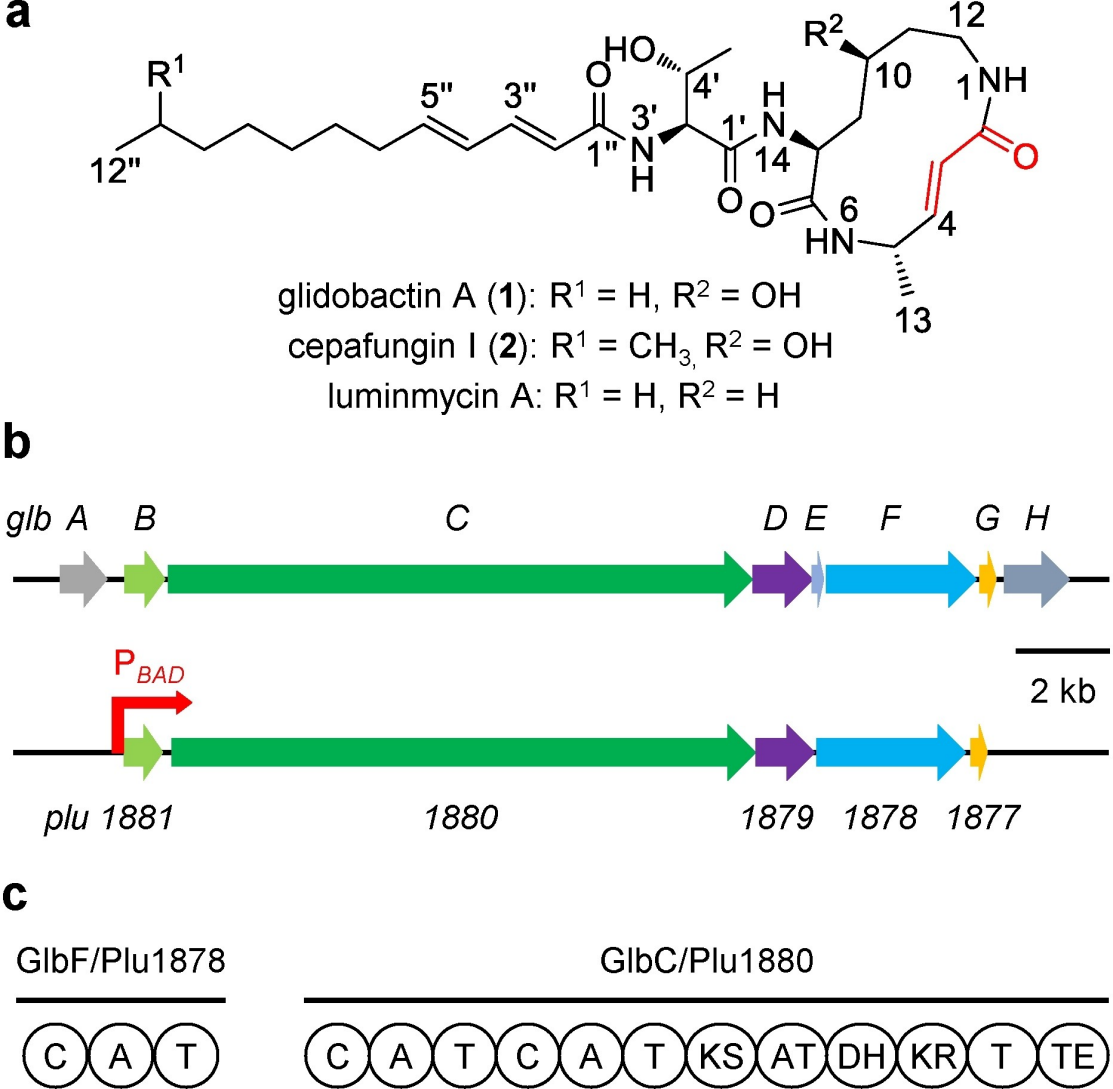
Select GLNP structures, BGC, and domain organization. a) Structures of glidobactin A and related natural products. The functional α,β‐unsaturated carbonyl group is marked red. b) BGC of glidobactins from *Burkholderia* K481‐B101 (*glbA−H*) and *P. laumondii* (*plu1881–1877*). Homologous genes are shown in identical colors. The putative functions of *glbA−H* in encoding proteins are as follows: GlbA: regulator, GlbB: lysine 4‐hydroxylase, GlbC: hybrid NRPS‐PKS, GlbD: transporter, GlbE: MbtH‐like protein, GlbF: NRPS, GlbG and GlbH: unknown. The position where the natural promoter in *plu1881–1877* is exchanged with the arabinose‐inducible promoter P_*BAD*_ is shown by a red arrow. c) Domain organization of the NRPS and hybrid NRPS‐PKS encoded by code biosynthetic genes *glbF/plu1878* and *glbC/plu1880*, respectively. Domains: C: condensation, A: adenylation, T: thiolation, KS: ketosynthase, AT: acyltransferase, DH: dehydratase, KR: ketoreductase, TE: thioesterase.

Glidobactin‐like natural products (GLNPs) such as glidobactins, cepafungins and luminmycins consist of similar structural scaffolds (Figure 1a).[^16^, ^17^] Luminmycins are the 10‐deoxy derivatives of glidobactins,^[18]^ while cepafungins only differ in the aliphatic tails.^[19]^ GLNPs characterize a common 12‐membered macrolactam ring with an α,β‐unsaturated carbonyl group. Bioactivity testing revealed their strong cytotoxicity against various human cancer cells.[^20^, ^21^] The mechanism behind the activity is the potent inhibition of proteasome.^[22]^ Crystal structure analysis of glidobactin A (**1**) and cepafungin I (**2**) in complex with yeast CP suggests that the inhibition occurs primarily by covalent and irreversible binding of the α,β‐unsaturated carbonyl moiety in the 12‐membered ring system of the inhibitors to the hydroxyl group of the active site threonine residue in the ChTL β5 subunit via ether bond formation from Michael‐type 1,4‐addition reaction.[^22^, ^23^] In addition, compounds **1** and **2** were also shown to inhibit the TL β2 subunit, whereas CL β1 subunit was not affected; in comparison with ChTL β5 subunit, the TL β2 subunit was less sensitive.^[23]^ With a reported IC_50_ value of 4 nM for the inhibition of yeast CP ChTL activity, compound **2** is the strongest natural proteasome inhibitor described to date, making it a promising candidate for further drug development.^[23]^

GLNPs are the products of mixed non‐ribosomal peptide synthetase (NRPS)/polyketide synthetase (PKS). Their biosynthetic gene cluster (BGC) was first identified from the soil bacterium *Burkholderia* K481‐B101.^[24]^ The BGC is composed of eight genes, named *glbA‐H* (Figure 1b), in which *glbF* and *glbC* encode a NRPS and a hybrid NRPS‐PKS, respectively, for the biosynthesis of the tripeptide part in GLNPs (Figure 1c).^[24]^ Bioinformatic analysis showed that the homologous BGC, consisting of five genes *plu1881–1877*, is also present in entomopathogenic *Photorhabdus laumondii* but lacking *glbA*, *glbE* and *glbH* homologues (Figure 1b).^[25]^ Thereby, *P. laumondii* was hypothesized to be able to produce a glidobactin‐type proteasome inhibitor. However, the BGC is silent or expressed at very low level even though *P. laumondii* was grown in various media and conditions in the laboratory.^[25]^ One explanation might be that the expression of the BGC is strictly regulated and solely induced by the specific environmental condition, in view of the unique niche of *P. laumondii* in the nematode‐symbiotic and insect‐pathogenic relationships.[^21^, ^26^] Herein, we report the activation, structure, biosynthesis and bioactivity of GLNP proteasome inhibitors from *P. laumondii*.

## Results and Discussion

In previous study, a heterologous expression of the *plu1881–1877* in *Pseudomonas putida*, which can bypass endogenous regulatory control, was found capable of producing **1**.^[25]^ Also, our effort of cloning the complete BGC into *Escherichia coli* resulted in successful production of **1** and its derivatives (Figure S1 in the Supporting Information). To investigate the functions of three small genes in *P. laumondii* for GLNP biosynthesis, heterologous *E. coli* strains with missing *plu1881*, *plu1879* and *plu1877* were constructed, and their products were identified by HPLC‐MS/MS analysis. The *plu1881* homologue *glbB* was recently identified to catalyze the 4‐hydroxylation reaction of l‐lysine.^[27]^ Expression of *plu1880–1877* without *plu1881* only generated 10‐deoxyglidobactins (Figure S2), verifying that Plu1881 has the same function as GlbB. The lack of the transporter Plu1879 did not show significant influence on GLNP production (Figure S3), suggesting that *plu1879* is not essential for GLNP biosynthesis in *E. coli*. Plu1877 belongs to NTF2‐like superfamily, including SnoaL polyketide cyclase, scytalone dehydratase and δ5‐3‐ketosteroid isomerase.^[28]^ Expressing *plu1881–1878* without *plu1877* mainly produced minimal amount of GLNPs with their aliphatic tails partly or completely reduced (Figure S4). Thereby *plu1877* might be involved in the synthesis of the unsaturated fatty acid moiety and it seems to play an important role in the biosynthesis of GLNPs.

**Figure 2:**
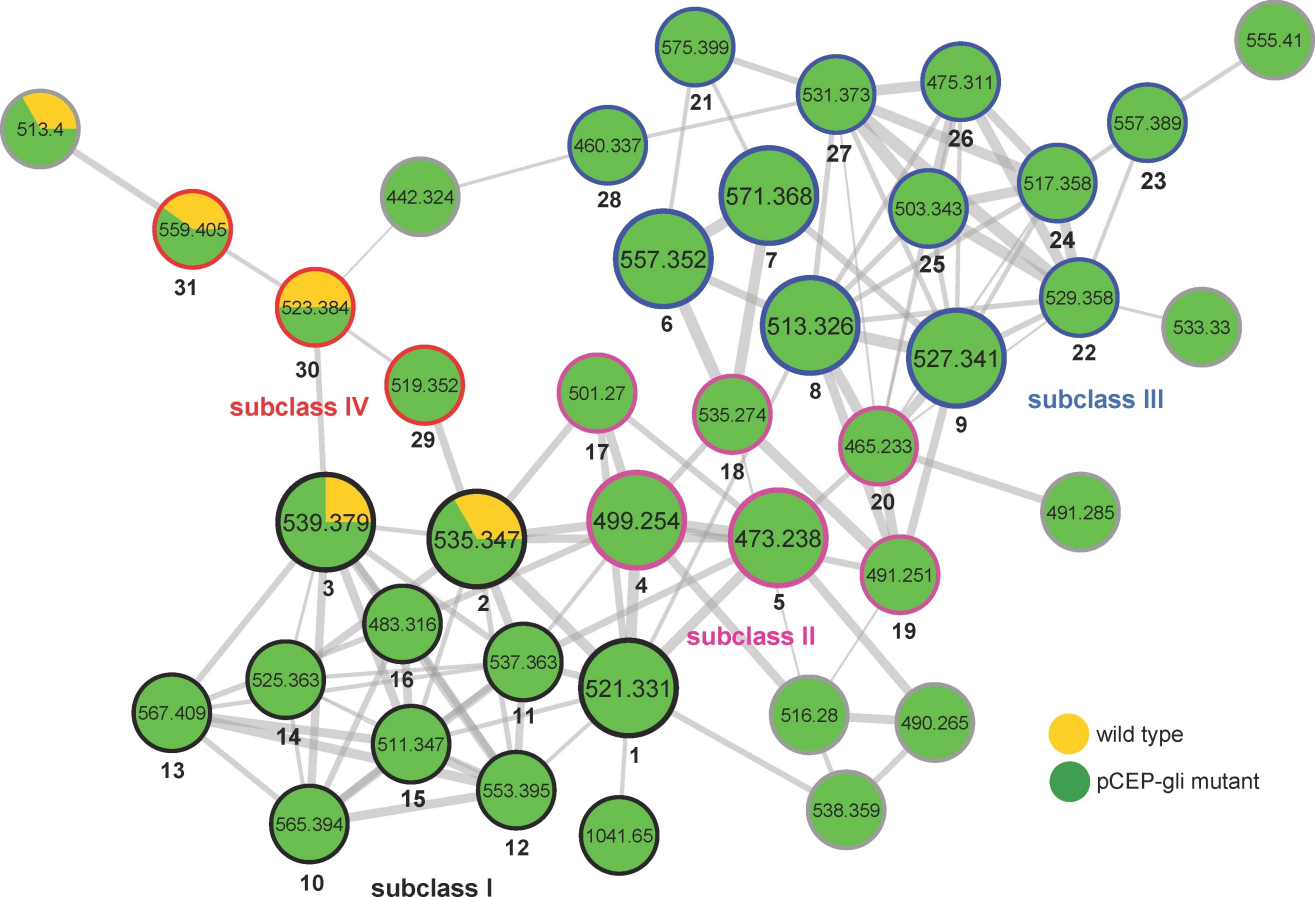
GLNP subnetwork of molecular networking for MeOH extracts of *P. laumondii* wild type and pCEP_gli mutant. The nodes in large circles represent the isolated derivatives (**1**–**9**). The edges of nodes in colors represent subclass I (black), II (pink), III (blue) and IV (red) of GLNPs. Detailed annotations for the 31 identified nodes (**1**–**31**) are presented in Table 1. The overall network is presented in Figure S5.

Although heterologous expression is one of the most frequently used strategies for the activation of silent BGCs, it is worth mentioning that the biosynthesis of correct products might be impossible if they are dependent on essential building blocks that cannot be synthesized by the heterologous host.[^29^, ^30^, ^31^] Therefore, in this study, a promoter exchange approach was also employed to activate the silent BGC *plu1881–1877* in the native host *P. laumondii* through exchanging the natural promoter against the well‐known arabinose‐inducible promoter P_*BAD*_. Assisted by molecular networking,^[32]^ the chemical diversity of GLNPs was revealed in the promoter exchange mutant *P. laumondii* pCEP_gli. As depicted in the molecular network (Figure 2), GLNPs are clustered into a large molecular family from the MeOH extracts. These nodes clearly represent far more GLNPs present in *P. laumondii* pCEP_gli mutant than in wild‐type strain when the strains were separately cultivated in a lysogeny broth (LB) medium under standard laboratory conditions.

In order to annotate these nodes, five major derivatives (**1**–**5**), along with four minor acyclic derivatives (**6**–**9**; Table 1), were isolated from the MeOH extract of *P. laumondii* pCEP_gli mutant by using Sephadex LH‐20 chromatography, followed by semipreparative HPLC. Their molecular formulas were determined by HR‐MS data (Table S1) and structures were elucidated by 1D and 2D NMR experiments (Table S2–11). Analysis of MS/MS fragmentation patterns of **1**–**9** further confirmed their structures (Figure S6). From known MS/MS fragmentations of derivatives **1**–**9**, the structures of the other minor derivatives can be deduced by detailed analysis of their MS/MS fragmentation patterns in combination with the HR‐MS data. In order to differentiate the N‐terminal branched‐chain fatty acids of GLNPs from the ones with straight‐chain fatty acids, a *P. laumondii ▵bkdABC* pCEP_gli mutant was constructed. Because of a missing branched‐chain ketoacid dehydrogenase (Bkd) complex, the *▵bkdABC* mutant is incapable of producing iso‐fatty acids.^[33]^ Hence *▵bkdABC* pCEP_gli mutant only accumulated straight‐chain fatty acid moiety containing derivatives (Figure S7). As GLNPs share high structural similarities and common biosynthetic origins, the absolute configurations of three amino acid residues in these metabolites were deduced to be the same as the previously reported analogues.[^18^, ^34^] However, low amounts of **6** and **7** prevent the assignment of the configurations at C4 by chemical degradation and derivatization. Based on the above efforts, in total 31 GLNPs (**1**−**31**; Table 1) were identified from the MeOH extract of *P. laumondii* pCEP_gli mutant. Interestingly, compound **4** with cinnamalacetic acid as the aliphatic tail instead of typical medium‐ or long‐chain fatty acids for most GLNPs was not observed in heterologous *E. coli*, but it is the main derivative produced by *P. laumondii* pCEP_gli mutant (Figure S10, Table S12). The cinnamic acid and cinnamalacetic acid derivatives were also not detected in the wild‐type strain. As the GLNP BGC is expressed at very low level in wild‐type strain, one speculation is that the wild‐type strain did not product this class of derivatives or their amounts are lower than the detection limit of HPLC‐MS.

**Table 1:** Compound list of **1**–**31**.

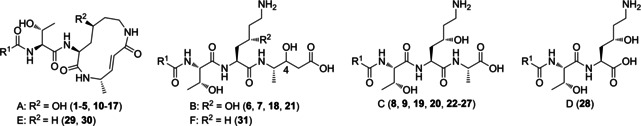									
Sub‐class	GLNP	R^1^	Core structure	Relative amount^b^	Sub‐class	GLNP	R^1^	Core structure	Relative amount^b^
I	**1**	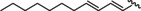	A	100 %	III	**6**	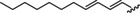	B	11 %
	**2**	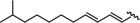	A	111 %		**7**	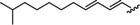	B	7 %
	**3**	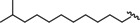	A	103 %		**8**	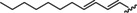	C	8 %
	**10**	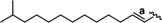	A	10 %		**9**	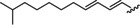	C	8 %
	**11**	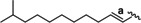	A	10 %		**21**	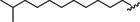	B	18 %
	**12**	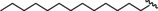	A	4 %		**22**	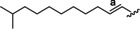	C	3 %
	**13**	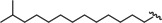	A	7 %		**23**	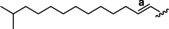	C	10 %
	**14**	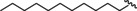	A	6 %		**24**	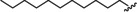	C	2 %
	**15**	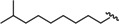	A	14 %		**25**	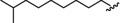	C	14 %
	**16**	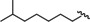	A	4 %		**26**	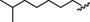	C	6 %
II	**4**	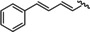	A	277 %		**27**	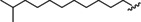	C	92 %
	**5**	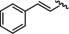	A	111 %		**28**	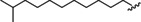	D	9 %
	**17**	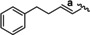	A	4 %	IV	**29**	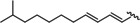	E	4 %
	**18**	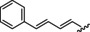	B	27 %		**30**	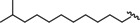	E	9 %
	**19**	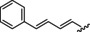	C	22 %		**31**	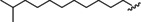	F	8 %
	**20**	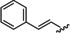	C	47 %

[a] The position of the double bond is not determined. [b] Production of **1** was normalized as 100 %, all the rest GLNPs were calculated based on **1**.

These identified GLNPs can be roughly divided into four subclasses (Figure 2, Table 1) according to their structural characteristics and biosynthetic logics. Subclass I represents the final products of *plu1881–1877* with complete 12‐membered ring system. They are assembled by three NRPS modules and one PKS module and eventually cyclized and released by thioesterase (TE) domain (Figure S8), in the light of the known biosynthesis of **1** catalyzed by GlbF and GlbC.[^24^, ^35^] Subclass II is a collection of novel cinnamic acid and cinnamalacetic acid containing GLNPs exclusively found in the promoter exchange mutant. To test whether cinnamic acid and cinnamalacetic acid moieties in these derivatives share the same biosynthetic pathways with isopropylstilbene (IPS) (Figure S9),[^33^, ^36^] three *P. laumondii* pCEP_gli strains were constructed by using three IPS‐negative mutants (*▵stlA*, *▵stlB*, and *▵stlCDE*). As expected, phenylalanine ammonium lyase (StlA)‐ and coenzyme A (CoA) ligase (StlB)‐deficient mutants did not produce this subclass of GLNPs (Figure S10), indicating that both cinnamic acid and cinnamalacetic acid are derived from phenylalanine and their activation is mediated by the CoA ligase. Unexpectedly, *▵stlCDE* pCEP_gli mutant still generated this family of derivatives but not IPS (Figure S10), suggesting an alternative biosynthetic pathway for the production of cinnamalacetic acid being incorporated as starting unit for GLNPs. Subclass III represents the open‐ring derivatives of subclass I. Their possible biosynthetic pathways were proposed according to the known luminmycin biosynthesis.^[18]^ These derivatives might be intermediates hydrolyzed spontaneously or catalyzed by the TE domain from different thiolation (T) domains using water as the nucleophile. For example, compound **28** might be directly hydrolyzed from the first T domain of Plu1880, leading to the absence of the 4‐amino‐2‐pentenoic acid moiety (Figure S11a). Compound **8** represents the hydrolysis product from the second T domain in Plu1880 without the involvement of the last PKS module, thus missing one PKS extender unit (Figure S11b). Compound **6** is the hydrolysis product from β‐hydroxyacyl‐S−T lacking dehydration at C4 by skipping dehydratase (DH) domain of the PKS module (Figure S11c). The identification of these intermediates supports the previously hypothetical biosynthetic pathway for **1**.[^24^, ^35^] The reason might be that the overproduction of GLNPs results in stalling of the enzyme‐bound intermediates that can then be hydrolyzed. Subclass IV consists of three minor deoxy derivatives at C10 and can be classified as luminmycins or deoxyglidobactins. They might derive from l‐lysine instead of (*S*)‐4‐hydroxy l‐lysine incorporated in the assembly line of GLNPs (Figure S8).

**Figure 3:**
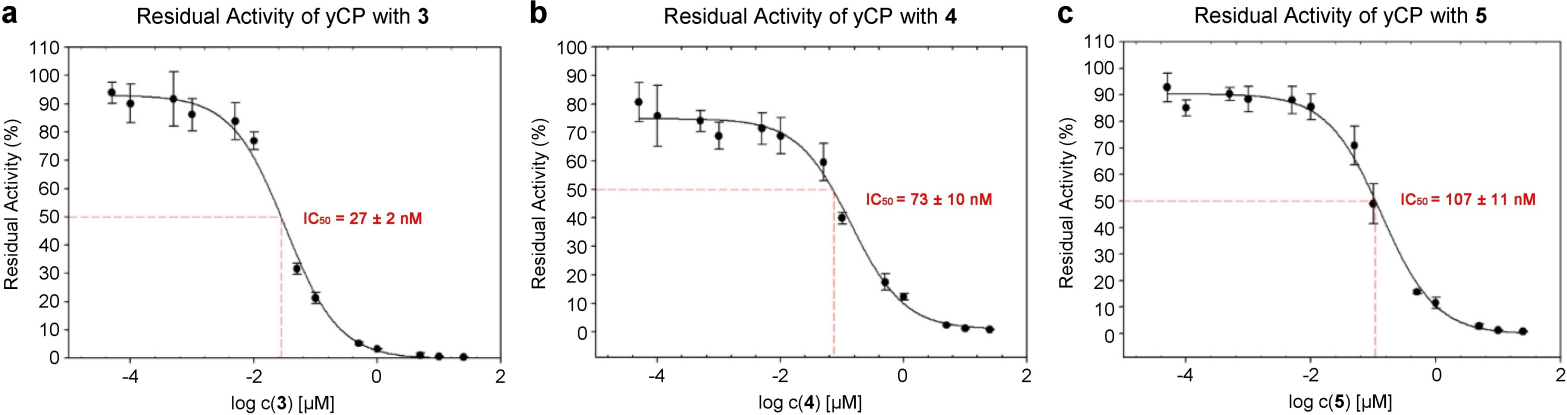
Dose–response curves for the residual ChTL activity of yCP after treatment with the inhibitors **3**–**5**. Residual activity as a function of the concentration of a) **3**, b) **4**, and c) **5**. Three replicates are shown as mean±SD (standard deviation). IC_50_ values were deduced from the curve.

In previous work, the open‐ring derivative luminmycin B did not show cytotoxic and antifungal activity.^[18]^ This is in agreement with the mechanism of proteasome inhibition relying on the functional reactive α,β‐unsaturated carbonyl moiety in the 12‐membered ring system.^[22]^ Moreover, terminal methyl branching in **2** exhibited a five times lower IC_50_ value (4 nM) in ChTL inhibitory activity compared to **1** (19 nM),[^23^, ^37^] suggesting that the aliphatic tail is critical for the proteasome inhibitory activity. Thus, the main compounds **3**–**5**, which possess the complete 12‐membered ring structures but contain different aliphatic tails, were investigated for their inhibitory potential against the ChTL activity of yeast CP. As expected, compounds **3**–**5** showed strong inhibitory activity with IC_50_ values of 27, 73 and 107 nM, respectively (Figure 3), which are weaker than that of **1** (19 nM) and **2** (4 nM). In agreement with previous studies,^[38]^ compounds **3**–**5** are less sensitive against the TL activity (>200 nM) and do not block the CL activity.

**Figure 4:**
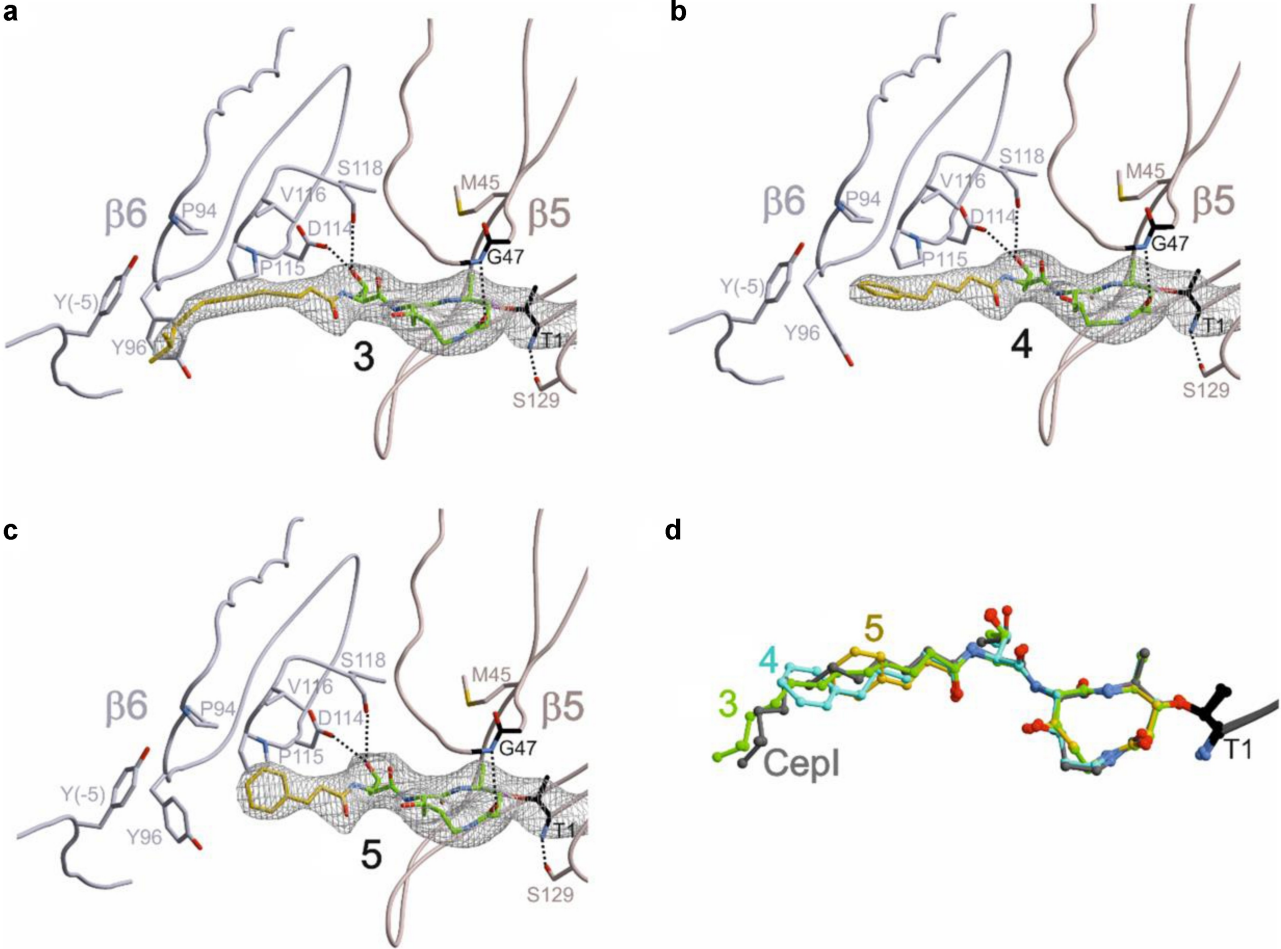
2*F*_o_−*F*_c_ electron density maps (gray mesh: 1*σ*) of a) **3** (2.9 Å resolution, PDB ID: 6ZOU), b) **4** (2.8 Å resolution, PDB ID: 6ZP6) and c) **5** (3.0 Å resolution, PDB ID: 6ZP8) bound to the β5 subunit of yCP. The dotted lines indicate hydrogen bonding. Aliphatic tails are in yellow. d) Structural superposition of **2** (CepI, gray, PDB ID: 4FZC), **3** (green), **4** (blue), and **5** (yellow) bound to the Thr1 (black) of yCP β5 subunit.

To explain the different potencies at the molecular level, compounds **3**–**5** were separately cocrystallized with the yeast 20S proteasome (yCP) and X‐ray structures were determined for yCP:**3** (2.9 Å resolution, *R*_free_=21.2 %, PDB ID: 6ZOU), yCP:**4** (2.8 Å resolution, *R*_free_=20.2 %, PDB ID: 6ZP6) and yCP:**5** (3.0 Å resolution, *R*_free_=20.6 %, PDB ID: 6ZP8). As shown,^[38]^ compounds **3**–**5** do not bind to the CL active site at β1, because the structural conformation of GLNPs displace the peptide backbone of the ligand from the proper alignment with the active site cleft. However, the electron density maps (Figure 4) reveal that **3**−**5** covalently bind to the β2 and β5 active site Thr‐1O^γ^ and develop hydrogen bonding interactions with the oxyanion hole Gly47 N (residue numbers are allocated on the basis of the alignment to the β‐subunit of *Thermoplasma acidophilum* ^[6]^). These prearrangements facilitate the Michael‐type 1,4‐reaction of the Thr‐1O^γ^ to the double bond of the GLNPs, located at C4 in the 12‐membered ring system.^[22]^ Although the β2 subunit has been attributed to TL activity, its large substrate binding pocket provides broad substrate specificity. Thus, the lower binding preference of GLNPs for this site is explained by their small P1‐alanine residues. As **3**–**5** were applied in the mM range for crystal soaking experiments, the electron density maps still display high occupancy of the respective ligands at this site. In contrast, GLNPs have the highest binding affinity to the ChTL active site at β5. The peptide moiety of each ligand adopts an antiparallel β‐sheet in this substrate binding channel. In addition, the P1‐alanine side chain forms strong hydrophobic interactions with β5‐Met45 and the P3‐threonine moiety is hydrogen bonded to Asp114 and Ser118 of the adjacent β6 subunit. While all these interactions are uniform in each of the bound GLNPs, differences between **3**−**5** are found in their individual aliphatic tails. Comparison of all three complex structures may explain the distinct ligand binding affinities to the ChTL active site: The aliphatic tail of **3** is engaged in hydrophobic interactions with Pro94, Tyr‐5 and Tyr96 forming a hydrophobic pocket in the β6 subunit (Figure 4a). Interestingly, this rigid conformation of the tail region is similar to that of **2** (cepafungin I, CepI; Figure 4d).^[23]^ Thereby, the restriction in the rotation of the highly flexible aliphatic tail region of **3**, once bound to the proteasomal active site, results in an enhanced entropic penalty and increases its IC_50_ value by sevenfold. On the other hand, the crystal structure of the yCP:**4** complex reveals that its shorter phenyl side chain is stacked between Pro94, Pro115 as well as Tyr96 of the β6 subunit (Figure 4b). However, the shorter tail region in **4** does not achieve the same accurate fitting as **3**, due to the missing interactions with β6‐Tyr‐5, thus increasing its IC_50_ value by eighteenfold compared to **2**. As expected, the yCP:**5** structure shows that the phenyl moiety of the shortest ligand does not form prominent interactions with the protein side chains (Figure 4c), which is in agreement with its low binding affinity, resulting in its 27‐fold higher IC_50_ value compared to **2**.

Taken together, inhibition of the eukaryotic proteasome might be an important ecological function of entomopathogenic bacteria like *P. laumondii*. Either GLNPs might act as toxins against the insect prey during infection or they protect the insect cadaver against other food competitors like soil‐living protozoa and amoeba. Subsequent testing of **1**–**5** against clinically relevant protozoa indeed showed their strong bioactivity with some differences in protozoa specificity (Table S13). However, cytotoxicity against mammalian L6 cells also showed their potent toxicity.

## Conclusion

The silent BGC *plu1881–1877* for GLNP production was activated both in *E. coli* by heterologous expression and in the native host *P. laumondii* by promoter exchange. The functions of Plu1881, Plu1879 and Plu1877 were investigated by separately expressing the BGC without *plu1881*, *plu1879* and *plu1877* in *E. coli* strains. Plu1877 (GlbG homologue) was identified for the first time to be involved in double bond formation of the unsaturated fatty acid moiety of GLNPs. Assisted by MS/MS molecular networking, as many as 31 derivatives were found and characterized from the promoter exchange mutant *P. laumondii* pCEP_gli, being an excellent example of applying molecular networking to map chemical diversity and biosynthetic intermediates from a culture extract. The compounds possessing cinnamic acid and cinnamalacetic acid side chains were identified as novel GLNPs, which were not generated in heterologous *E. coli*. The chemical diversity of GLNPs results from a relaxed substrate specificity for the condensation (C) domain of Plu1878 and adenylation (A) domain of Plu1880, and flexible product release from different T domains in Plu1880. The discovery of the large number of open‐ring intermediates testifies the previously proposed biosynthetic pathway for the final product glidobactin A.[^24^, ^35^] Proteasome inhibition assays combined with crystal structures of the new and main GLNPs in yeast CP suggested that the aliphatic tail, such as the degree of unsaturation and the length of chain, is vital for the high inhibitory potency. These results could be interesting for medicinal chemists to design new selective and efficient proteasome inhibitors for further drug development.

## Conflict of interest

The authors declare no conflict of interests.

## Supporting information

As a service to our authors and readers, this journal provides supporting information supplied by the authors. Such materials are peer reviewed and may be re‐organized for online delivery, but are not copy‐edited or typeset. Technical support issues arising from supporting information (other than missing files) should be addressed to the authors.

Supplementary
