# Supplementary material for: Activation, Structure, Biosynthesis and Bioactivity of Glidobactin‐like Proteasome Inhibitors from Photorhabdus laumondii
Source: Chembiochem. 2021 Mar 3;22(9):1582–8. doi: 10.1002/cbic.202100014 (PMC8248439; doi:10.1002/cbic.202100014)
Supplement: Supplementary file 1 — Supplementary [file CBIC-22-1582-s001.pdf]

ChemBioChem

Supporting Information

Activation, Structure, Biosynthesis and Bioactivity of Glidobactin-like Proteasome Inhibitors from *Photorhabdus laumondii*

Lei Zhao, Camille Le Chapelain, Alexander O. Brachmann, Marcel Kaiser, Michael Groll, and Helge B. Bode*

Table of Contents

Experimental Procedures **S5**
Strain construction S5
Strain cultivation and sample preparation S5
HPLC-MS analysis S6
Molecular networking S6
Compound isolation S6
NMR analysis S6
Quantification of production of GLNPs S7
Yeast 20S proteasome purification S7
Proteasome inhibition assays S7
Crystal growth, data collection and structure elucidation S8
Antiprotozoal activity and mammalian cell cytotoxicity assays S8
Supplementary Tables **S9**
Table S1. HR-MS data of GLNPs identified in this study S9
Table S2. ^{1}H (500 MHz) and ^{13}C (125 MHz) NMR data of **1** in DMSO-d_6 (δ in ppm) S10
Table S3. ^{1}H (500 MHz) and ^{13}C (125 MHz) NMR data of **1** in methanol-d_4 (δ in ppm) S11
Table S4. ^{1}H (500 MHz) and ^{13}C (125 MHz) NMR data of **2** in methanol-d_4 (δ in ppm) S12
Table S5. ^{1}H (500 MHz) and ^{13}C (125 MHz) NMR data of **3** in methanol-d_4 (δ in ppm) S13
Table S6. ^{1}H (500 MHz) and ^{13}C (125 MHz) NMR data of **4** in methanol-d_4 (δ in ppm) S14
Table S7. ^{1}H (500 MHz) and ^{13}C (125 MHz) NMR data of **5** in methanol-d_4 (δ in ppm) S15
Table S8. ^{1}H (500 MHz) and ^{13}C (125 MHz) NMR data of **6** in methanol-d_4 (δ in ppm) S16
Table S9. ^{1}H (500 MHz) and ^{13}C (125 MHz) NMR data of **7** in methanol-d_4 (δ in ppm) S17
Table S10. ^{1}H (500 MHz) and ^{13}C (125 MHz) NMR data of **8** in methanol-d_4 (δ in ppm) S18
Table S11. ^{1}H (500 MHz) and ^{13}C (125 MHz) NMR data of **9** in methanol-d_4 (δ in ppm) S19
Table S12. Quantification of main GLNPs produced in heterologous *E. coli* strains and *P. laumondii* pCEP_gli mutant S20
Table S13. Bioactivity of **1**−**5** against different protozoan parasites and mammalian L6 Cells S20
Table S14. X-ray data collection and refinement statistics S21
Table S15. Bacterial strains constructed and used in this study S22
Table S16. Plasmids constructed and used in this study S23
Table S17. Primers used in this study S24
Supplementary Figures **S25**
Figure S1. Production of GLNPs in (a) *E. coli plu1881−1877* and (b) *E. coli plu1881−1880* and *plu1879−1877* S25
Figure S2. Production of GLNPs in *E. coli plu1880* and *plu1879−1877* (without *plu1881*). S26
Figure S3. Production of GLNPs in *E. coli plu1881−1880* and *plu1878−1877* (without *plu1879*) S26

Figure S4. Production of GLNPs in *E. coli plu1881−1880* and *plu1879−1878* (without *plu1877*) S27
Figure S5. The overall network of molecular networking for MeOH extracts of *P. laumondii* wild type (yellow) and pCEP_gli mutant (green) S28
Figure S6. Structure confirmation of **1**−**9** by their MS/MS fragmentation patterns S32
Figure S7. Production of straight-chain fatty acid moiety containing GLNPs in *P. laumondii* Δ*bkdABC* pCEP_gli mutant. S33
Figure S8. Proposed biosynthesis for selected GLNP **2** from subclass I and GLNP **29** from subclass IV S34
Figure S9. Possible biosynthesis for cinnamalacetic acid moiety of selected GLNP **4** and cinnamic acid moiety of selected GLNP **5** from subclass II S35
Figure S10. Production of IPS and cinnamic acid and cinnamalacetic acid containing GLNPs in *P. laumondii* (a) pCEP-gli, (b) Δ*stlA* pCEP-gli, (c) Δ*stlB* pCEP-gli, and (d) Δ*stlCDE* pCEP_gli mutants S36
Figure S11. Proposed biosynthesis for selected GLNP (a) **28**, (b) **8** and (c) **6** from subclass III S37
Figure S12. ^{1}H NMR (500 MHz, DMSO-d_6) spectrum of **1** S38
Figure S13. ^{13}C NMR (500 MHz, DMSO-d_6) spectrum of **1** S39
Figure S14. ^{1}H NMR (500 MHz, methanol-d_4) spectrum of **1** S40
Figure S15. ^{13}C NMR (125 MHz, methanol-d_4) spectrum of **1** S41
Figure S16. ^{1}H NMR (500 MHz, methanol-d_4) spectrum of **2** S42
Figure S17. ^{13}C NMR (125 MHz, methanol-d_4) spectrum of **2** S43
Figure S18. ^{1}H NMR (500 MHz, methanol-d_4) spectrum of **3** S44
Figure S19. ^{13}C NMR (125 MHz, methanol-d_4) spectrum of **3** S45
Figure S20. COSY (methanol-d_4) spectrum of **3** S46
Figure S21. HSQC (methanol-d_4) spectrum of **3** S47
Figure S22. HMBC (methanol-d_4) spectrum of **3** S48
Figure S23. ^{1}H NMR (500 MHz, methanol-d_4) spectrum of **4** S49
Figure S24. ^{13}C NMR (125 MHz, methanol-d_4) spectrum of **4** S50
Figure S25. COSY (methanol-d_4) spectrum of **4** S51
Figure S26. HSQC (methanol-d_4) spectrum of **4** S52
Figure S27. HMBC (methanol-d_4) spectrum of **4** S53
Figure S28. ^{1}H NMR (500 MHz, methanol-d_4) spectrum of **5** S54
Figure S29. ^{13}C NMR (125 MHz, methanol-d_4) spectrum of **5** S55
Figure S30. COSY (methanol-d_4) spectrum of **5** S56
Figure S31. HSQC (methanol-d_4) spectrum of **5** S57
Figure S32. HMBC (methanol-d_4) spectrum of **5** S58
Figure S33. ^{1}H NMR (500 MHz, methanol-d_4) spectrum of **6** S59
Figure S34. ^{13}C NMR (125 MHz, methanol-d_4) spectrum of **6** S60

Figure S35. COSY (methanol-d_4) spectrum of **6** S61
Figure S36. HSQC (methanol-d_4) spectrum of **6** S62
Figure S37. HMBC (methanol-d_4) spectrum of **6** S63
Figure S38. ^{1}H NMR (500 MHz, methanol-d_4) spectrum of **7** S64
Figure S39. ^{13}C NMR (125 MHz, methanol-d_4) spectrum of **7** S65
Figure S40. COSY (methanol-d_4) spectrum of **7** S66
Figure S41. HSQC (methanol-d_4) spectrum of **7** S67
Figure S42. HMBC (methanol-d_4) spectrum of **7** S68
Figure S43. ^{1}H NMR (500 MHz, methanol-d_4) spectrum of **8** S69
Figure S44. ^{13}C NMR (125 MHz, methanol-d_4) spectrum of **8** S70
Figure S45. COSY (methanol-d_4) spectrum of **8** S71
Figure S46. HSQC (methanol-d_4) spectrum of **8** S72
Figure S47. HMBC (methanol-d_4) spectrum of **8** S73
Figure S48. ^{1}H NMR (500 MHz, methanol-d_4) spectrum of **9** S74
Figure S49. ^{13}C NMR (125 MHz, methanol-d_4) spectrum of **9** S75
Figure S50. COSY (methanol-d_4) spectrum of **9** S76
Figure S51. HSQC (methanol-d_4) spectrum of **9** S77
Figure S52. HMBC (methanol-d_4) spectrum of **9** S78
References S79

Experimental Procedures

Strain construction

The construction of the promoter exchange mutant *P. laumondii* pCEP_gli was carried out as described previously.[1] Briefly, the initial fragment of *plu1881* was amplified from the genomic DNA of *P. laumondii* using primers CEP_Gli_NdeI and CEP_Gli_SacI. The PCR amplicon was subcloned into vector pJET1.2 (Thermo/Fermentas) and subsequently digested and cloned into the vector pCEP-Cm via restriction sites *NdeI* and *SacI*. The resulting plasmid pCEP_gli was transformed into *E. coli* S17-1λ*pir*. For conjugation, *P. laumondii* and *E. coli* S17-1λ*pir* carrying pCEP_gli were grown in lysogeny broth (LB) medium with chloramphenicol (17 μg/mL) supplemented to *E. coli* S17-1λ*pir*. After OD_{600} 0.5–0.7, the cells were harvested and washed three times with fresh LB medium. Subsequently, the donor and recipient strains were mixed on a LB agar plate in a ratio of 1:3 and incubated at 37 °C for 3 hours followed by growth at 30 °C overnight. The next day, the bacterial cell layer was harvested and resuspended in fresh LB medium. Serial dilutions were spread out on selective LB agar plates with rifampicin (50 μg/mL) and chloramphenicol (17 μg/mL) and incubated at 30 °C for 2 days. The genotype of individual clones was verified by PCR.

The promoter exchange mutants *P. laumondii* Δ*bkdABC* pCEP_gli, Δ*stlA* pCEP_gli, Δ*stlB* pCEP_gli, and Δ*stlCDE* pCEP_gli were constructed in a similar way. *E. coli* ST18 carrying pCEP_gli was used as the donor strain with δ-aminolevulinic acid (50 μg/mL) added.[2]

For heterologous expression of *plu1881−1877* in *E. coli*, different plasmids were constructed by introducing *plu1881−1877*, *plu1880*, *plu1879−1877*, *plu1881−1880*, *plu1878−1877* and *plu1879−1878* into pFF1, pACYC, pCDF, pACYC, pCDF and pCDF to get pLZ4, pLZ5, pLZ6, pLZ7, pLZ8, and pLZ9, respectively. The correct plasmids were verified by enzyme digestion and transformed into *E. coli* DH10B MtaA. Individual clones were analyzed by HPLC-MS for the production of the glidobactin-like natural products (GLNPs).

Strain cultivation and sample preparation

100 μL of overnight cultures of *P. laumondii*, promoter exchange mutants and heterologous *E. coli* strains were inoculated into 10 mL fresh LB medium containing 2% Amberlite XAD-16 resin. Appropriate antibiotics and 0.1% L-arabinose (from a 25% stock solution) were added to LB medium when necessary. The cultures were cultivated at 30 °C and 200 rpm on a rotary

shaker. The XAD-16 beads were harvested after 3 days and extracted with 10 mL MeOH for 1 h. Subsequently, the extracts were analyzed by HPLC-MS.

HPLC-MS analysis

The HPLC-MS analysis was performed on a Dionex UltiMate 3000 system coupled to a Bruker Impact II QTOF mass spectrometer. The extracts were eluted on an ACQUITY UPLC BEH C_{18} column (130 Å, 2.1 mm × 50 mm, 1.7 μm) using a gradient from 5% to 95% MeCN/H_2O solution containing 0.1% formic acid at a flow rate of 0.4 mL/min for 16 min. Positive mode with scan range from 100 to 1200 *m*/*z* was used to detect GLNPs.

Molecular networking

The molecular network of the extracts from *P. laumondii* wide type and pCEP_gli mutant was created as described previously.[3] Briefly, the obtained HPLC-MS/MS data were converted from DataAnalysis (version 4.3, Bruker) to .mzXML files and uploaded to the Global Natural Products Social (GNPS) Molecular Networking web (https://gnps.ucsd.edu/ProteoSAFe/static/gnps-splash.jsp) to create a molecular network.[4] The default small data presets were used as the network analysis parameters except that precursor ion mass tolerance, fragment ion mass tolerance and minimum peak intensity were set to 0.05 Da, 0.01 Da and 100, respectively. The output molecular networking data were visualized using Cytoscape (version 3.6.1).

Compound isolation

To isolate GLNPs **1**−**9**, the XAD-16 resin from 4 L cultures of *P. laumondii* pCEP_gli was harvested and extracted with MeOH. The extract was fractionated by Sephadex LH-20 chromatography using MeOH as the eluent. The enriched fractions containing **1**−**9** were collected and purified by semipreparative HPLC system using gradient MeCN/H_2O solution containing 0.1% formic acid to yield **1** (23.6 mg), **2** (16.3 mg), **3** (5.6 mg), **4** (21.0 mg), **5** (3.1 mg), **6** (1.6 mg), **7** (0.9 mg), **8** (1.6 mg), and **9** (3.3 mg).

NMR analysis

1D (^{1}H and ^{13}C) and 2D (^{1}H−^{1}H-COSY, ^{1}H−^{13}C-HSQC, and ^{1}H−^{13}C-HMBC) NMR spectra were recorded on a Bruker AV 500 spectrometer at 500 MHz (^{1}H) and 125 MHz (^{13}C) using DMSO-d_6 or methanol-d_4 as solvent. The ^{1}H and ^{13}C NMR chemical shifts were referenced to the solvent peaks at δ_H 2.50 and δ_C 39.52 for DMSO-d_6 and δ_H 3.31 and δ_C 49.15 for methanol-d_4.

Quantification of production of GLNPs

Quantitative analysis of major GLNPs produced in heterologous *E. coli* strains and *P. laumondii* pCEP_gli mutant was carried out as described previously.[5] Briefly, the isolated **1** was used as standard. Its serial concentrations (50−0.78 μg/mL) were prepared and measured by HPLC-MS. The peak area at different concentrations was calculated to generate the equation $y = 4 \times 10^8x + 3 \times 10^8$ ($R^2 = 0.9955$). The extract samples from each strain were prepared as described above and analyzed by HPLC-MS. The peak area of expected compounds was obtained and their corresponding production titer was calculated based on the equation generated from **1**.

Yeast 20S proteasome purification

20S proteasome core particle (CP) from *Saccharomyces cerevisiae* (yCP) was purified as previously described.[6] To briefly summarize, yeast strains were grown in 18 L YPD medium at 30 °C into early stationary phase. Cells were harvested by centrifugation for 15 min at 5000*g* and frozen at -20 °C until further use. 120 g yeast cells were solubilized in 150 mL of 50 mM KH_2PO_4/K_2HPO_4 buffer (pH 7.5) and disrupted with a French press. Cell debris were removed by centrifugation for 30 min at 40000*g* (4 °C). The resulting supernatant was filtered and saturated aqueous $(NH_4)_2SO_4$ was added to a final concentration of 30% (v/v). This solution was loaded on a Phenyl Sepharose™ 6 Fast Flow column (GE Healthcare) preequilibrated with 1 M $(NH_4)_2SO_4$ in 20 mM KH_2PO_4/K_2HPO_4 (pH 7.5). yCP was eluted by applying a linear gradient from 1 to 0 M $(NH_4)_2SO_4$. Proteasome-containing fractions were pooled and loaded onto a hydroxyapatite column (BioRad) equilibrated with 20 mM KH_2PO_4/K_2HPO_4 (pH 7.5). yCP was eluted by applying a phosphate buffer gradient (20 to 500 mM). After anion exchange chromatography (Resource Q column, GE Healthcare) with gradient elution (0−500 mM NaCl in 20 mM Tris/HCl, pH 7.5), yCP was subjected to size exclusion chromatography on a Superose 6 10/300 GL (GE Healthcare) using 150 mM NaCl in 20 mM Tris/HCl (pH 7.5). The protein was concentrated to 40 mg/mL in 20 mM Tris/HCl (pH 7.5) and stored at 4 °C for further use.

Proteasome inhibition assays

In vitro proteasome inhibition assays were performed by fluorescence assays in 96-well plates. Assay mixtures contained 10 μg/mL of purified yCP in 20 mM Tris/HCl buffer (pH 7.5) containing 0.01% (w/v) SDS. Inhibitors were dissolved in DMSO and added at various concentrations. Assays were conducted in triplicates. A sample containing DMSO served as vehicle control. After an incubation time of 60 min at room temperature, the fluorogenic substrate

Suc-Leu-Leu-Val-Tyr-AMC (Bachem) was added to a final concentration of 333 μM in order to measure the residual activity of the chymotrypsin-like (ChTL) site. The assay mixture was incubated for another hour at room temperature, then diluted with 300 μL of 20 mM Tris/HCl (pH 7.5). Fluorescence was measured on a Varian Cary Eclipse photofluorometer with excitation and emission wavelengths of λ(excitation) = 360 nm and λ(emission) = 460 nm. The fluorescence values were normalized to the DMSO control. The IC_{50} values were obtained by plotting the percent inhibition against inhibitor concentration [I] and fitting the experimental data to the following equation: % inhibition = $100 \times [I]_0/(IC_{50} + [I]_0)$.

Crystal growth, data collection and structure elucidation

Crystals were grown in hanging drop plates at 20 °C as previously described,[6] using a protein concentration of 40 mg/mL in 20 mM Tris/HCl (pH 7.5). The drops contained 1 μL of protein and 1 μL of the reservoir solution consisting of 25 mM $Mg(OAc)_2$, 100 mM MES (pH 6.8) and 10% (v/v) 2-methyl-2,4-pentanediol (MPD). Crystals were soaked with the respective inhibitors in DMSO at final concentrations of 2 mM for at least 24 h following complementation of the droplets with cryoprotecting buffer consisting of 30% (w/v) MPD, 20 mM $Mg(OAc)_2$, 100 mM MES (pH 6.8). Crystals were supercooled in a stream of liquid nitrogen gas at 100 K (Oxford Cryo Systems). Datasets of proteasome:inhibitor complexes were collected up to 2.5 Å resolution using synchrotron radiation (λ = 1.0 Å) at the X06SA-beamline (Swiss Light Source, Villingen, Switzerland, Table S14). X-ray intensities were assessed with the program XDS and data reduction was carried out using XSCALE.[7] Molecular replacement started with the coordinates of yCP (PDB ID: 5CZ4)[8] and Translation/Libration/Screw (TLS) refinements were performed with REFMAC5 in the CCP4i suite.[9] Structures were built with the programs MAIN[10] and COOT.[11] The amino acids numbering in the manuscript follows the primary sequence alignment of *Thermoplasma acidophilum*.[12]

Antiprotozoal activity and mammalian cell cytotoxicity assays

Bioactivity of **3**–**5** against the parasites *Trypanosoma brucei rhodesiense* STIB900, *Trypanosoma cruzi* Tulahuen C4, *Leishmania donovani* MHOM-ET-67/L82 and *Plasmodium falciparum* NF54 and their cytotoxicity against rat skeletal myoblasts (L6 cells) were evaluated as described previously.[13]

Supplementary Tables

Table S1. HR-MS data of GLNPs identified in this study

Compound	Sum formula	Found $[M + H]^+$	Calcd. $[M + H]^+$	Δppm
**1**	$C_{27}H_{44}N_4O_6$	521.3322	521.3334	2.2
**2**	$C_{28}H_{46}N_4O_6$	535.3478	535.3490	2.3
**3**	$C_{28}H_{50}N_4O_6$	539.3797	539.3803	1.2
**4**	$C_{26}H_{34}N_4O_6$	499.2544	499.2551	1.5
**5**	$C_{24}H_{32}N_4O_6$	473.2390	473.2395	1.0
**6**	$C_{27}H_{48}N_4O_8$	557.3530	557.3545	2.6
**7**	$C_{28}H_{50}N_4O_8$	571.3686	571.3701	2.6
**8**	$C_{25}H_{44}N_4O_7$	513.3269	513.3283	2.7
**9**	$C_{26}H_{46}N_4O_7$	527.3423	527.3439	3.1
**10**	$C_{30}H_{52}N_4O_6$	565.3951	565.3960	1.5
**11**	$C_{28}H_{48}N_4O_6$	537.3629	537.3647	3.3
**12**	$C_{29}H_{52}N_4O_6$	553.3949	553.3960	1.8
**13**	$C_{30}H_{54}N_4O_6$	567.4104	567.4116	2.1
**14**	$C_{27}H_{48}N_4O_6$	525.3634	525.3647	2.5
**15**	$C_{26}H_{46}N_4O_6$	511.3480	511.3490	2.0
**16**	$C_{24}H_{42}N_4O_6$	483.3166	483.3177	2.2
**17**	$C_{26}H_{36}N_4O_6$	501.2697	501.2708	2.1
**18**	$C_{26}H_{38}N_4O_8$	535.2755	535.2762	1.4
**19**	$C_{24}H_{34}N_4O_7$	491.2492	491.2500	1.7
**20**	$C_{22}H_{32}N_4O_7$	465.2337	465.2344	1.4
**21**	$C_{28}H_{54}N_4O_8$	575.4002	575.4014	2.1
**22**	$C_{26}H_{48}N_4O_7$	529.3584	529.3596	2.3
**23**	$C_{28}H_{52}N_4O_7$	557.3898	557.3909	2.0
**24**	$C_{25}H_{48}N_4O_7$	517.3584	517.3596	2.3
**25**	$C_{24}H_{46}N_4O_7$	503.3430	503.3439	1.8
**26**	$C_{22}H_{42}N_4O_7$	475.3119	475.3126	1.5
**27**	$C_{26}H_{50}N_4O_7$	531.3742	531.3752	2.0
**28**	$C_{23}H_{45}N_3O_6$	460.3376	460.3381	1.2
**29**	$C_{28}H_{46}N_4O_5$	519.3523	519.3541	3.5
**30**	$C_{28}H_{50}N_4O_5$	523.3848	523.3854	1.2
**31**	$C_{28}H_{54}N_4O_7$	559.4054	559.4065	2.0
**32**	$C_{22}H_{39}N_3O_6$	442.2903	442.2912	1.9
**33**	$C_{27}H_{48}N_4O_7$	541.3580	541.3596	2.9
**34**	$C_{25}H_{44}N_4O_6$	497.3322	497.3334	2.3
**35**	$C_{27}H_{46}N_4O_6$	523.3478	523.3490	2.3
**36**	$C_{27}H_{44}N_4O_5$	505.3376	505.3384	1.7
**37**	$C_{25}H_{46}N_4O_7$	515.3426	515.3439	2.6
**38**	$C_{27}H_{52}N_4O_7$	545.3891	545.3909	3.2

Table S2. ^{1}H (500 MHz) and ^{13}C (125 MHz) NMR data^a of **1** in DMSO-d$_6$ (δ in ppm)

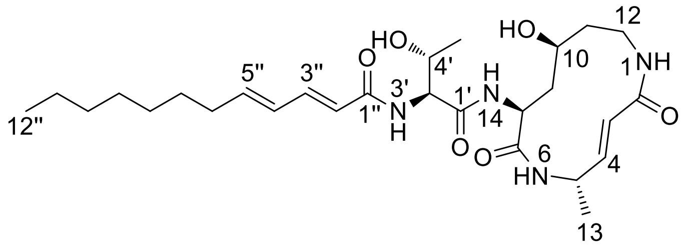

Position	δ_C, type	δ_H, mult. (J in Hz)
1-NH		7.41, t (6.1)
2	167.8, C	
3	123.3, CH	6.19, overlap
4	143.3, CH	6.41, d (11.7)
5	44.8, CH	4.32, m
6-NH		8.65, s
7	171.1, C	
8	51.3, CH	4.32, m
9	42.4, CH_2	1.85, m; 1.58, d (11.5)
10	66.8, CH	3.57, m
11	39.9, CH_2^{b}	1.45, m
12	40.1, CH_2^{b}	3.01, m
13	18.6, CH_3	1.25, overlap
14-NH		7.74, d (7.7)
1'	169.5, C	
2'	58.2, CH	4.32, overlap
3'-NH		7.88, d (8.5)
4'	67.0, CH	3.94, t (11.2)
5'	20.0, CH_3	1.00, d (6.3)
1"	165.6, C	
2"	123.1, CH	6.10, overlap
3"	139.9, CH	7.00, dd (15.1, 10.7)
4"	128.6, CH	6.19, óverlap
5"	142.3, CH	6.10, overlap
6"	32.3, CH_2	2.12, q (7.0)
7"	28.4, CH_2	1.37, m
8"	28.6, CH_2	1.25, m
9"	28.6, CH_2	1.25, m
10"	31.3, CH_2	1.25, m
11"	22.1, CH_2	1.25, m
12"	14.0, CH_3	0.85, t (6.7)

^{a}Identical with reported data[14,15], ^{b}submerged in solvent

Table S3. ^{1}H (500 MHz) and ^{13}C (125 MHz) NMR data of **1** in methanol-d$_4$ (δ in ppm)

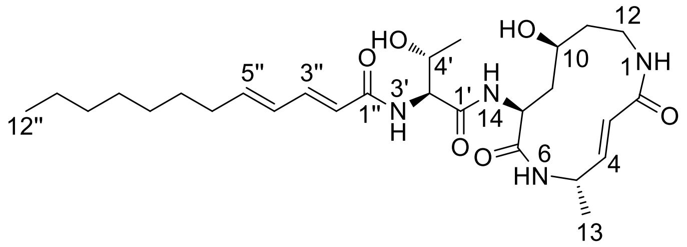

Position	δ_C, type	δ_H, mult. (J in Hz)
2	171.5, C	
3	124.2, CH	6.36, d (15.9)
4	146.0, CH	6.63, d (11.4)
5	47.1, CH	4.57, m
7	173.6, C	
8	53.5, CH	4.41, d (4.3)
9	42.4, CH_2	2.10, 1.79, m
10	69.1, CH	3.73, m
11	40.6, CH_2	1.68, 1.59, m
12	41.4, CH_2	3.20, m
13	18.9, CH_3	1.32, m
1'	172.2, C	
2'	60.2, CH	4.41, d (4.3)
4'	68.7, CH	4.13, m
5'	20.3, CH_3	1.17, d (6.4)
1''	169.4, C	
2''	122.6, CH	6.07, d (15.1)
3''	143.3, CH	7.16, dd (15.1, 10.7)
4''	130.0, CH	6.23, dd (15.0, 10.8)
5''	144.9, CH	6.14, dd (14.6, 7.4)
6''	34.1, CH_2	2.18, q (7.1)
7''	30.1, CH_2	1.45, m
8''	30.4, CH_2	1.32, m
9''	30.4, CH_2	1.32, m
10''	33.1, CH_2	1.32, m
11''	23.9, CH_2	1.32, m
12''	14.6, CH_3	0.90, t (7.0)

Table S4. ^{1}H (500 MHz) and ^{13}C (125 MHz) NMR data of **2** in methanol-d$_4$ (δ in ppm)

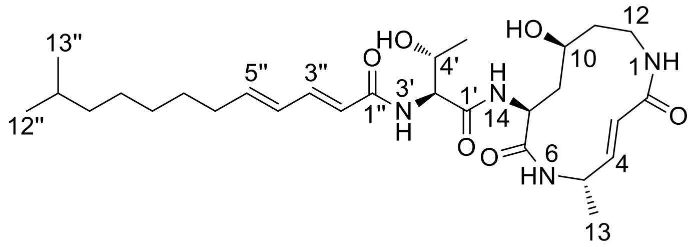

Position	δ_C, type	δ_H, mult. (J in Hz)
2	171.6, C	
3	124.2, CH	6.36, d (15.9)
4	146.1, CH	6.63, d (11.5)
5	47.1, CH	4.57, m
7	173.6, C	
8	53.5, CH	4.41, d (4.15)
9	42.6, CH_2	2.10, 1.79, m
10	69.0, CH	3.73, m
11	40.6, CH_2	1.68, 1.60, m
12	41.4, CH_2	3.20, m
13	18.9, CH_3	1.32, m
1'	172.2, C	
2'	60.2, CH	4.41, d (4.15)
4'	68.7, CH	4.13, m
5'	20.3, CH_3	1.17, m
1"	169.4, C	
2"	122.6, CH	6.07, d (15.1)
3"	143.3, CH	7.16, dd (15.1, 10.7)
4"	130.0, CH	6.23, dd (15.1, 10.8)
5"	144.9, CH	6.14, dd (14.6, 7.4)
6"	34.1, CH_2	2.18, q (7.1)
7"	30.1, CH_2	1.45, m
8"	30.7, CH_2	1.32, m
9"	28.5, CH_2	1.32, m
10"	40.3, CH_2	1.17, m
11"	29.3, CH	1.52, m
12"	23.2, CH_3	0.88, d (6.6)
13"	23.2, CH_3	0.88, d (6.6)

Table S5. ^{1}H (500 MHz) and ^{13}C (125 MHz) NMR data of **3** in methanol-d$_4$ (δ in ppm). COSY (bold) and key HMBC (arrows) are shown

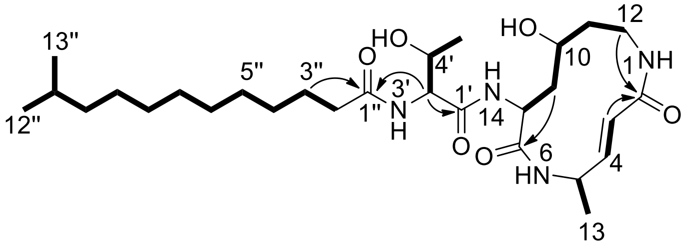

Position	δ_C, type	δ_H, mult. (J in Hz)
2	171.6, C	
3	124.2, CH	6.36, dd (15.9, 0.6)
4	146.1, CH	6.63, d (10.9)
5	47.1, CH	4.58, submerged
7	173.6, C	
8	53.5, CH	4.41, d (9.6)
9	42.5, CH_2	2.10, 1.79, m
10	69.0, CH	3.73, m
11	40.6, CH_2	1.64, m
12	41.4, CH_2	3.20, m
13	18.9, CH_3	1.32, m
1'	172.3, C	
2'	60.0, CH	4.32, d (4.2)
4'	68.6, CH	4.11, m
5'	20.3, CH_3	1.17, m
1"	176.8, C	
2"	37.1, CH_2	2.30, m
3"	27.1, CH_2	1.64, m
4"	30.5, CH_2	1.32, m
5"	30.7, CH_2	1.32, m
6"	30.9, CH_2	1.32, m
7"	30.8, CH_2	1.32, m
8"	31.2, CH_2	1.32, m
9"	28.7, CH_2	1.32, m
10"	40.4, CH_2	1.17, m
11"	29.3, CH	1.52, m
12"	23.2, CH_3	0.88, d (6.6)
13"	23.2, CH_3	0.88, d (6.6)

Table S6. ^{1}H (500 MHz) and ^{13}C (125 MHz) NMR data of **4** in methanol-d$_4$ (δ in ppm). COSY (bold) and key HMBC (arrows) are shown

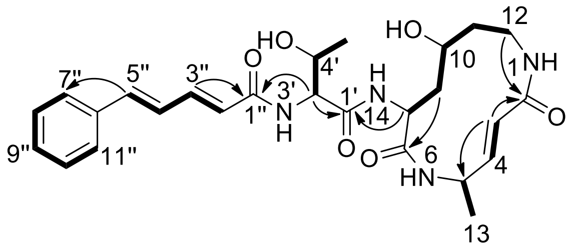

Position	δ_C, type	δ_H, mult. (J in Hz)
2	171.6, C	
3	124.2, CH	6.36, dd (15.9, 1.0)
4	146.1, CH	6.63, m
5	47.1, CH	4.59, submerged
7	173.6, C	
8	53.6, CH	4.43, overlap
9	42.6, CH_2	2.11, 1.81, m
10	69.0, CH	3.73, m
11	40.6, CH_2	1.67, 1.60, m
12	41.3, CH_2	3.20, m
13	18.8, CH_3	1.34, d (4.5)
1'	172.2, C	
2'	60.3, CH	4.43, overlap
4'	68.7, CH	4.15, m
5'	20.3, CH_3	1.19, m
1"	169.1, C	
2"	124.7, CH	6.30, d (15.0)
3"	143.0, CH	7.36, m
4"	127.8, CH	7.01, dd (15.5, 10.5)
5"	141.0, CH	6.94, d (15.6)
6"	137.9, C	
7"	128.3, CH	7.52, m
8"	130.0, CH	7.36, m
9"	130.0, CH	7.29, dd (8.3, 6.4)
10"	130.0, CH	7.36, m
11"	128.3, CH	7.52, m

Table S7. ^{1}H (500 MHz) and ^{13}C (125 MHz) NMR data of **5** in methanol-d$_4$ (δ in ppm). COSY (bold) and key HMBC (arrows) are shown

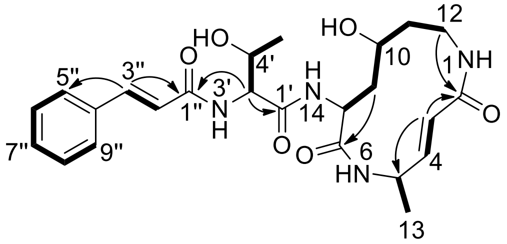

Position	δ_C, type	δ_H, mult. (J in Hz)
2	171.5, C	
3	124.2, CH	6.36, dd (15.9, 0.7)
4	146.1, CH	6.62, d (12.3)
5	47.1, CH	4.56, br. s
7	173.6, C	
8	53.6, CH	4.43, d (10.4)
9	42.5, CH_2	2.11, 1.81, m
10	69.1, CH	3.73, m
11	40.6, CH_2	1.67, 1.60, m
12	41.4, CH_2	3.20, m
13	18.9, CH_3	1.34, d (5.2)
1'	172.2, C	
2'	60.3, CH	4.46, d (4.3)
4'	68.7, CH	4.17, m
5'	20.3, CH_3	1.20, d (6.4)
1''	168.9, C	
2''	121.6, CH	6.80, d (15.8)
3''	142.7, CH	7.56, overlap
4''	136.4, C	
5''	129.1, CH	7.58, overlap
6''	130.1, CH	7.38, overlap
7''	131.1, CH	7.38, overlap
8''	130.1, CH	7.38, overlap
9''	129.1, CH	7.58, overlap

Table S8. ^{1}H (500 MHz) and ^{13}C (125 MHz) NMR data of **6** in methanol-d$_4$ (δ in ppm). COSY (bold) and key HMBC (arrows) are shown

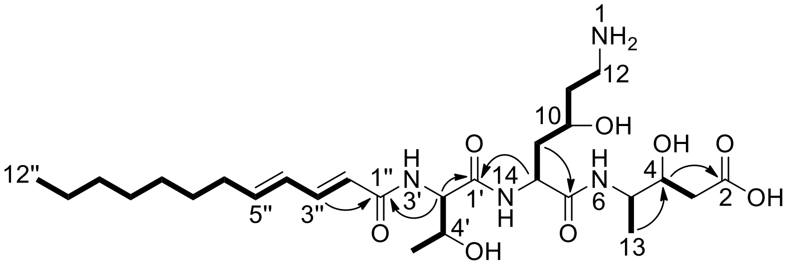

Position	δ_C, type	δ_H, mult. (J in Hz)
2	175.7, C	
3	40.0, CH_2	2.45, 2.36, m
4	71.5, CH	3.99, m
5	50.5, CH	3.99, m
7	173.4, C	
8	52.3, CH	4.51, dd (8.4, 6.3)
9	40.5, CH_2	2.02, 1.87, m
10	68.0, CH	3.85, m
11	34.8, CH_2	1.87, 1.70, m
12	38.7, CH_2	3.07, m
13	17.3, CH_3	1.16, d (6.8)
1'	172.8, C	
2'	60.9, CH	4.34, d (4.5)
4'	68.5, CH	4.16, m
5'	20.2, CH_3	1.22, d
1''	169.7, C	
2''	122.5, CH	6.09, d (15.1)
3''	143.4, CH	7.16, dd (15.1, 10.7)
4''	129.9, CH	6.23, dd (15.1, 10.7)
5''	145.1, CH	6.14, dd (14.6, 7.4)
6''	34.1, CH_2	2.18, m
7''	30.1, CH_2	1.44, m
8''	30.4, CH_2	1.31, m
9''	30.4, CH_2	1.31, m
10''	33.1, CH_2	1.31, m
11''	23.9, CH_2	1.31, m
12''	14.6, CH_3	0.90, t (6.9)

Table S9. ^{1}H (500 MHz) and ^{13}C (125 MHz) NMR data of **7** in methanol-d_4 (δ in ppm). COSY (bold) and key HMBC (arrows) are shown

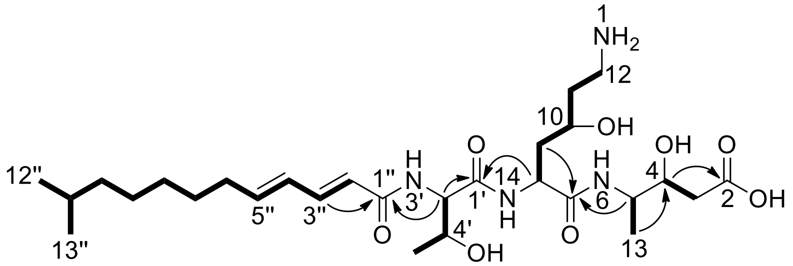

Position	δ_C, type[a]	δ_H, mult. (J in Hz)
2	175.6, C	
3	40.0, CH_2	2.46 dd (15.8, 4.3), 2.37, dd (15.7, 8.8)
4	71.5, CH	3.99, m
5	50.5, CH	3.99, m
7	173.4, C	
8	52.3, CH	4.52, m
9	40.5, CH_2	2.02, 1.87, m
10	68.0, CH	3.84, m
11	34.8, CH_2	1.87, 1.70, m
12	38.7, CH_2	3.07, m
13	17.3, CH_3	1.17, overlap
1'	172.8, C	
2'	60.9, CH	4.34, d (4.5)
4'	68.5, CH	4.16, m
5'	20.2, CH_3	1.23, d (6.4)
1"	169.7, C	
2"	122.5, CH	6.10, d (15.3)
3"	143.4, CH	7.16, dd (15.1, 10.6)
4"	129.9, CH	6.24, dd (15.1, 10.8)
5"	145.1, CH	6.15, dd (14.5, 7.4)
6"	34.1, CH_2	2.19, q (7.0)
7"	30.1, CH_2	1.45, m
8"	30.7, CH_2	1.31, m
9"	28.5, CH_2	1.31, m
10"	40.3, CH_2	1.17, overlap
11"	29.3, CH	1.52, m
12"	23.2, CH_3	0.88, dd (6.6, 1.1)
13"	23.2, CH_3	0.88, dd (6.6, 1.1)

[a]Some ^{13}C NMR data were obtained inversely from ^{1}H–^{13}C HSQC and ^{1}H–^{13}C HMBC data

Table S10. ^{1}H (500 MHz) and ^{13}C (125 MHz) NMR data of **8** in methanol-d4 (δ in ppm). COSY (bold) and key HMBC (arrows) are shown

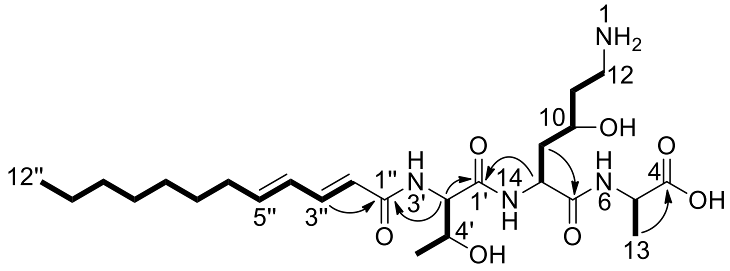

Position	δ_C, type	δ_H, mult. (J in Hz)
4	176.1, C	
5	49.5, CH	4.36, m
7	173.5, C	
8	51.8, CH	4.57, m
9	40.7, CH_2	2.02, 1.89, m
10	67.9, CH	3.89, m
11	34.8, CH_2	1.89, 1.70, m
12	38.7, CH_2	3.07, m
13	17.7, CH_3	1.41, d (7.4)
1'	172.6, C	
2'	60.7, CH	4.36, m
4'	68.5, CH	4.14, m
5'	20.1, CH_3	1.21, d (6.4)
1"	169.6, C	
2"	122.5, CH	6.09, d (15.3)
3"	143.3, CH	7.15, dd (15.1, 10.7)
4"	129.9, CH	6.23, dd (15.1, 10.8)
5"	145.0, CH	6.14, dd (14.7, 7.4)
6"	34.1, CH_2	2.18, dd (14.5, 7.2)
7"	30.1, CH_2	1.44, m
8"	30.4, CH_2	1.31, m
9"	30.4, CH_2	1.31, m
10"	33.1, CH_2	1.31, m
11"	23.8, CH_2	1.31, m
12"	14.6, CH_3	0.90, t (7.0)

Table S11. ^{1}H (500 MHz) and ^{13}C (125 MHz) NMR data of **9** in methanol-d$_4$ (δ in ppm). COSY (bold) and key HMBC (arrows) are shown

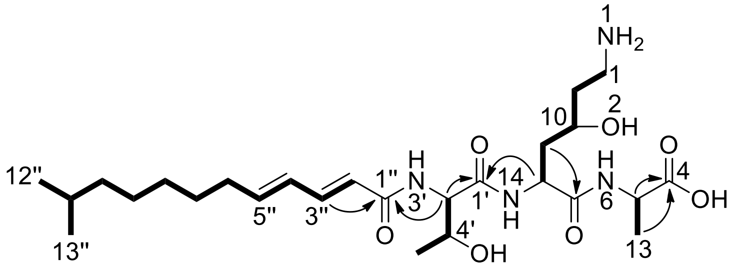

Position	δ_C, type	δ_H, mult. (J in Hz)
4	176.6, C	
5	49.9, CH	4.35, m
7	173.4, C	
8	51.9, CH	4.57, dd (8.1, 6.7)
9	40.7, CH_2	2.02, 1.87, m
10	67.8, CH	3.89, m
11	34.8, CH_2	1.87, 1.70, m
12	38.7, CH_2	3.07, m
13	17.8, CH_3	1.40, d (7.3)
1'	172.6, C	
2'	60.7, CH	4.35, m
4'	68.5, CH	4.14, m
5'	20.2, CH_3	1.21, d (6.4)
1"	169.6, C	
2"	122.6, CH	6.09, d (15.1)
3"	143.3, CH	7.15, dd (15.1, 10.7)
4"	130.0, CH	6.23, dd (15.1, 10.8)
5"	145.0, CH	6.14, dd (14.6, 7.4)
6"	34.1, CH_2	2.18, q (7.0)
7"	30.2, CH_2	1.45, m
8"	30.7, CH_2	1.31, m
9"	28.5, CH_2	1.31, m
10"	40.3, CH_2	1.18, m
11"	29.3, CH	1.52, m
12"	23.2, CH_3	0.88, d (6.6)
13"	23.2, CH_3	0.88, d (6.6)

Table S12. Quantification of main GLNPs produced in heterologous *E. coli* strains and *P. laumondii* pCEP_gli mutant

Strain	GLNP (mg/L)							
	**1**	**2**	**3**	**4**	**5**	**8**	**34**	**36**
E. coli plu1881−1877	2.9	−	−	−	−	1.6	−	−
E. coli plu1881−1880 and *plu1879−1877*	1.1	−	−	−	−	1.5	−	−
E. coli plu1880 and *plu1879−1877*	−	−	−	−	−	−	2.2	1.3
E. coli plu1881−1880 and *plu1878−1877*	0.7	−	−	−	−	0.9	−	−
P. laumondii pCEP_gli	2.6	2.9	2.7	7.2	2.9	−	−	−

Table S13. Bioactivity of **1**−**5** against different protozoan parasites and mammalian L6 Cells

Species	IC_{50} (μM)				
	**1**	**2**	**3**	**4**	**5**
T. brucei rhodesiense	0.02	0.14	0.44	1.4	8.5
T. cruzi	34	1.3	0.68	79	>100
L. donovani	0.76	4.5	0.27	21	>100
P. falciparum	0.26	0.60	0.33	1.3	8.8
rat skeletal myoblasts	0.15	0.12	0.05	11	65

Table S14. X-ray data collection and refinement statistics

Crystallographic data	yCP:**3** (HB333)	yCP:**4** (HB334)	yCP:**5** (HB335)
**Crystal parameters**			
Space group	$P2_1$	$P2_1$	$P2_1$
Cell constants (Å)/°	a = 136.8	a = 135.7	a = 136.2
	b = 300.2	b = 301.6	b = 300.1
	c = 145.8	c = 144.4	c = 144.8
	β = 113.3	β = 113.1	β = 113.1
CPs/AU[a]	1	1	1
**Data collection**			
Beam line	X06SA, SLS	X06SA, SLS	X06SA, SLS
Wavelength (Å)	1.0	1.0	1.0
Resolution range (Å)[b]	50−2.9 (3.0−2.9)	50−2.8 (2.9−2.8)	50−3.0 (3.1−3.0)
No. observations	714999	809866	656915
No. unique reflections[c]	231040	255091	210818
Completeness (%)[b]	97.0 (99.2)	97.5 (98.4)	99.0 (99.7)
R_{merge} (%)[b, d]	8.3 (56.2)	6.1 (54.3)	9.5 (52.0)
I/σ (I)[b]	11.7 (2.5)	13.4 (2.6)	10.9 (2.4)
**Refinement (REFMAC5)**			
Resolution range (Å)	15−2.9	15−2.8	15−3.0
No. reflections working set	217934	240805	198714
No. reflections test set	11470	12674	10458
No. nonhydrogen atoms	49820	49853	49805
No. of ligand atoms	152	144	136
Solvent (H_2O, ions, MES)	325	376	336
R_{work}/R_{free} (%)[e]	14.4/21.2	17.4/20.2	16.7/20.6
rmsd bond (Å)/(°)[f]	0.007/1.1	0.006/1.1	0.006/1.1
Average B-factor (Å^2)	74.3	70.2	76.1
Ramachandran plot (%)[g]	98.0/1.7/0.3	98.0/1.7/0.3	97.9/1.8/0.3
PDB ID	6ZOU	6ZP6	6ZP8

[a]Asymmetric unit
[b]The values in parentheses for resolution range, completeness, R_{merge} and I/σ (I) correspond to the highest resolution shell
[c]Data reduction was carried out with XDS[16] and from a single crystal. Friedel pairs were treated as identical reflections
[d]$R_{merge}(I) = \Sigma_{hkl}\Sigma_j \mid I(hkl)_j - \langle I(hkl)\rangle \mid / \Sigma_{hkl} \Sigma_j I(hkl)_j$, where $I(hkl)_j$ is the jth measurement of the intensity of reflection hkl and <I(hkl)> is the average intensity
[e]$R = \Sigma_{hkl} \mid |F_{obs}| - |F_{calc}| \mid / \Sigma_{hkl} |F_{obs}|$, where R_{free} is calculated without a sigma cut off for a randomly chosen 5% of reflections, which were not used for structure refinement, and R_{work} is calculated for the remaining reflections
[f]Deviations from ideal bond lengths/angles
[g]Number of residues in favored region/allowed region/outlier region

Table S15. Bacterial strains constructed and used in this study

Strain	Genotype/Description	Reference
E. coli DH10B MtaA	F-*mcr*A, Δ(*mrr*-*hsd*RMS-*mcr*BC), Φ80*lac*ZΔM15, Δ*lac*X74, *rec*A1, *end*A1, *ara*D139, Δ(*ara leu*)7697, *gal*U, *gal*K, *rps*L, *nup*G, λ–, *ent*D::*mtaA*	[17]
E. coli S17-1λ*pir*	Tp^r Sm^r *recA thi hsdR* RP4-2-Tc::MuKm::Tn7, λ*pir*	[18]
E. coli ST18	*E. coli* S17-1 λ*pir* Δ*hemA*	[19]
P. laumondii	wild type	[20,21]
P. laumondii Δ*bkdABC*	*bkdABC* (*plu1883-1885*) deletion mutant	Bode lab
P. laumondii Δ*stlA*	Δ*stlA* (*plu2234*) deletion mutant	Bode lab
P. laumondii Δ*stlB*	Δ*stlB* (*plu2134*) deletion mutant	Bode lab
P. laumondii Δ*stlCDE*	Δ*stlCDE* (*plu2163-2165*) deletion mutant	Bode lab
P. laumondii pCEP_gli	*P. laumondii* with a promoter exchange in front of *plu1881*	this work
P. laumondii Δ*bkdABC* pCEP_gli	*P. laumondii* Δ*bkdABC* with a promoter exchange in front of *plu1881*	this work
P. laumondii Δ*stlA* pCEP_gli	*P. laumondii* Δ*stlA* with a promoter exchange in front of *plu1881*	this work
P. laumondii Δ*stlB* pCEP_gli	*P. laumondii* Δ*stlB* with a promoter exchange in front of *plu1881*	this work
P. laumondii Δ*stlCDE* pCEP_gli	*P. laumondii* Δ*stlCDE* with a promoter exchange in front of *plu1881*	this work
E. coli plu1881−1877	*E. coli* DH10B MtaA expressing *plu1881−1877* (pLZ4)	this work
E. coli plu1881−1880 and *plu1879−1877*	*E. coli* DH10B MtaA coexpressing *plu1881−1880* (pLZ7) and *plu1879−1877* (pLZ6)	this work
E. coli plu1880 and *plu1879−1877*	*E. coli* DH10B MtaA coexpressing *plu1880* (pLZ5) and *plu1879−1877* (pLZ6)	this work
E. coli plu1881−1880 and *plu1878−1877*	*E. coli* DH10B MtaA coexpressing *plu1881−1880* (pLZ7) and *plu1878−1877* (pLZ8)	this work
E. coli plu1881−1880 and *plu1879−1878*	*E. coli* DH10B MtaA coexpressing *plu1881−1880* (pLZ7) and *plu1879−1878* (pLZ9)	this work

Table S16. Plasmids constructed and used in this study

Plasmid	Genotype/Description	Reference
pACYC	modified from pACYC_tacI/I containing arabinose-inducible promoter and chloramphenicol resistance gene (Cm^R)	Bode lab
pCDF	modified from pCDF_tacI/I containing arabinose-inducible promoter and spectinomycin resistance gene (Sm^R)	Bode lab
pFF1	2µ ori, $G418^R$, P_{BAD} promoter, pCOLA ori, MCS, Ypet-Flag, Km^R	[22]
pCEP-Cm	R6Kγ ori, oriT, *araC*, *araBAD* promoter, Cm^R	[1]
pCEP_gli	initial fragment of *plu1881* from *P. laumondii* assembled into pCEP-Cm, CmR	this work
pLZ4	*plu1881−1877* from *P. laumondii* assembled into pFF1, Km^R	this work
pLZ5	*plu1880* from *P. laumondii* assembled into pACYC, Cm^R	this work
pLZ6	*plu1879−1877* from *P. laumondii* assembled into pCDF, Sm^R	this work
pLZ7	*plu1881−1880* from *P. laumondii* assembled into pACYC, Cm^R	this work
pLZ8	*plu1878−1877* from *P. laumondii* assembled into pCDF, Sm^R	this work
pLZ9	*plu1879−1878* from *P. laumondii* assembled into pCDF, SmR	this work

Table S17. Primers used in this study. Restriction sites used for cloning are underlined

Primer	5' to 3' Sequence	Targeting DNA fragment	Plasmid
CEP_Gli_NdeI	CATATGGGCTGGAATATATTTATTAACGC	initial fragment of *plu1881* from *P. laumondii*	pCEP_gli
CEP_Gli_SacI	GAGCTCGTCACGATGAAAACAGTTAGGC		
LZ_10	TCGCAACTCTCTACTGTTTCTCCATACCCGTTTTTTTGGGCTAACAGGAGGAATTCCATGGGCTGGAATATATTTATTAACGC	fragment I of *plu1881−1877* from *P. laumondii*	pLZ4
LZ_12	CAGACTAAGACGCTGACACAGAG		
LZ_11	CCGAGGTGATCGAGTTGG	fragment II of *plu1881−1877* from *P. laumondii*	
LZ_14	GCTGATGTGACGTGCCAG		
LZ_13	CGGAAGAGACGACAGAAGG	fragment III of *plu1881−1877* from *P. laumondii*	
LZ_15	CTTCACCTTTGCTCATGAACTCGCCAGAACCAGCAGCGGAGCCAGCGGATCCGGCGCGCCTTAATGAGGTACTTCAAATTTAAAGTAATCG		
LZ_17	ATTCCTTGCCAACGCCGGCTCAAC	fragment I of *plu1880* from *P. laumondii*	pLZ5
LZ_18	TCTAGCAGTTCACGCCAGATAG		
LZ_19	AGTTGTGTCATCACTCAGTCGC	fragment II of *plu1880* from *P. laumondii*	
LZ_20	TCATATCTGTCCTCCTGTTATTATTGATG		
LZ_21	TCCATCAATAATAACAGGAGGACAGATATGACAATTAATCATCGGCTCGTATAATG	pACYC vector backbone	
LZ_22	AGCCATAGCCTGCGTTGAGCCGGCGTTGGCAAGGAATTCCTCCTGTTAGCCC		
LZ_23	ATGAGTGATTCTTCCCCAACG	*plu1879−1877* from *P. laumondii*	pLZ6
LZ_24	TTAATGAGGTACTTCAAATTTAAAGTAATCG		
LZ_25	AGCGATTACTTTAAATTTGAAGTACCTCATTAACAATTAATCATCGGCTCGTATAATG	pCDF vector backbone	
LZ_26	TCAGGAAGGTGAACCGTTGGGGAAGAATCACTCATGGAATTCCTCCTGTTAGCCC		
LZ_27	ATGGGCTGGAATATATTTATTAACGC	fragment I of *plu1881−1880* from *P. laumondii*	pLZ7
LZ_18	TCTAGCAGTTCACGCCAGATAG		
LZ_19	AGTTGTGTCATCACTCAGTCGC	fragment II of *plu1881−1880* from *P. laumondii*	
LZ_20	TCATATCTGTCCTCCTGTTATTATTGATG		
LZ_21	TCCATCAATAATAACAGGAGGACAGATATGACAATTAATCATCGGCTCGTATAATG	pACYC vector backbone	
LZ_28	TGGTGCGTTAATAAATATATTCCAGCCCATGGAATTCCTCCTGTTAGCCC		
LZ_29	ATGAAACAACATCAAGGAAGCTATTAC	*plu1878−1877* from *P. laumondii*	pLZ8
LZ_24	TTAATGAGGTACTTCAAATTTAAAGTAATCG		
LZ_25	AGCGATTACTTTAAATTTGAAGTACCTCATTAACAATTAATCATCGGCTCGTATAATG	pCDF vector backbone	
LZ_30	TCAGGGGGTAATAGCTTCCTTGATGTTGTTTCATGGAATTCCTCCTGTTAGCCC		
LZ_23	ATGAGTGATTCTTCCCCAACG	*plu1879−1878* from *P. laumondii*	pLZ9
LZ_31	TTAGGTCTGGCTAACGACTTC		
LZ_32	AGGAAAATGAAGTCGTTAGCCAGACCTAACAATTAATCATCGGCTCGTATAATG	pCDF vector backbone	
LZ_26	TCAGGAAGGTGAACCGTTGGGGAAGAATCACTCATGGAATTCCTCCTGTTAGCCC		

Supplementary Figures

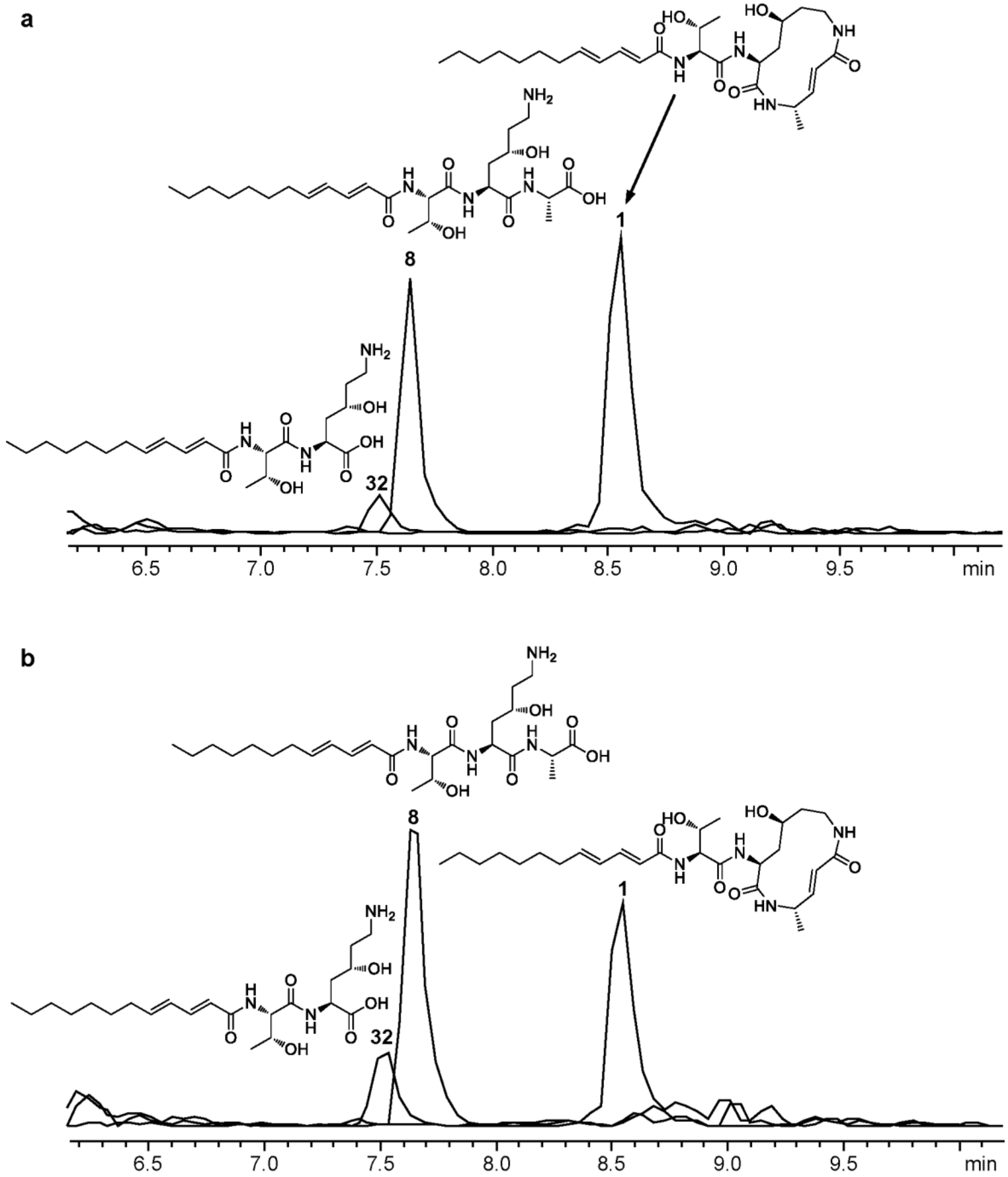

Figure S1. Production of GLNPs in (a) *E. coli plu1881−1877* and (b) *E. coli plu1881−1880* and *plu1879−1877*. Extracted ion chromatograms (EICs) are shown.

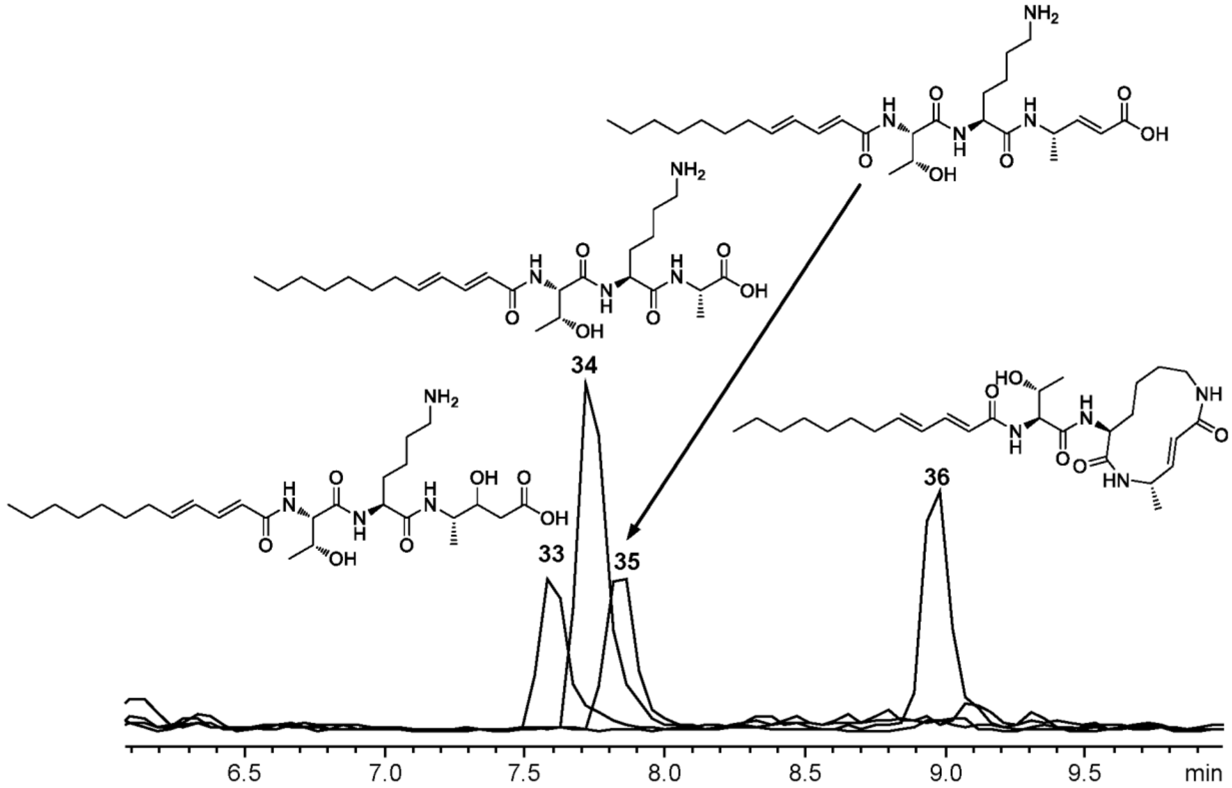

Figure S2. Production of GLNPs in *E. coli plu1880* and *plu1879−1877* (without *plu1881*). EICs are shown.

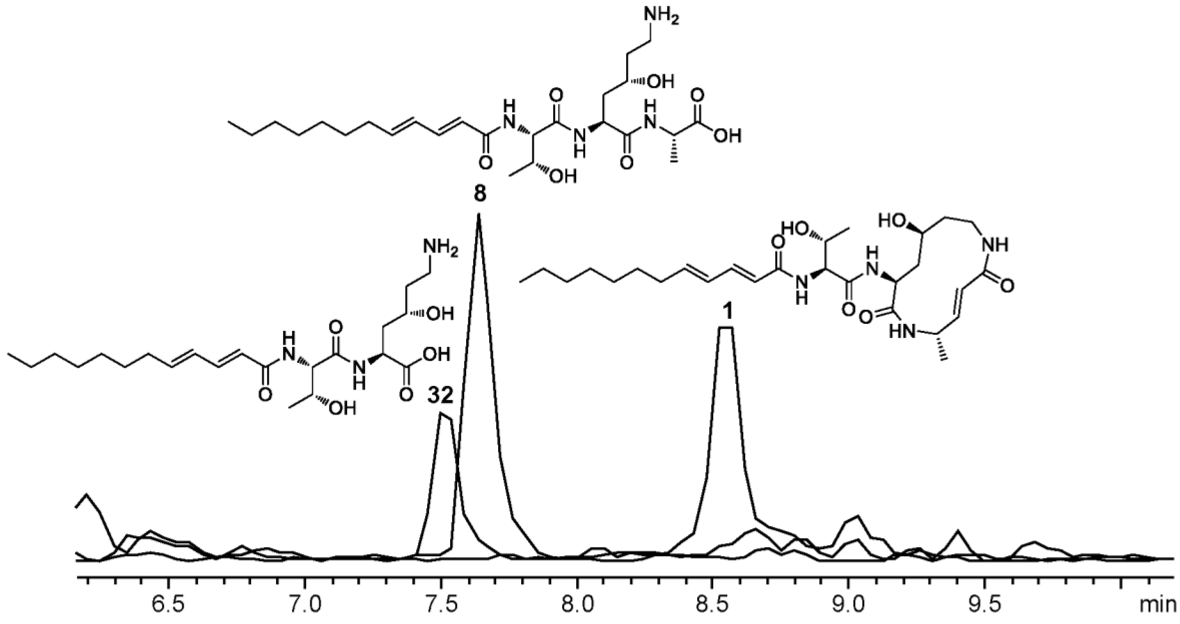

Figure S3. Production of GLNPs in *E. coli plu1881−1880* and *plu1878−1877* (without *plu1879*). EICs are shown.

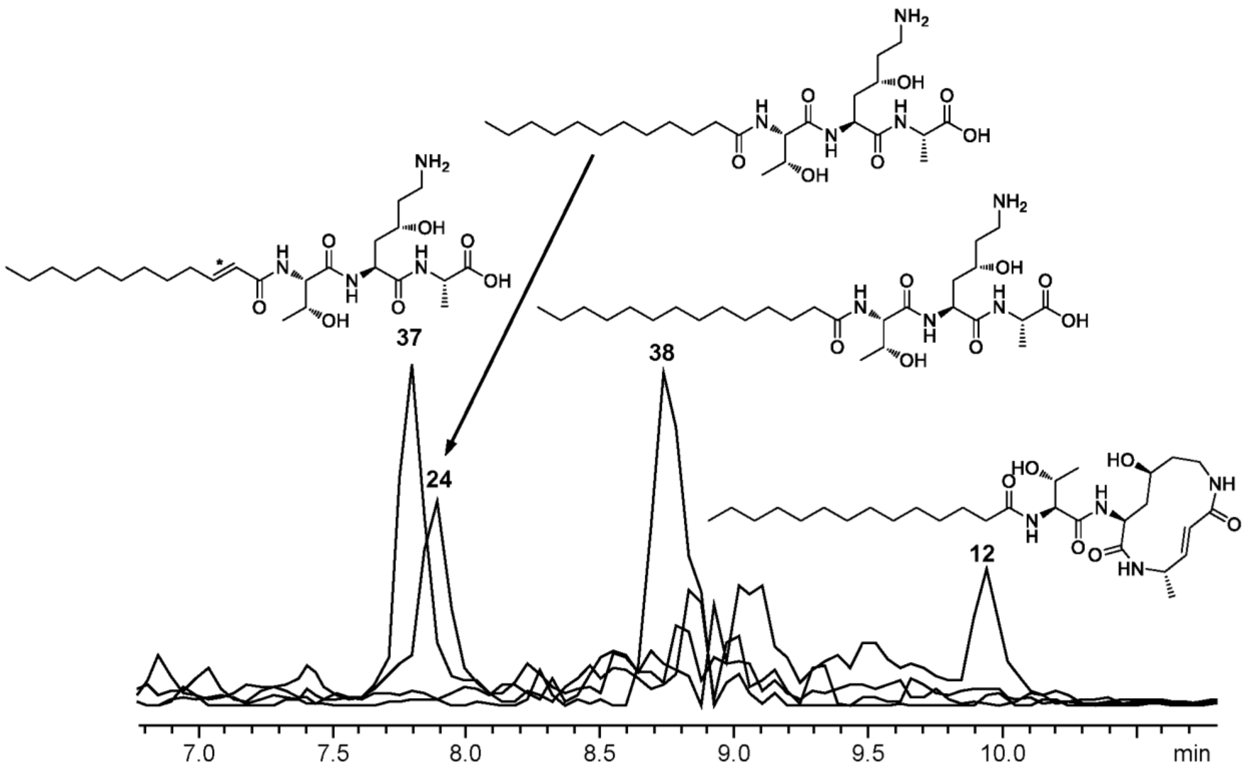

Figure S4. Production of GLNPs in *E. coli plu1881−1880* and *plu1879−1878* (without *plu1877*). EICs are shown. *the position of the double bond is not determined. Only ~1/8 of the amount of GLNPs were produced in this strain compared to strains carrying *plu1877*.

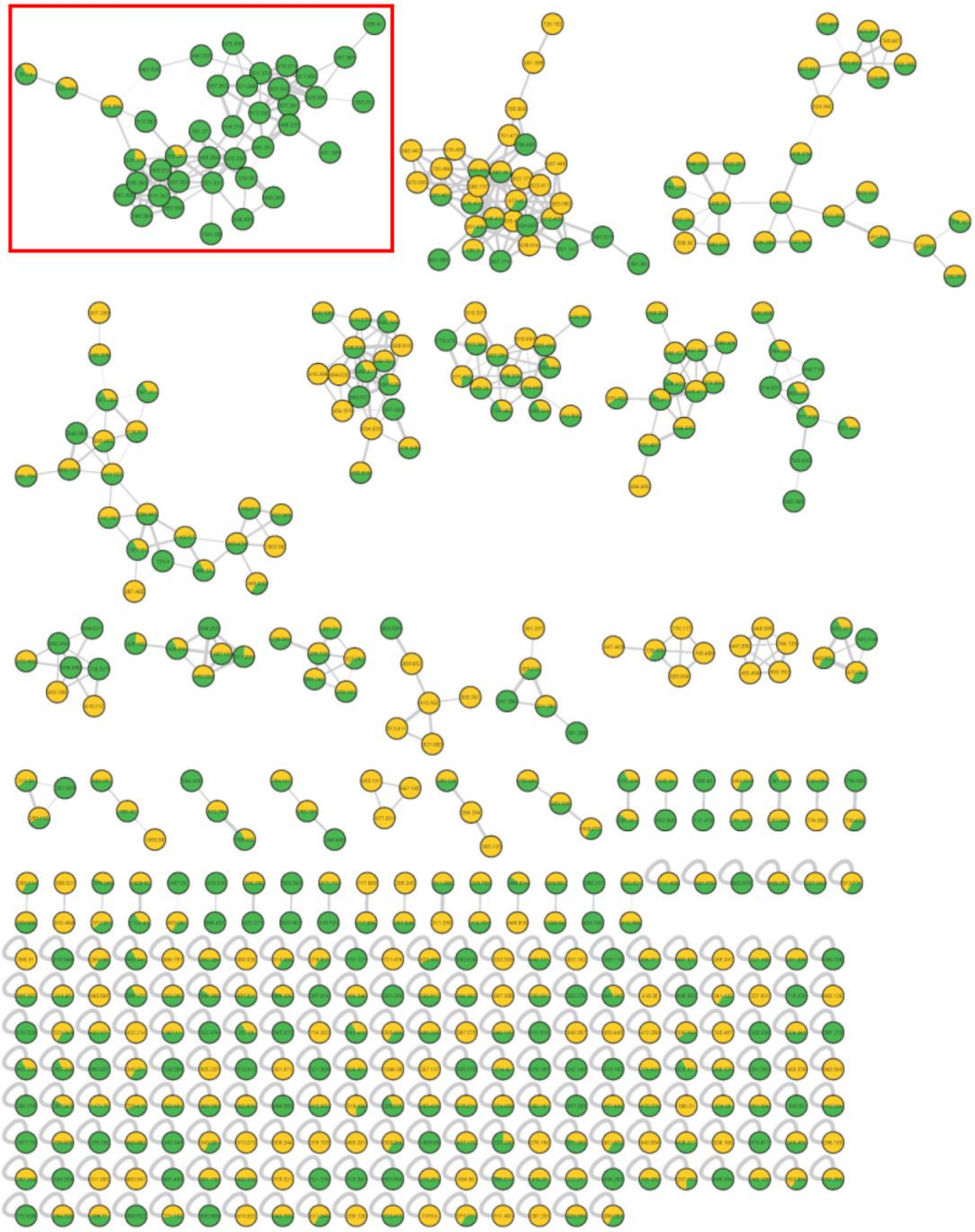

Figure S5. The overall network of molecular networking for MeOH extracts of *P. laumondii* wild type (yellow) and pCEP_gli mutant (green). GLNP subnetwork is shown in red frame.

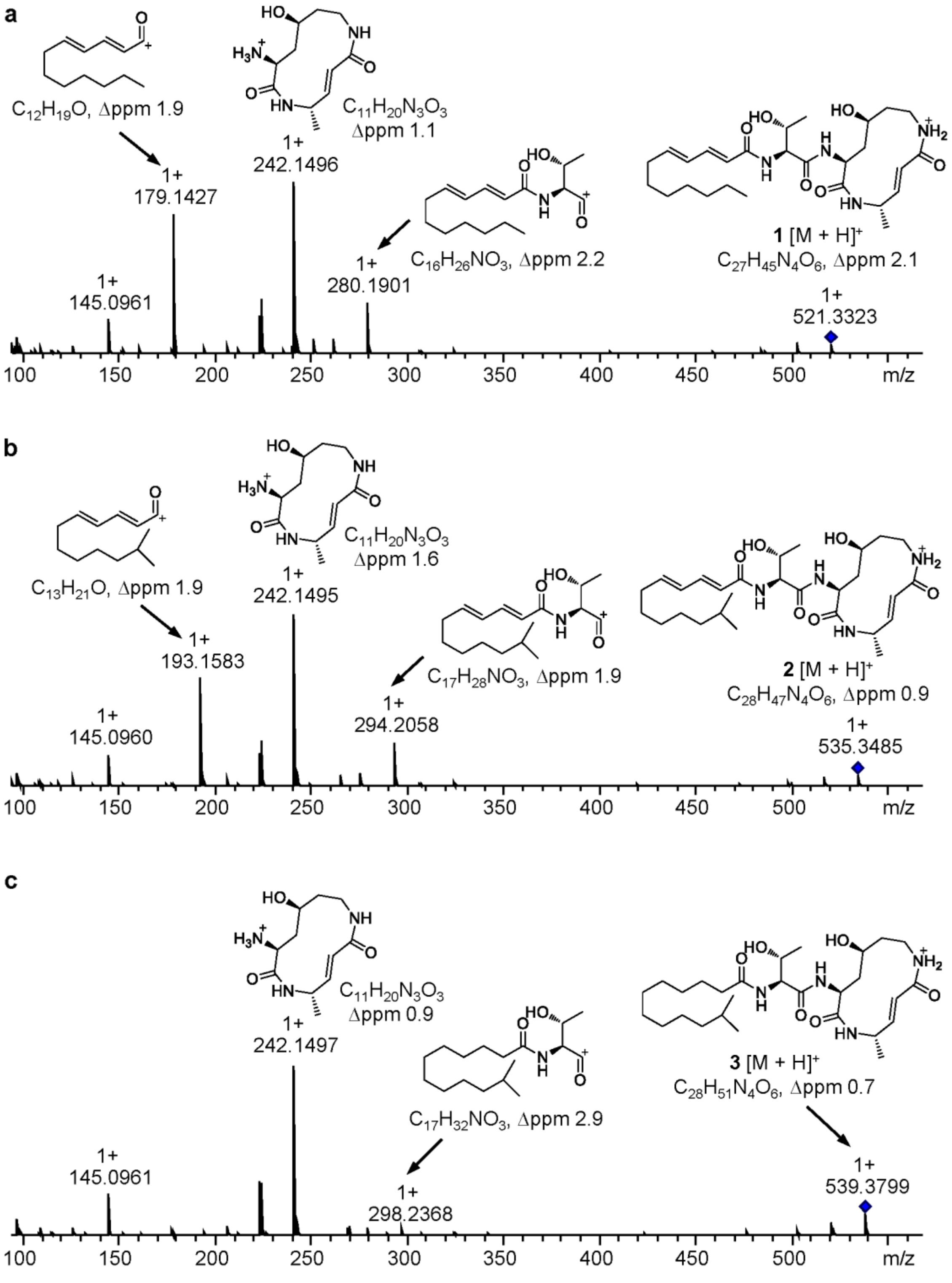
a
C12H19O, Δppm 1.9
1+
179.1427
C11H20N3O3
Δppm 1.1
1+
242.1496
C16H26NO3, Δppm 2.2
1+
280.1901
1+
145.0961
1 [M + H]+
C27H45N4O6, Δppm 2.1
1+
521.3323
b
C13H21O, Δppm 1.9
1+
193.1583
C11H20N3O3
Δppm 1.6
1+
242.1495
C17H28NO3, Δppm 1.9
1+
294.2058
1+
145.0960
2 [M + H]+
C28H47N4O6, Δppm 0.9
1+
535.3485
c
C11H20N3O3
Δppm 0.9
1+
242.1497
C17H32NO3, Δppm 2.9
1+
298.2368
1+
145.0961
3 [M + H]+
C28H51N4O6, Δppm 0.7
1+
539.3799
m/z

d

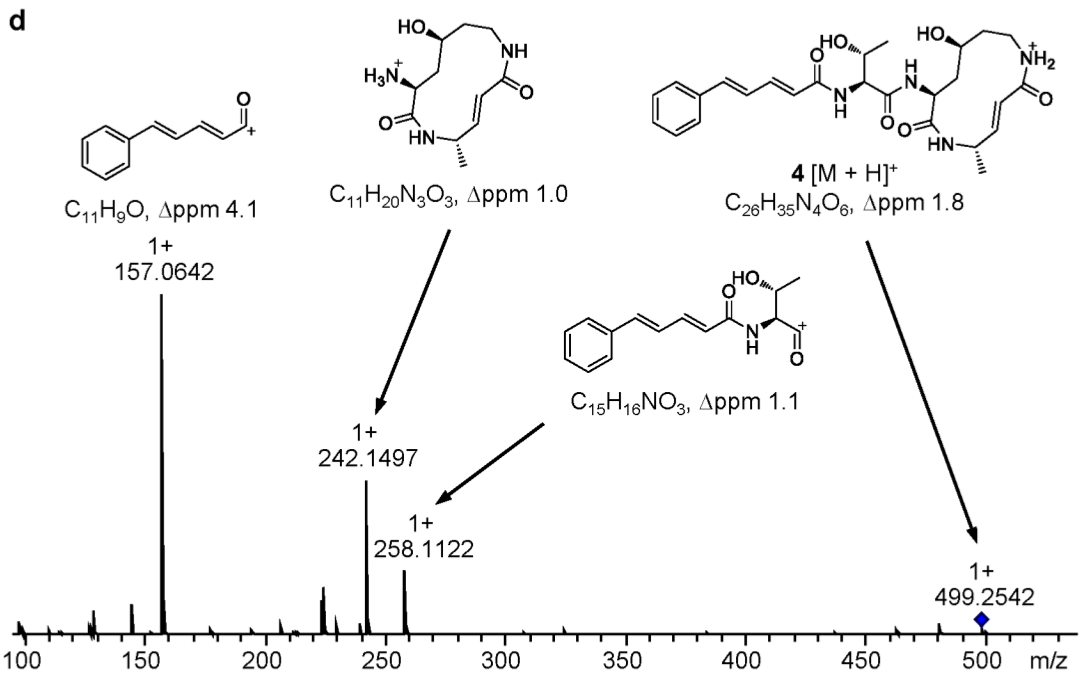

C11H9O, Δppm 4.1
1+
157.0642
C11H20N3O3, Δppm 1.0
4 [M + H]+
C26H35N4O6, Δppm 1.8
C15H16NO3, Δppm 1.1
1+
242.1497
1+
258.1122
1+
499.2542
100
150
200
250
300
350
400
450
500
m/z

e

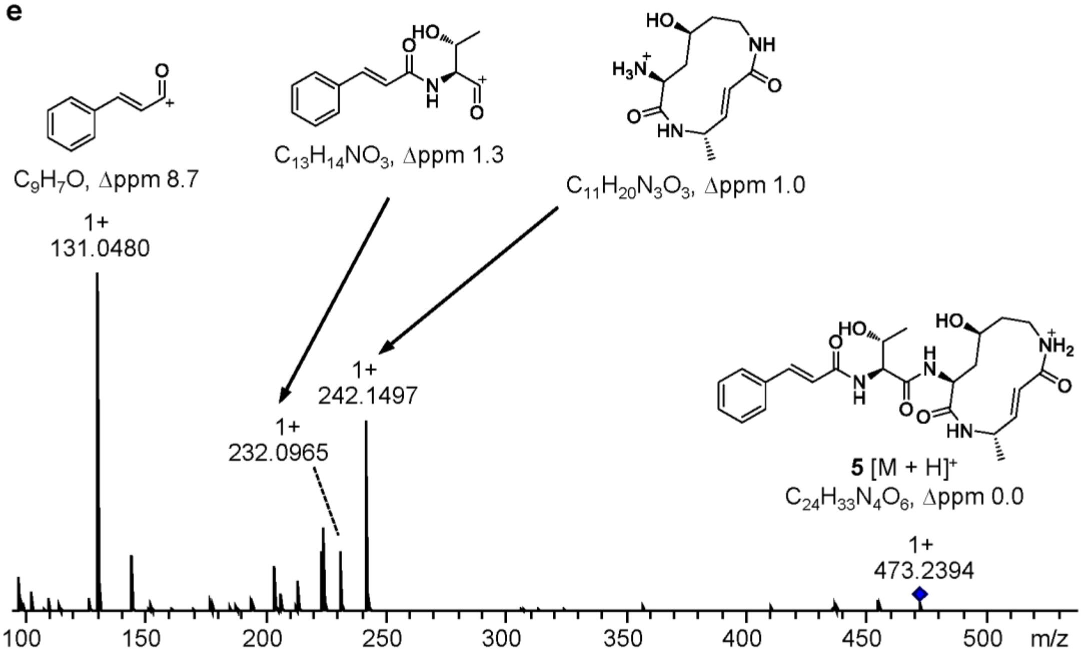

C9H7O, Δppm 8.7
C13H14NO3, Δppm 1.3
C11H20N3O3, Δppm 1.0
1+
131.0480
1+
242.1497
1+
232.0965
5 [M + H]+
C24H33N4O6, Δppm 0.0
1+
473.2394
100
150
200
250
300
350
400
450
500
m/z

f

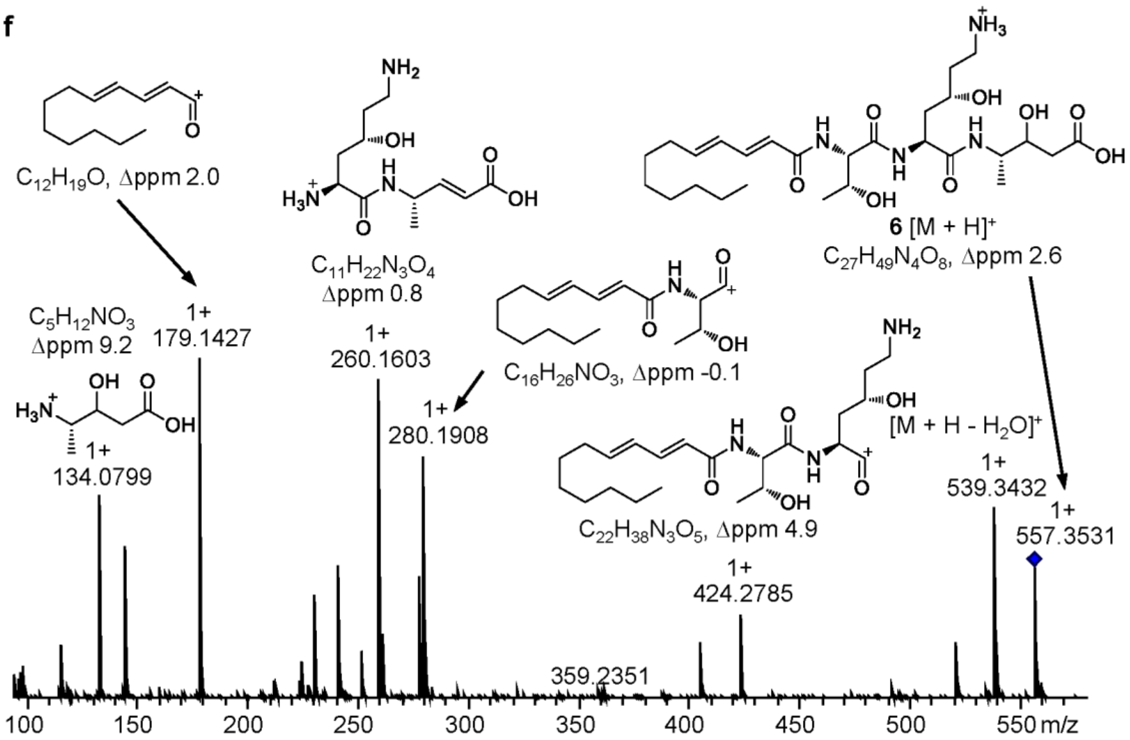

$C_{12}H_{19}O$, Δppm 2.0
$C_{11}H_{22}N_3O_4$
Δppm 0.8
6 [M + H]+
$C_{27}H_{49}N_4O_8$, Δppm 2.6
$C_5H_{12}NO_3$
Δppm 9.2
1+
179.1427
1+
260.1603
$C_{16}H_{26}NO_3$, Δppm -0.1
1+
280.1908
1+
134.0799
[M + H - H2O]+
1+
539.3432
1+
557.3531
$C_{22}H_{38}N_3O_5$, Δppm 4.9
1+
424.2785
359.2351
m/z

g

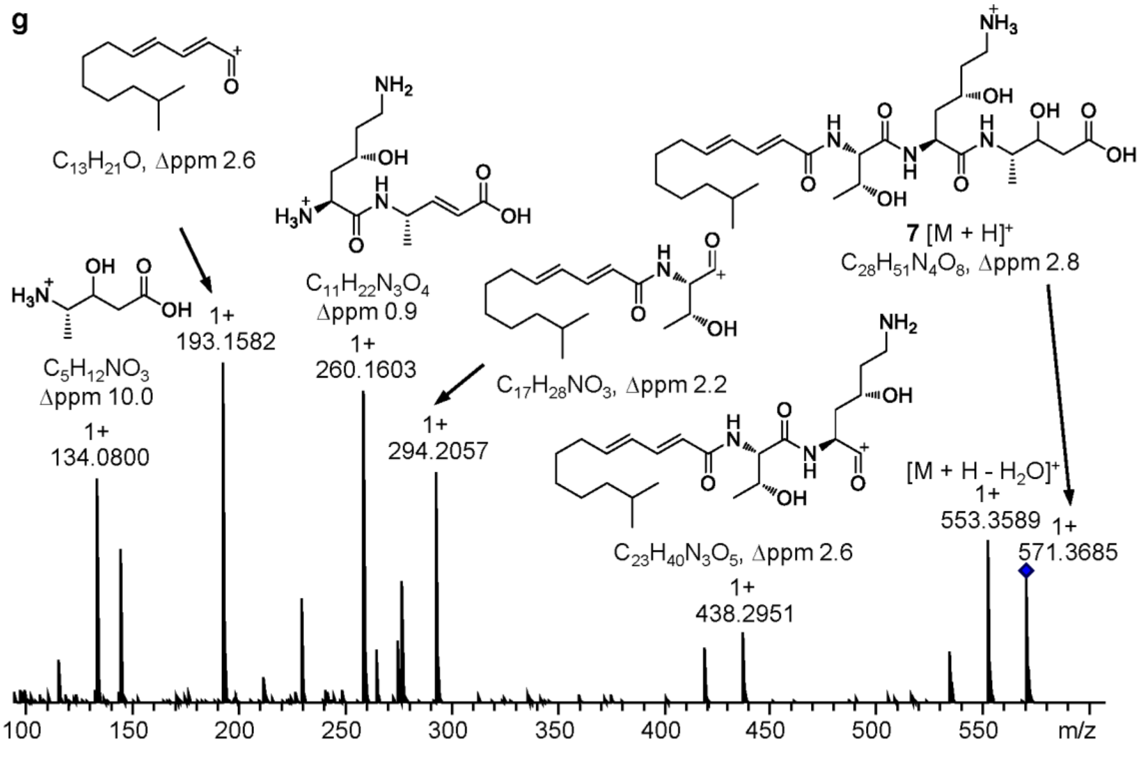

$C_{13}H_{21}O$, Δppm 2.6
$C_{11}H_{22}N_3O_4$
Δppm 0.9
7 [M + H]+
$C_{28}H_{51}N_4O_8$, Δppm 2.8
1+
193.1582
$C_5H_{12}NO_3$
Δppm 10.0
1+
134.0800
1+
260.1603
$C_{17}H_{28}NO_3$, Δppm 2.2
1+
294.2057
[M + H - H2O]+
1+
553.3589
1+
571.3685
$C_{23}H_{40}N_3O_5$, Δppm 2.6
1+
438.2951
m/z

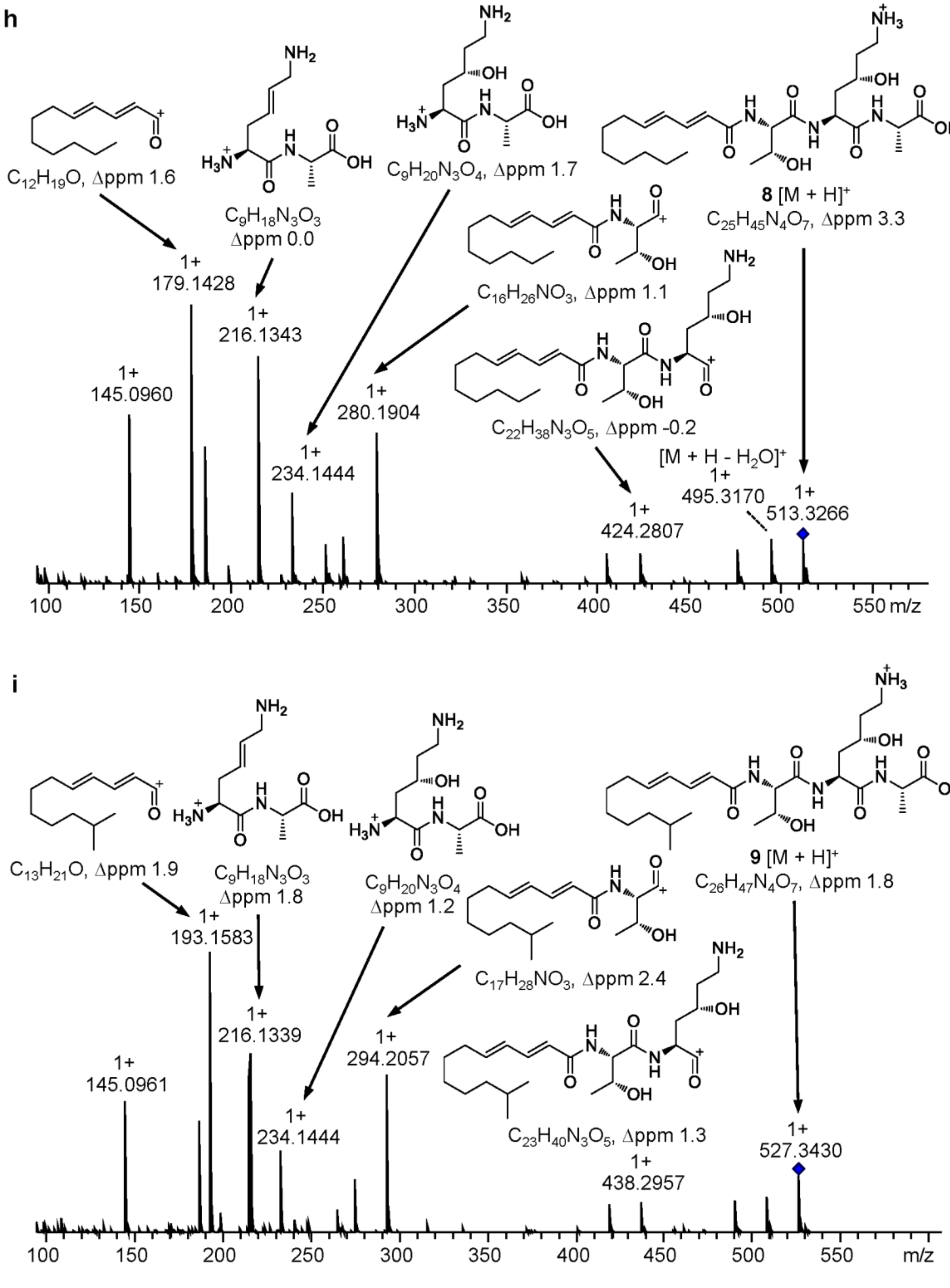

Figure S6. Structure confirmation of **1**−**9** by their MS/MS fragmentation patterns. MS/MS spectra and proposed fragment structures are shown for (a−i) **1**−**9**.

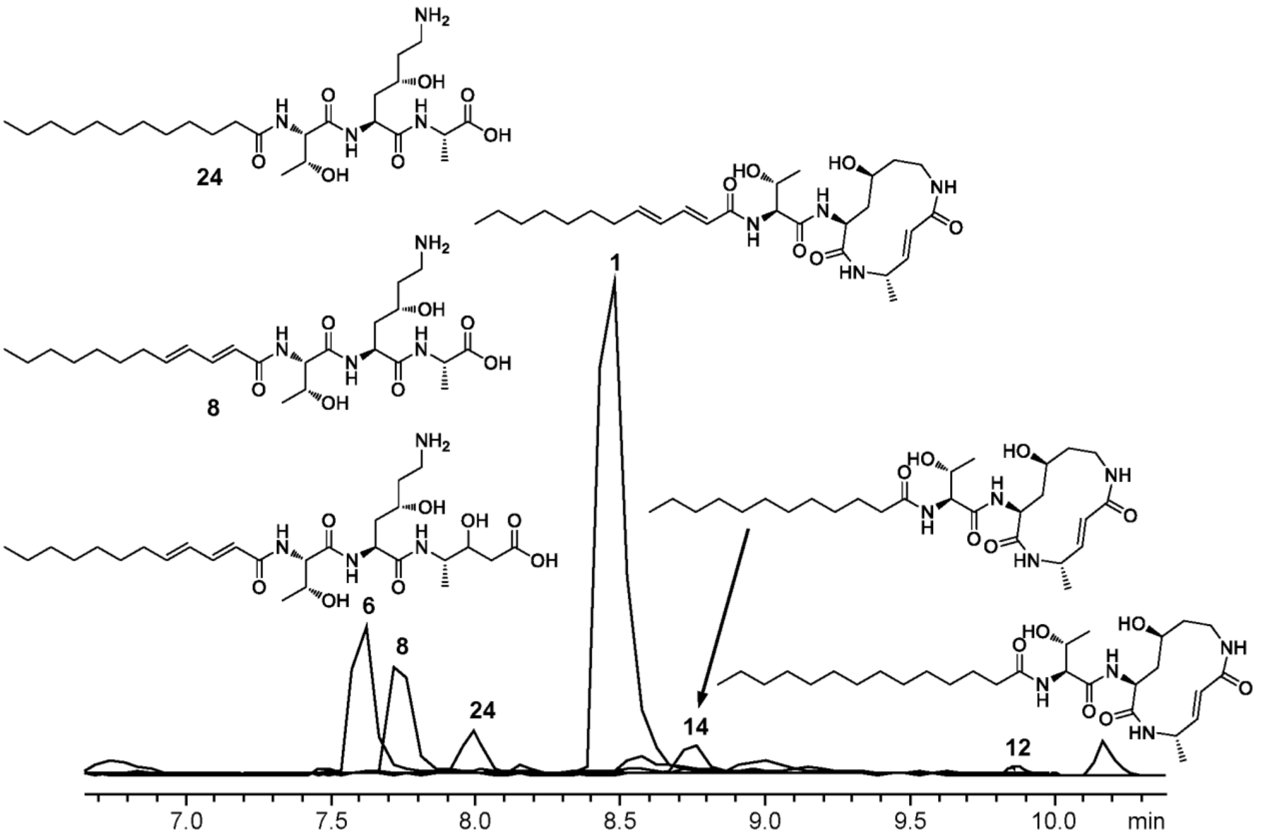

Figure S7. Production of straight-chain fatty acid moiety containing GLNPs in *P. laumondii* Δ*bkdABC* pCEP_gli mutant. EICs are shown.

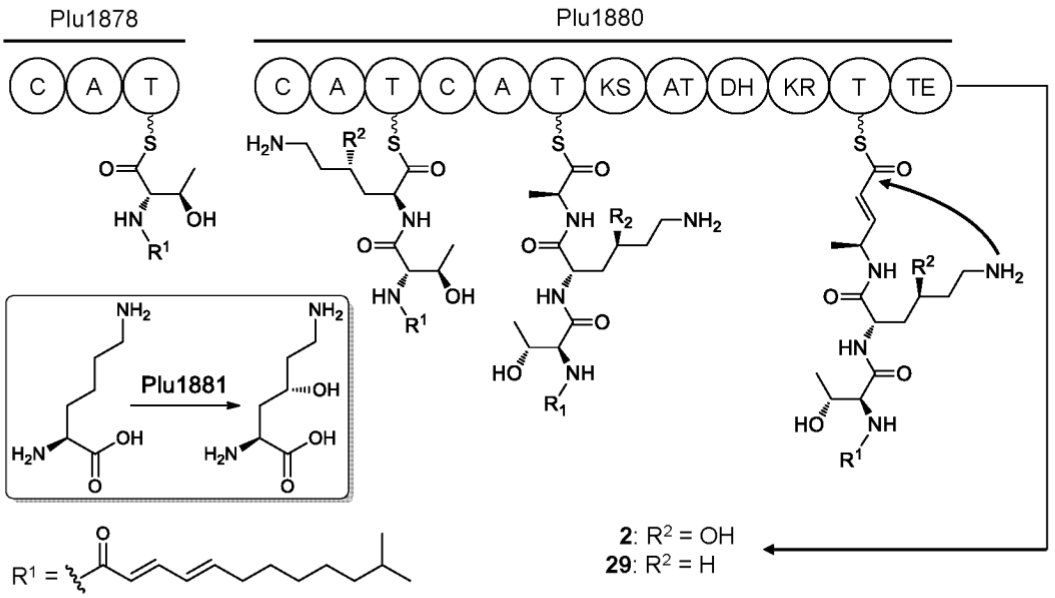

Figure S8. Proposed biosynthesis for selected GLNP **2** from subclass I and GLNP **29** from subclass IV. As the starter module, the non-ribosomal peptide synthetase (NRPS) Plu1878 activates *N*-acylated L-threonine with a coenzyme A (CoA)-activated fatty acid. The first NRPS module of Plu1880 activates L-lysine or (*S*)-4-hydroxy L-lysine, which is oxidized from L-lysine by Plu1881. The second NRPS module in Plu1880 activates L-alanine, which is further modified to 4-amino-2-pentenoic acid by polyketide synthetase (PKS) module of Plu1880. The macrolactam ring formation and the release of final product are catalyzed by the thioesterase (TE) domain. Domains: C: condensation, A: adenylation, T: thiolation, KS: ketosynthase, AT: acyltransferase, DH: dehydratase, KR: ketoreductase.

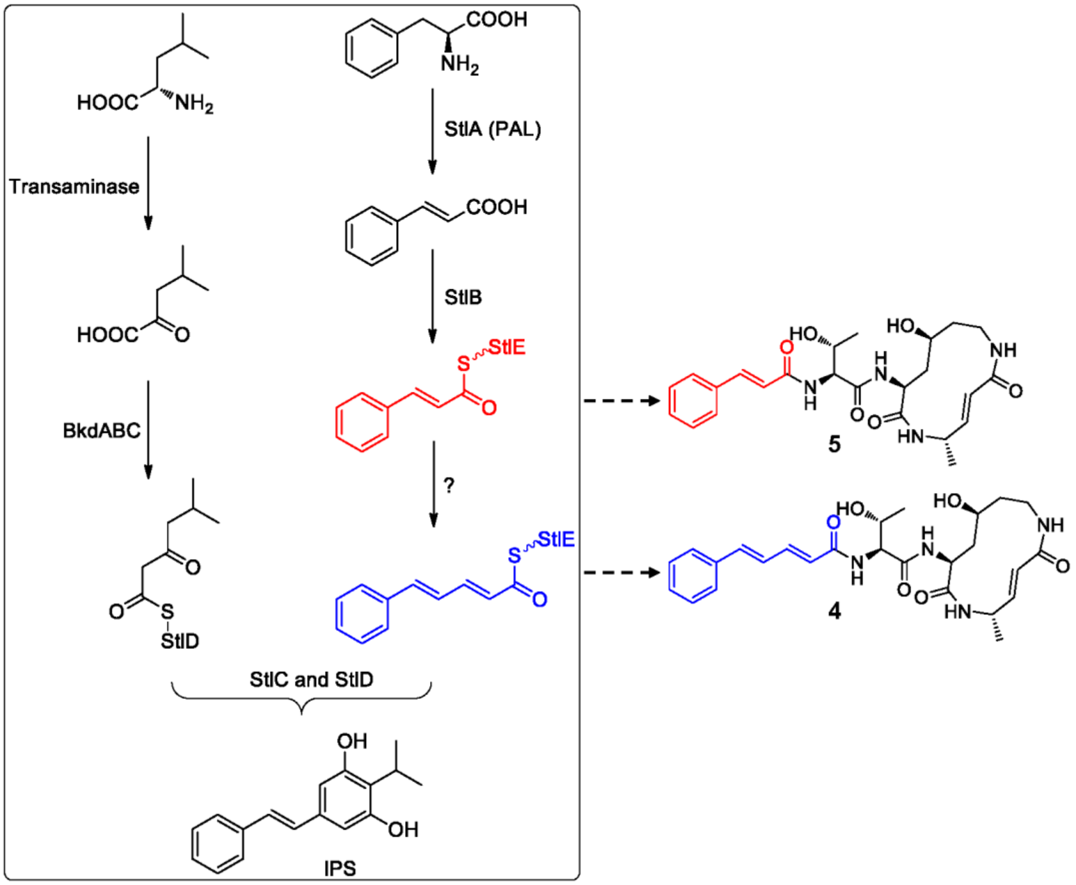

Figure S9. Possible biosynthesis for cinnamalacetic acid moiety of selected GLNP **4** and cinnamic acid moiety of selected GLNP **5** from subclass II. The biosynthesis for isopropylstilbene (IPS) in *P. laumondii* is shown in left frame.[23,24] StlA (PAL): phenylalanine ammonium lyase, StlB: CoA ligase, StlC: cyclase, StlD: cinnamoyl-CoA condensing ketosynthase, Bkd: branched-chain keto acid dehydrogenase (BkdA, BkdB), BkdC: isovaleryl-CoA condensing ketosynthase.

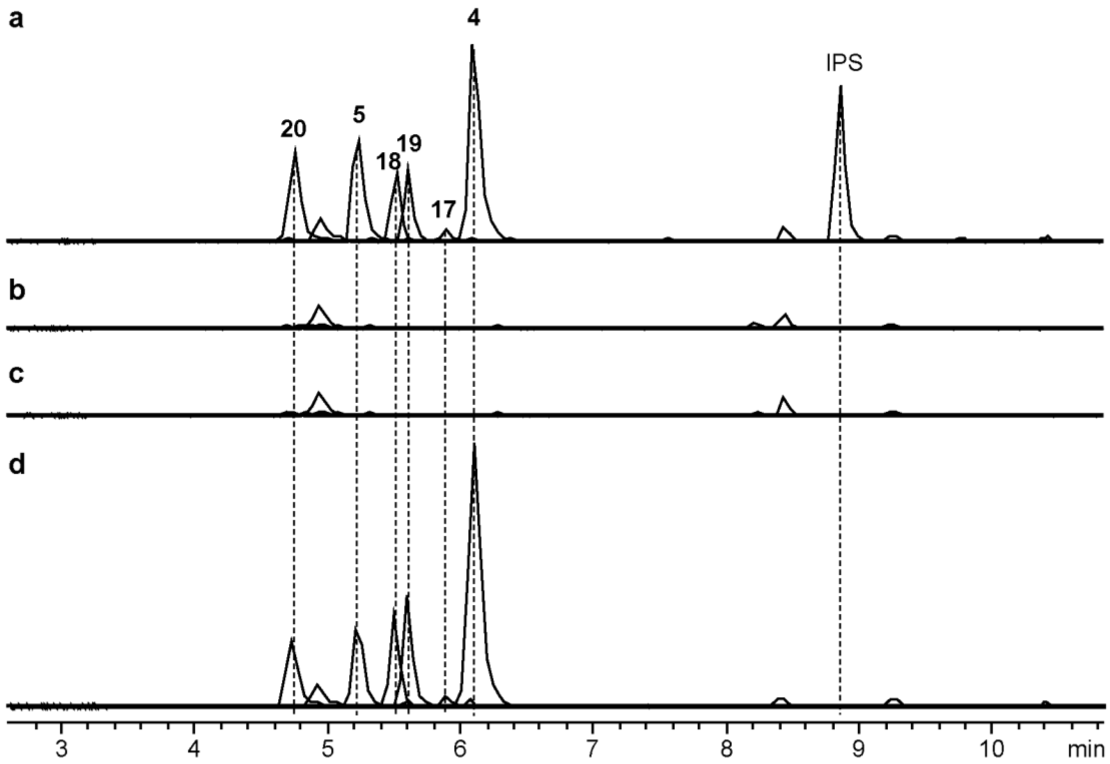

Figure S10. Production of IPS and cinnamic acid and cinnamalacetic acid containing GLNPs in *P. laumondii* (a) pCEP-gli, (b) Δ*stlA* pCEP-gli, (c) Δ*stlB* pCEP-gli, and (d) Δ*stlCDE* pCEP_gli mutants.

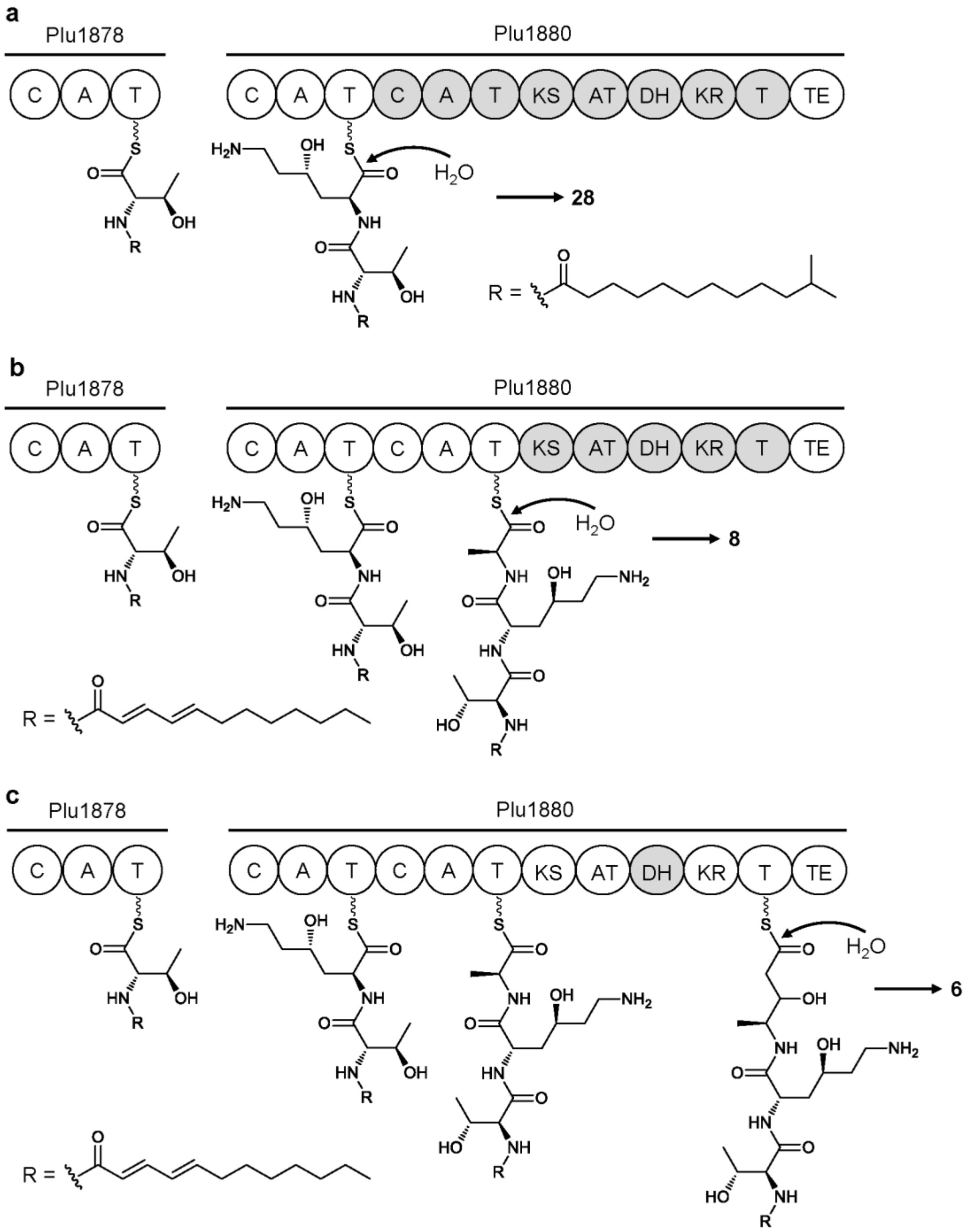

Figure S11. Proposed biosynthesis for selected GLNP (a) **28**, (b) **8** and (c) **6** from subclass III. The non-functional domains are shown in grey.

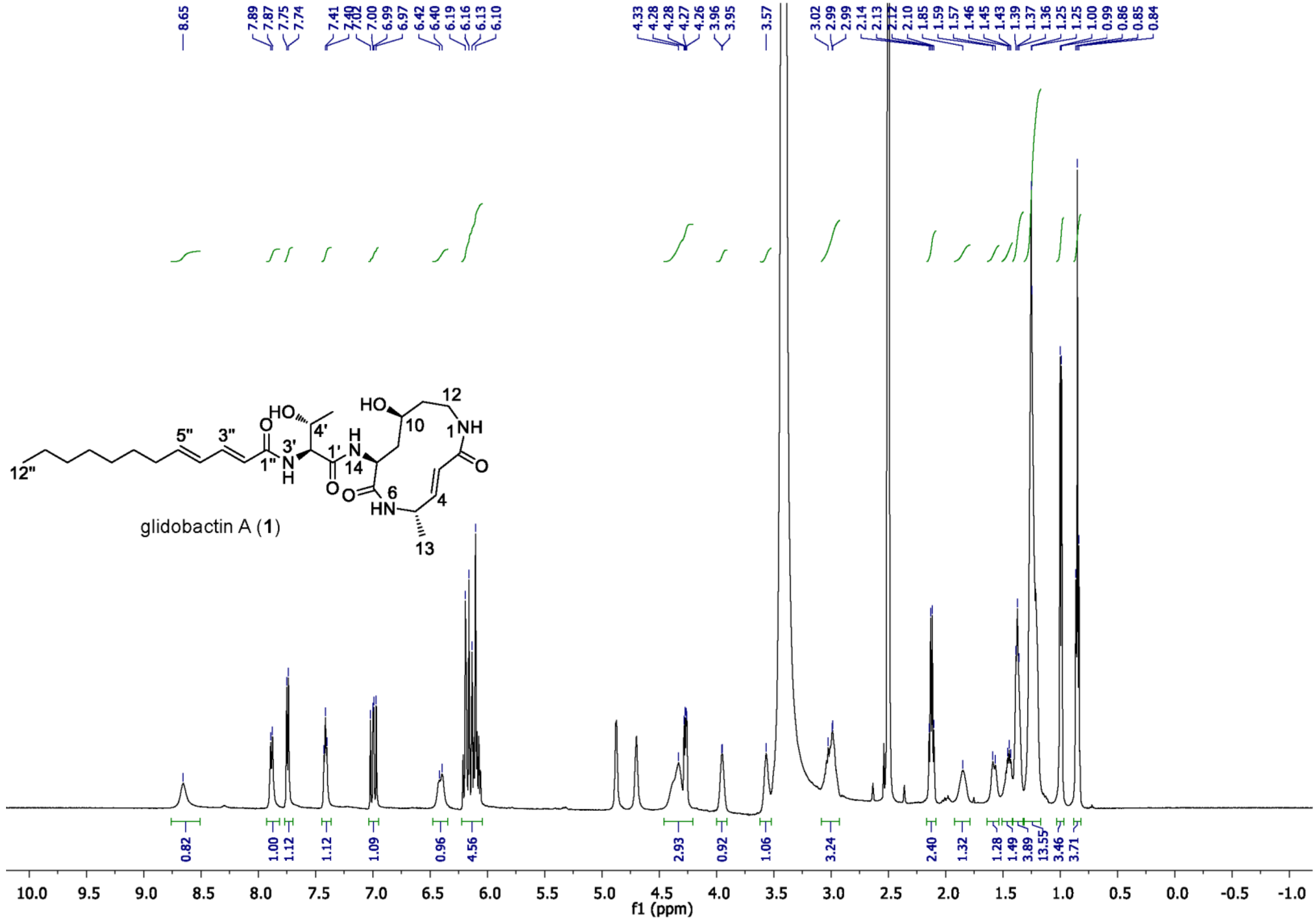

Figure S12. ^{1}H NMR (500 MHz, DMSO-d_6) spectrum of **1**.

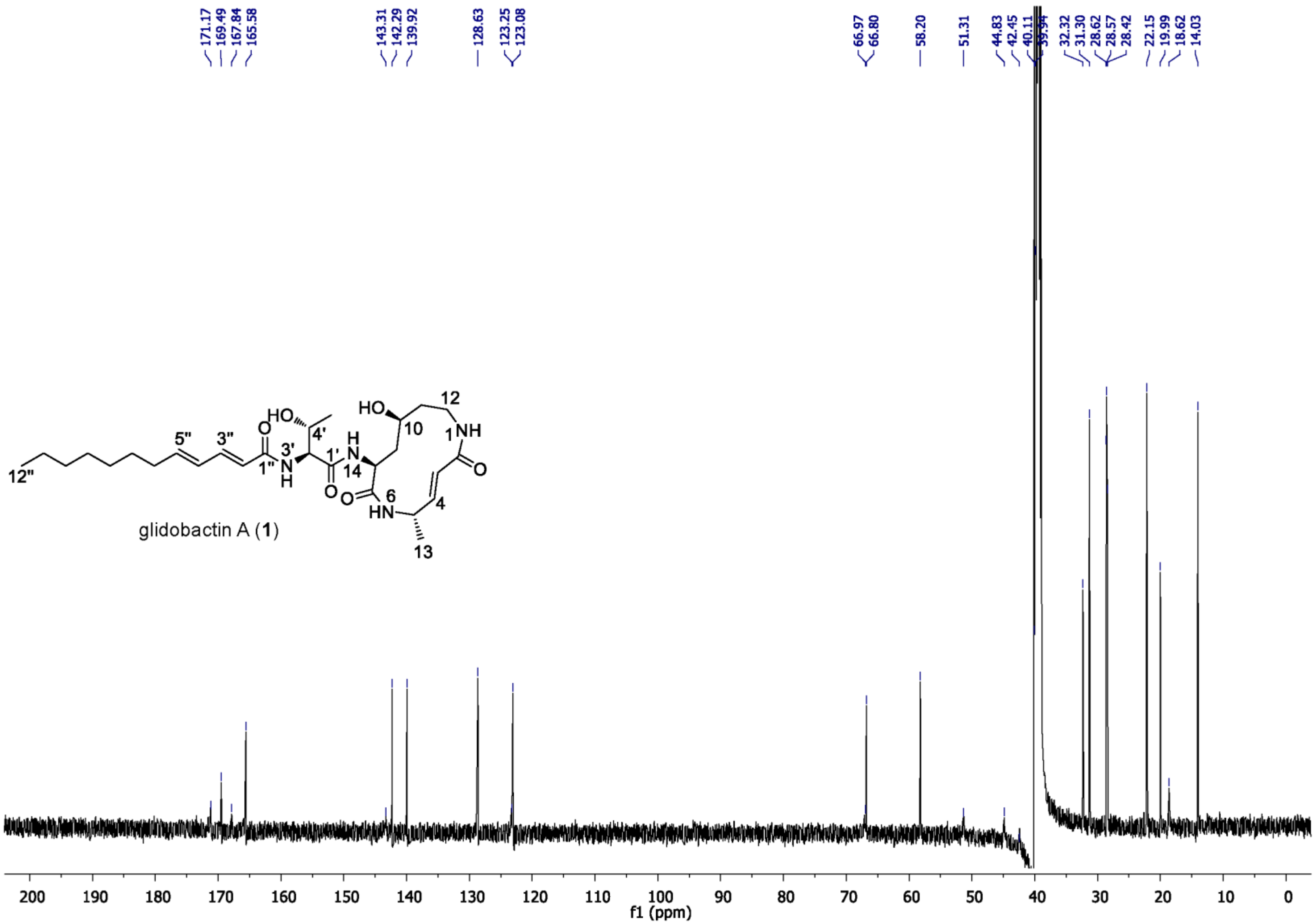

Figure S13. ^{13}C NMR (500 MHz, DMSO-d_6) spectrum of **1**.

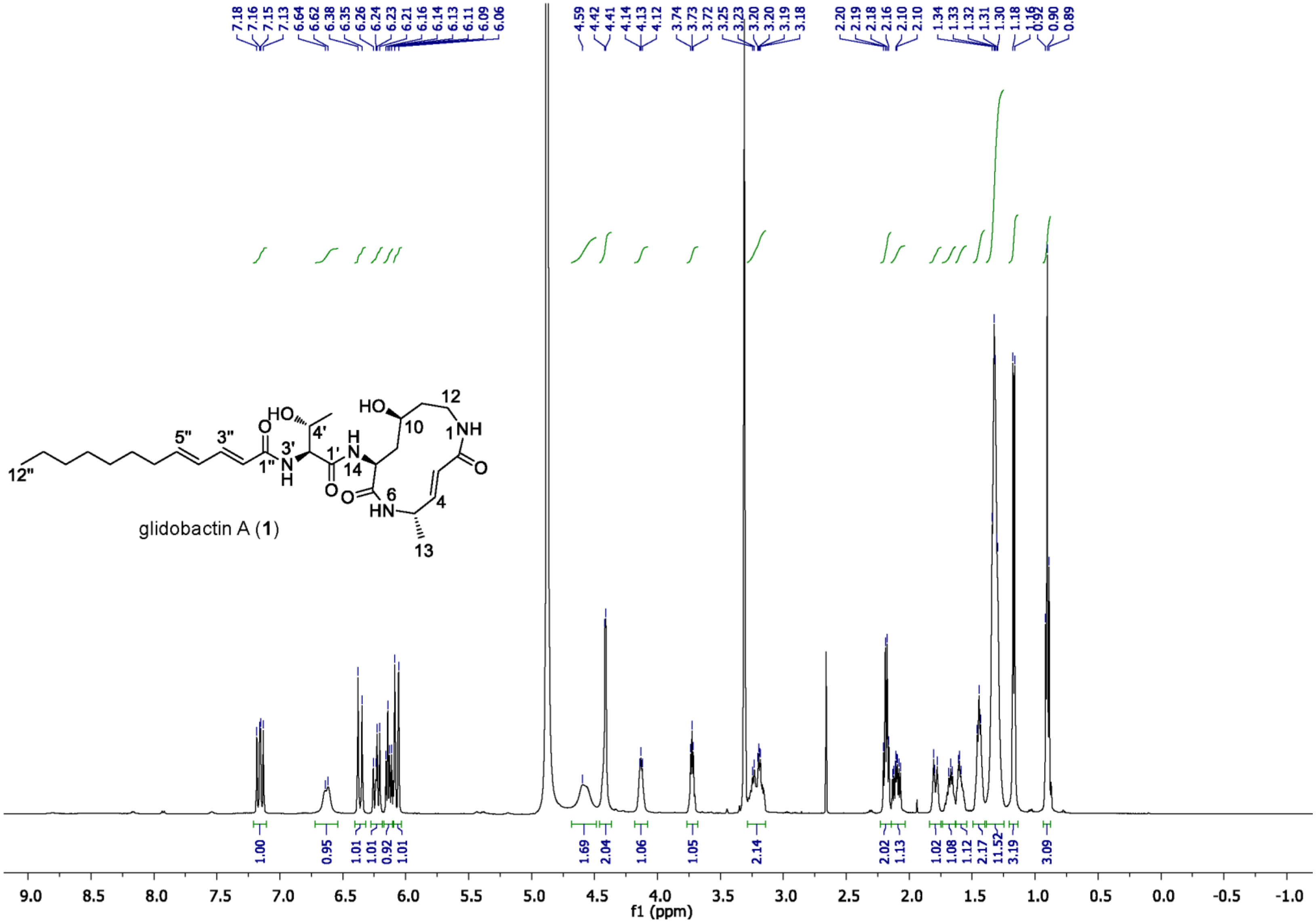

Figure S14. ^{1}H NMR (500 MHz, methanol-d$_4$) spectrum of **1**.

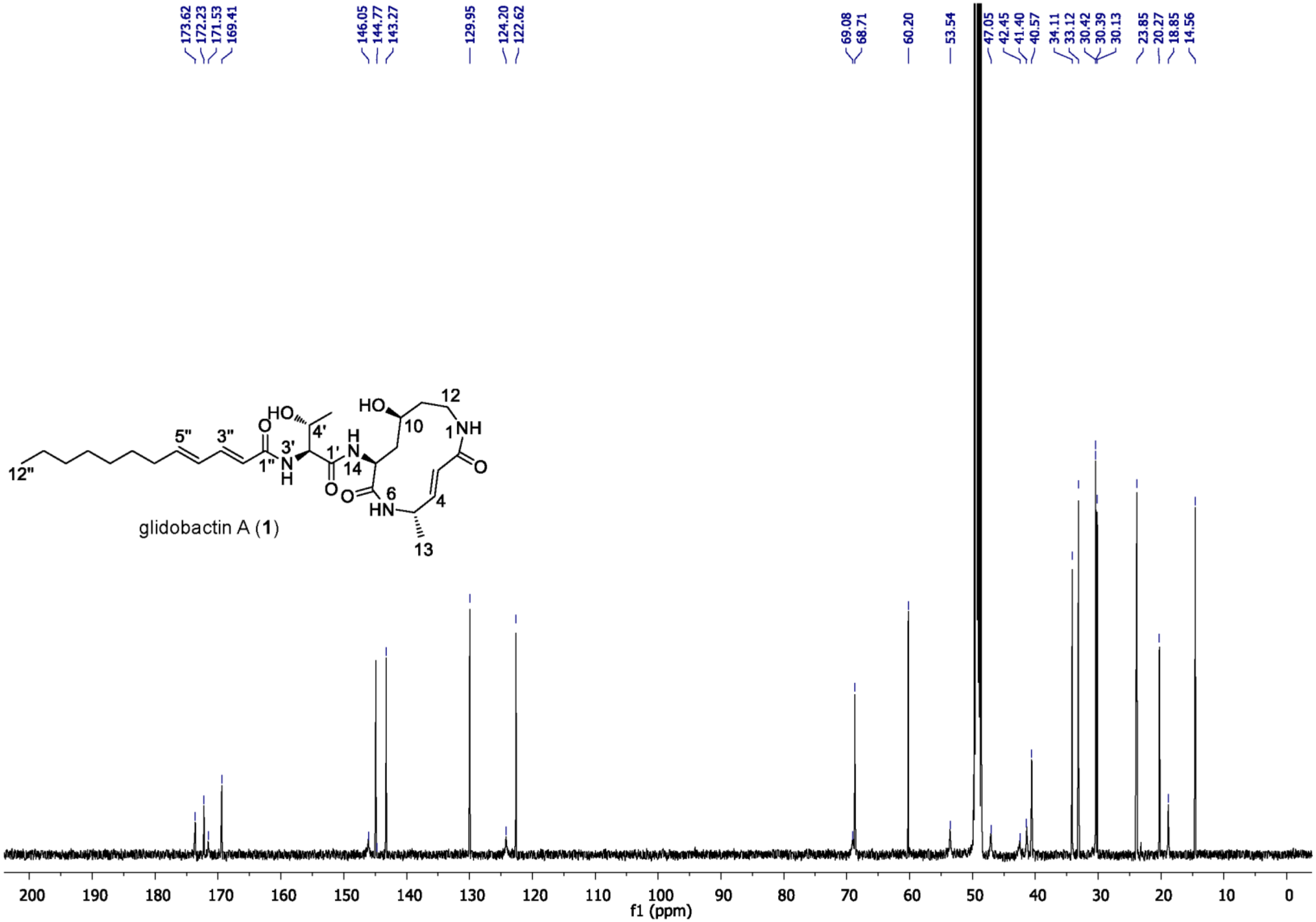

Figure S15. ^{13}C NMR (125 MHz, methanol-d_4) spectrum of **1**.

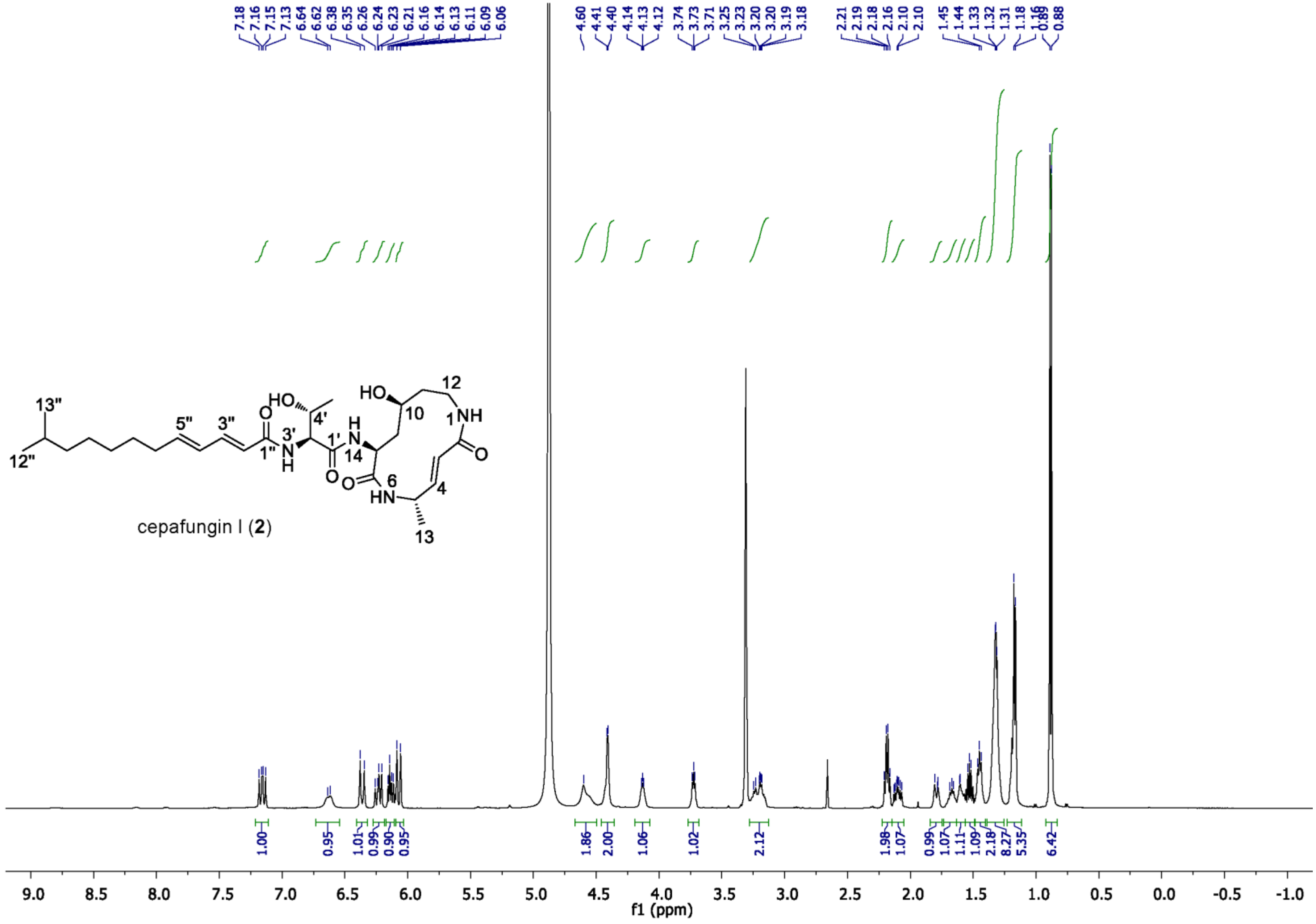

Figure S16. ^{1}H NMR (500 MHz, methanol-d_4) spectrum of **2**.

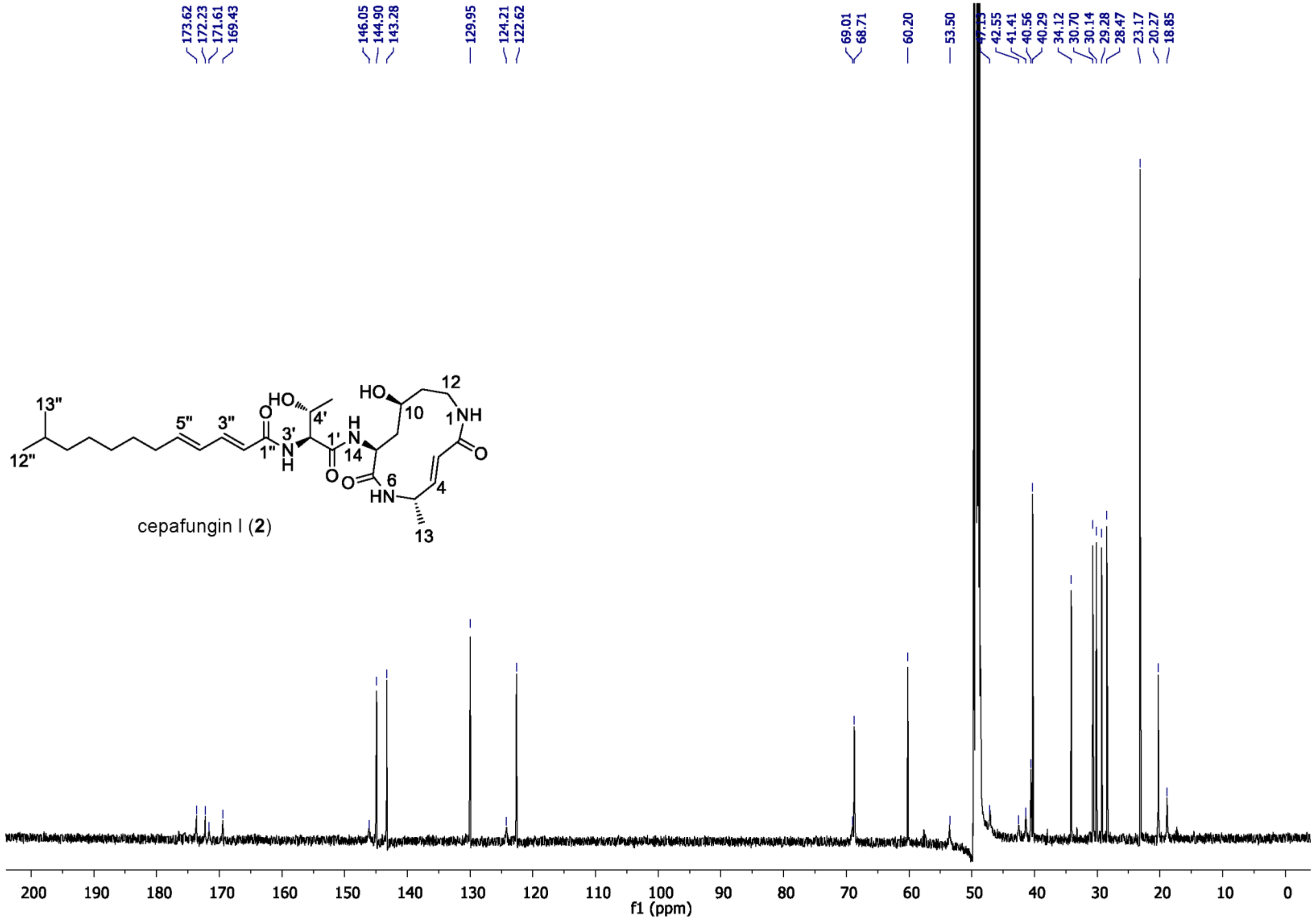

Figure S17. ^{13}C NMR (125 MHz, methanol-d_4) spectrum of **2**.

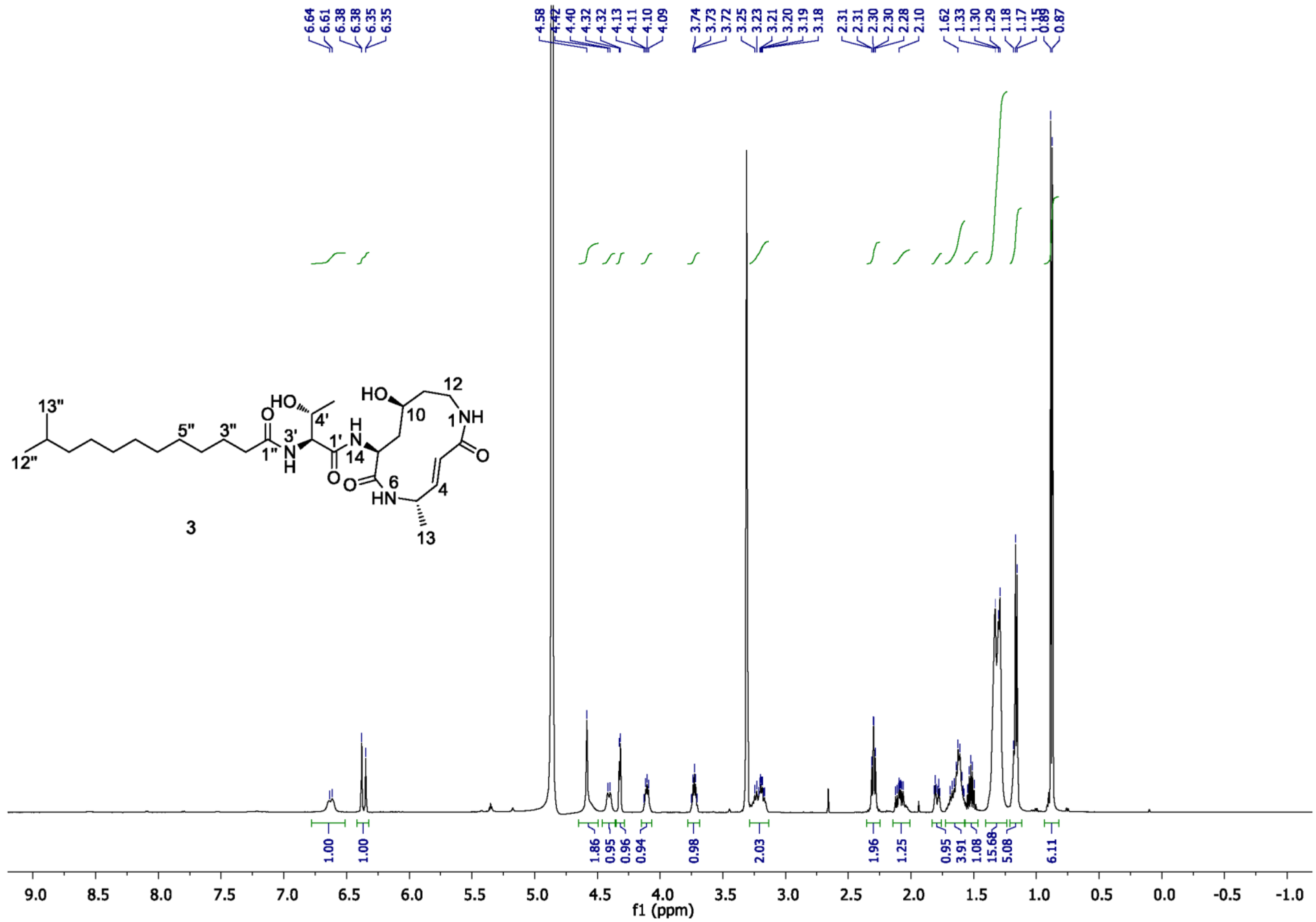

Figure S18. ^{1}H NMR (500 MHz, methanol-d_4) spectrum of **3**.

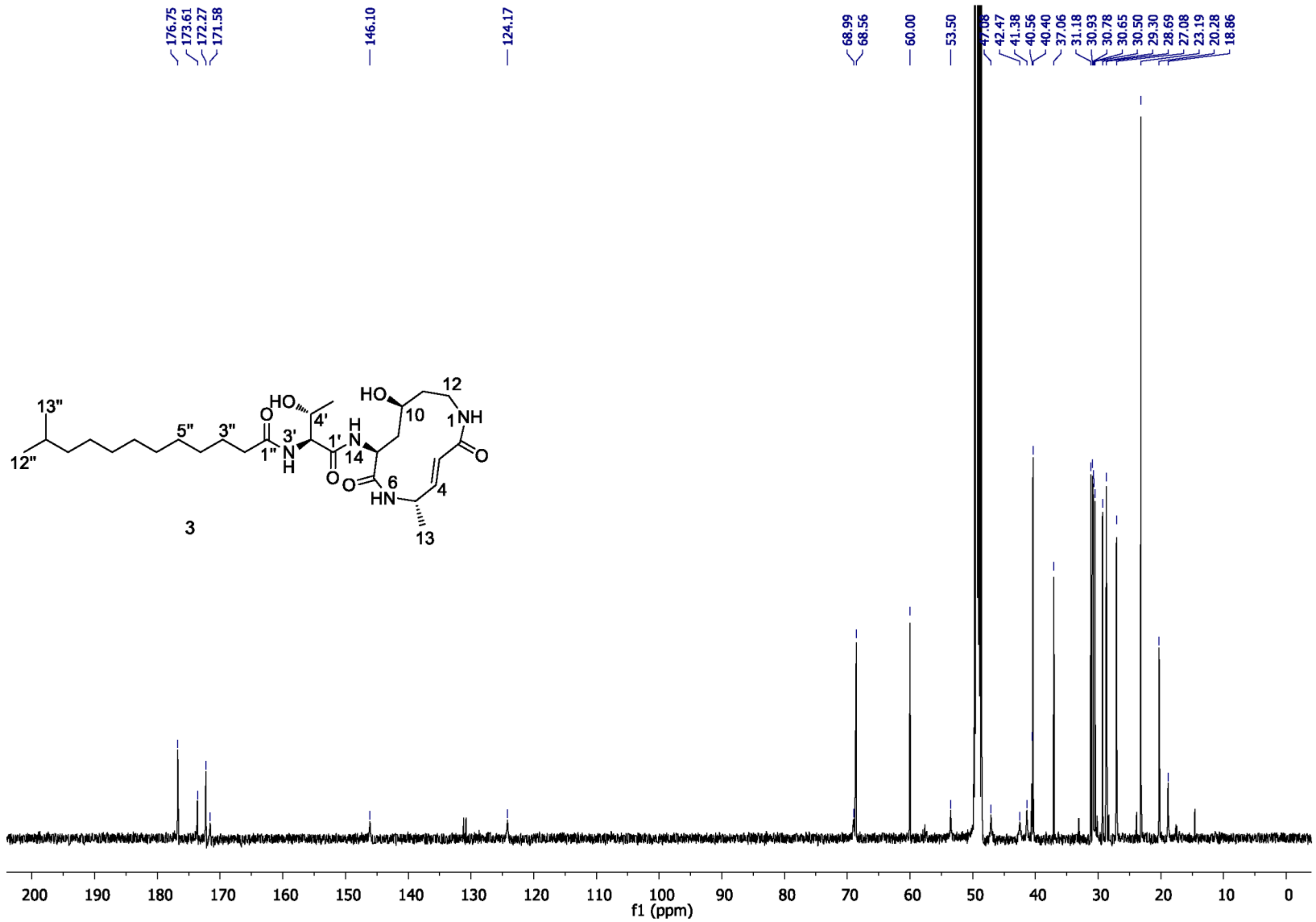

Figure S19. ^{13}C NMR (125 MHz, methanol-d_4) spectrum of **3**.

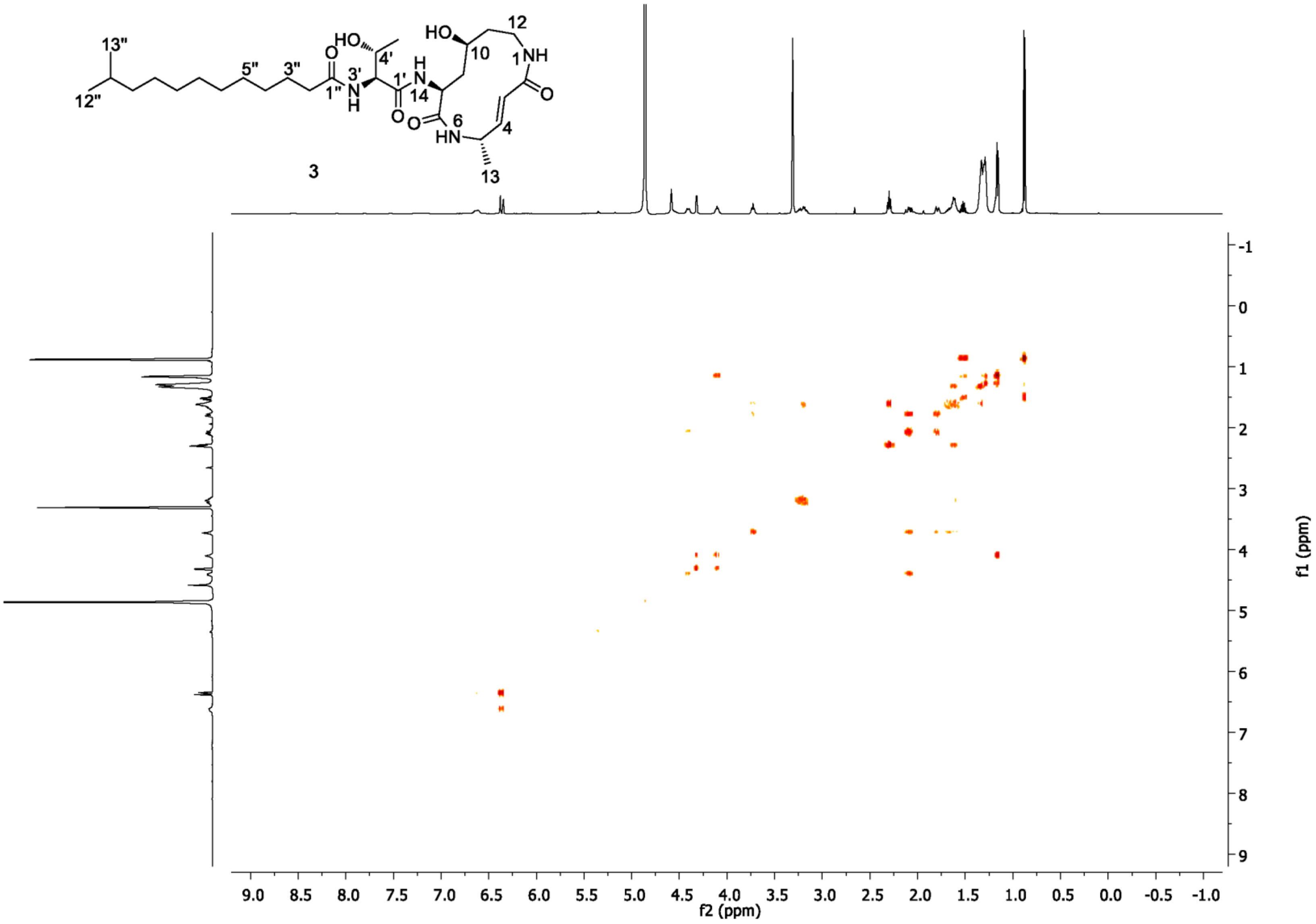

Figure S20. COSY (methanol-d_4) spectrum of **3**.

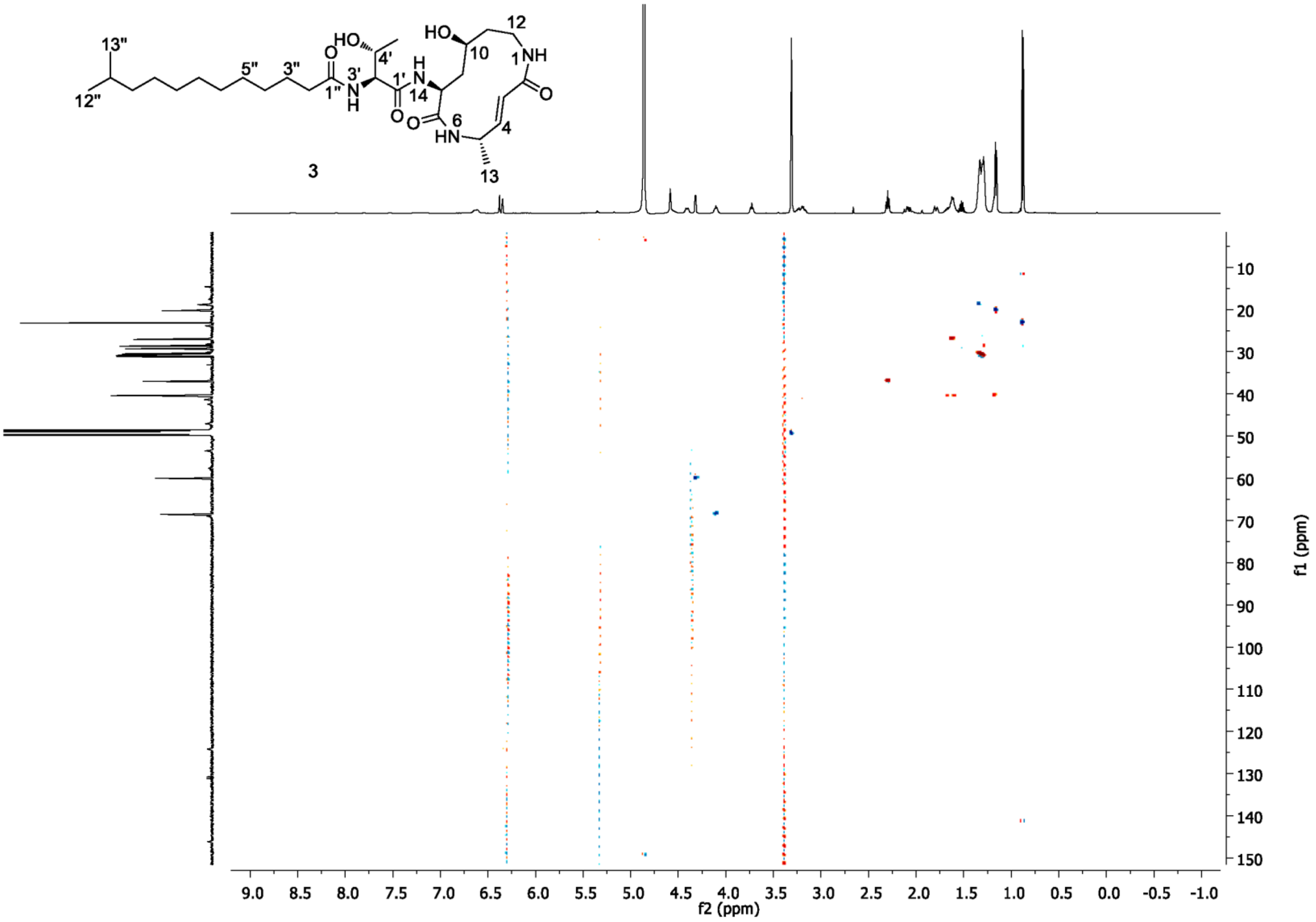

Figure S21. HSQC (methanol-d_4) spectrum of **3**.

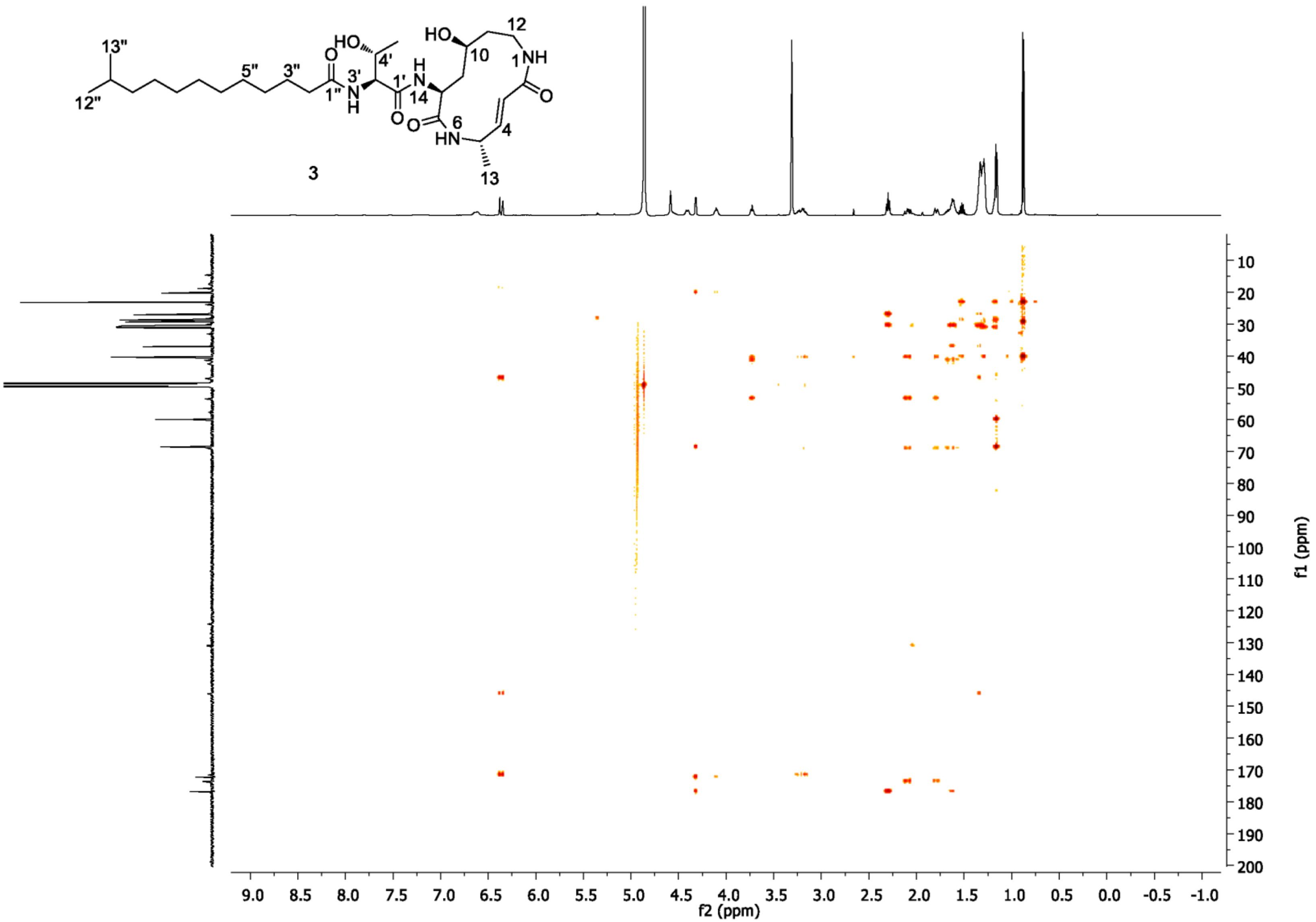

Figure S22. HMBC (methanol-d4) spectrum of **3**.

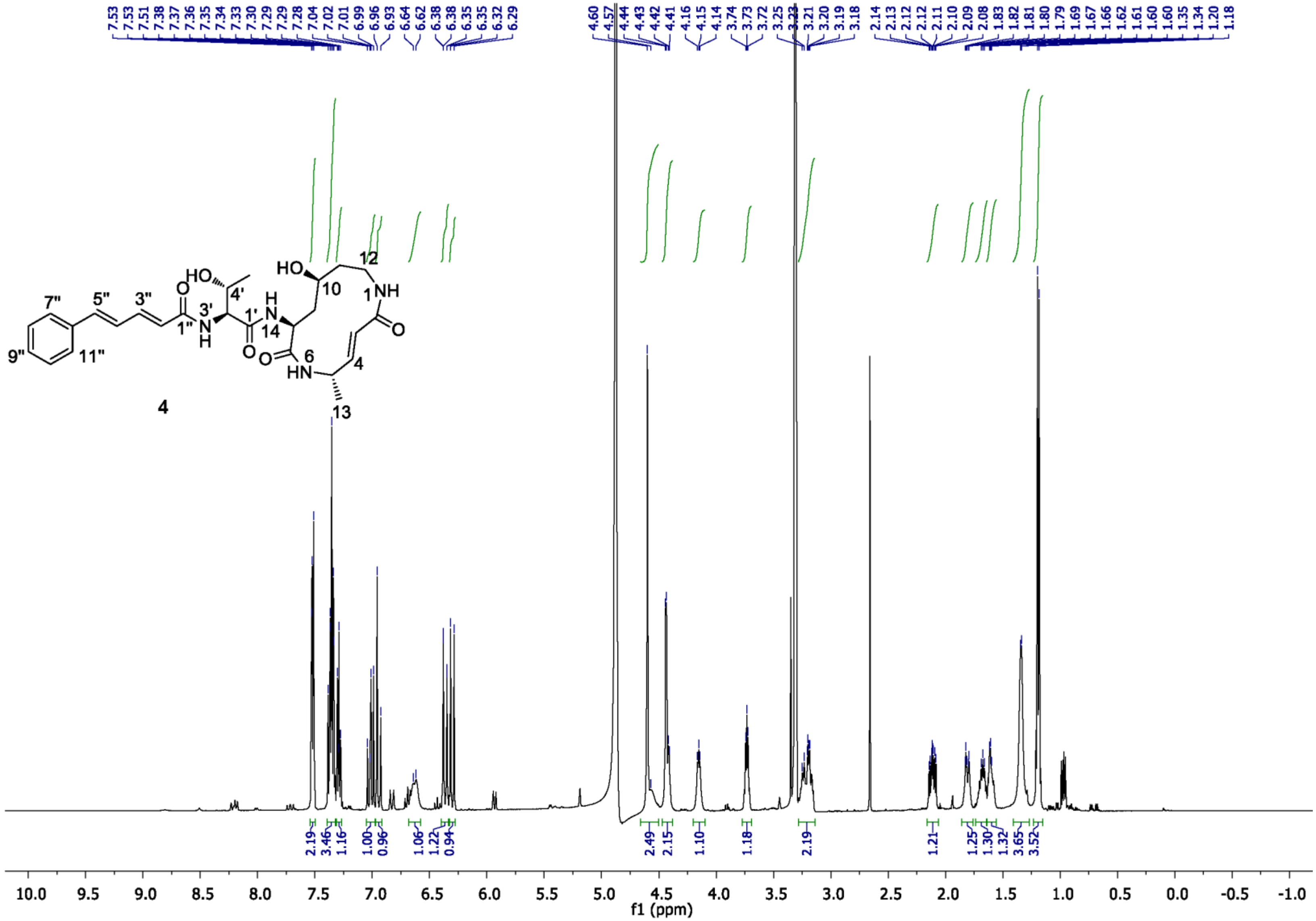

Figure S23. ^{1}H NMR (500 MHz, methanol-d_4) spectrum of **4**.

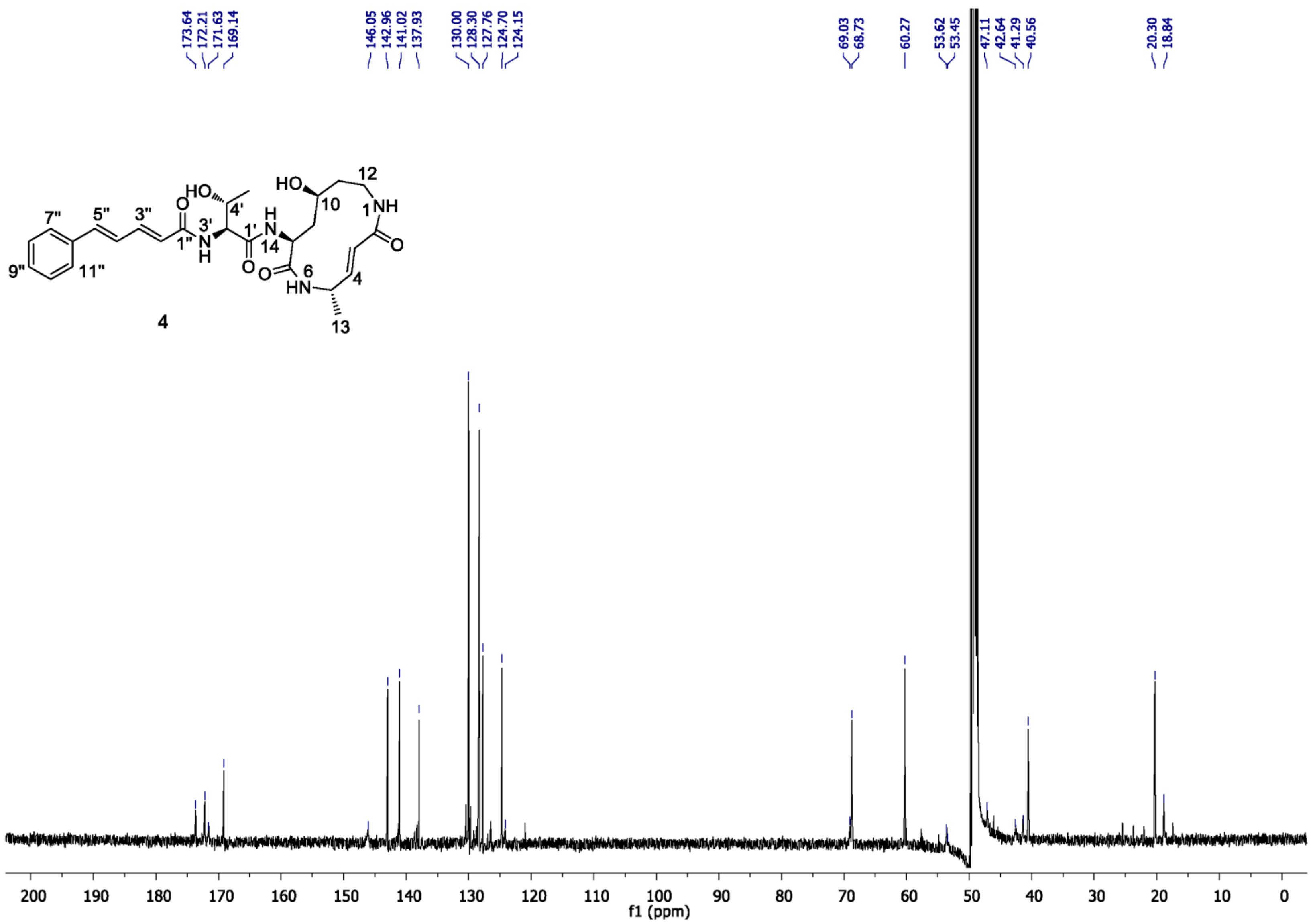

Figure S24. ^{13}C NMR (125 MHz, methanol-d4) spectrum of **4**.

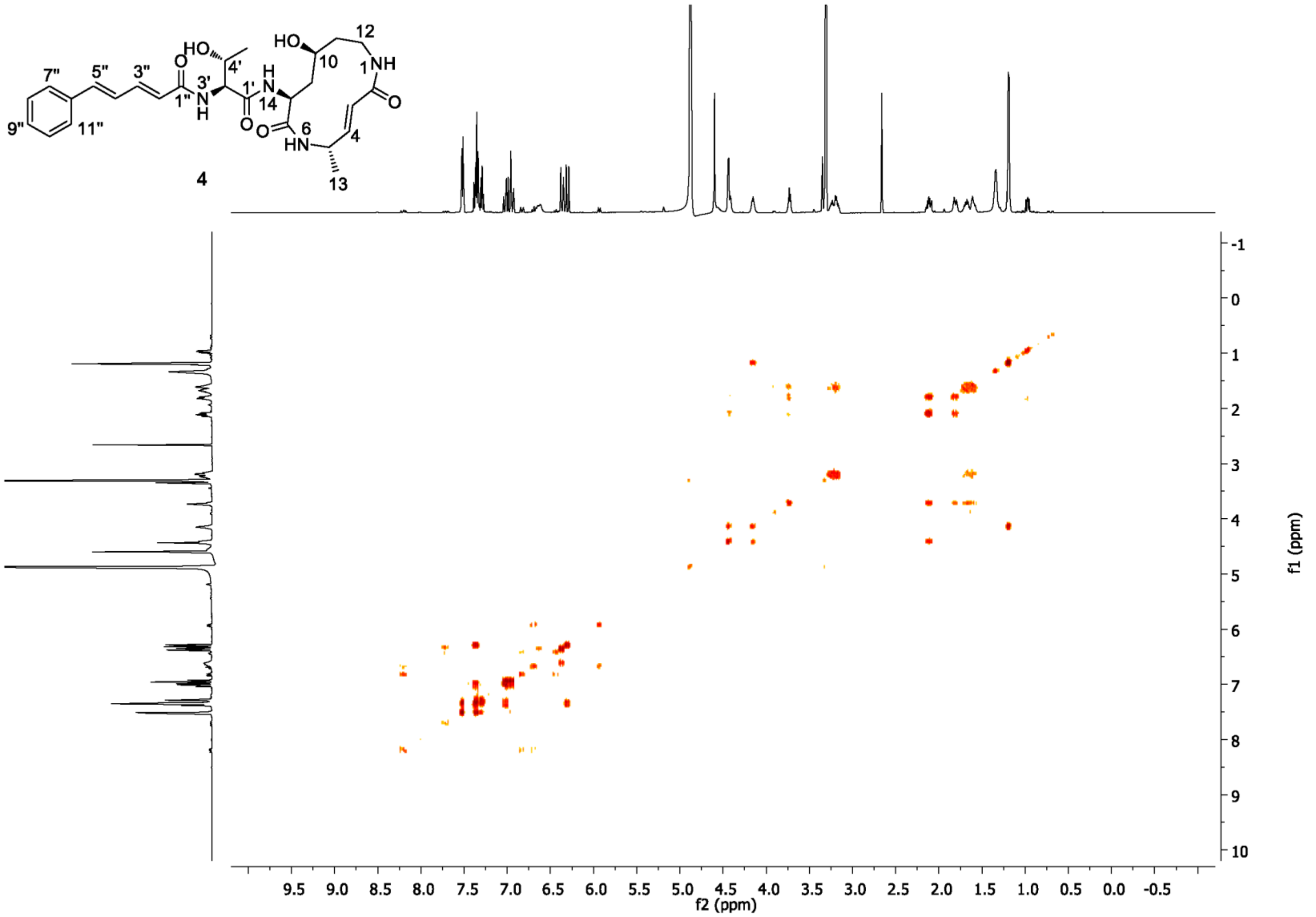

Figure S25. COSY (methanol-d$_4$) spectrum of **4**.

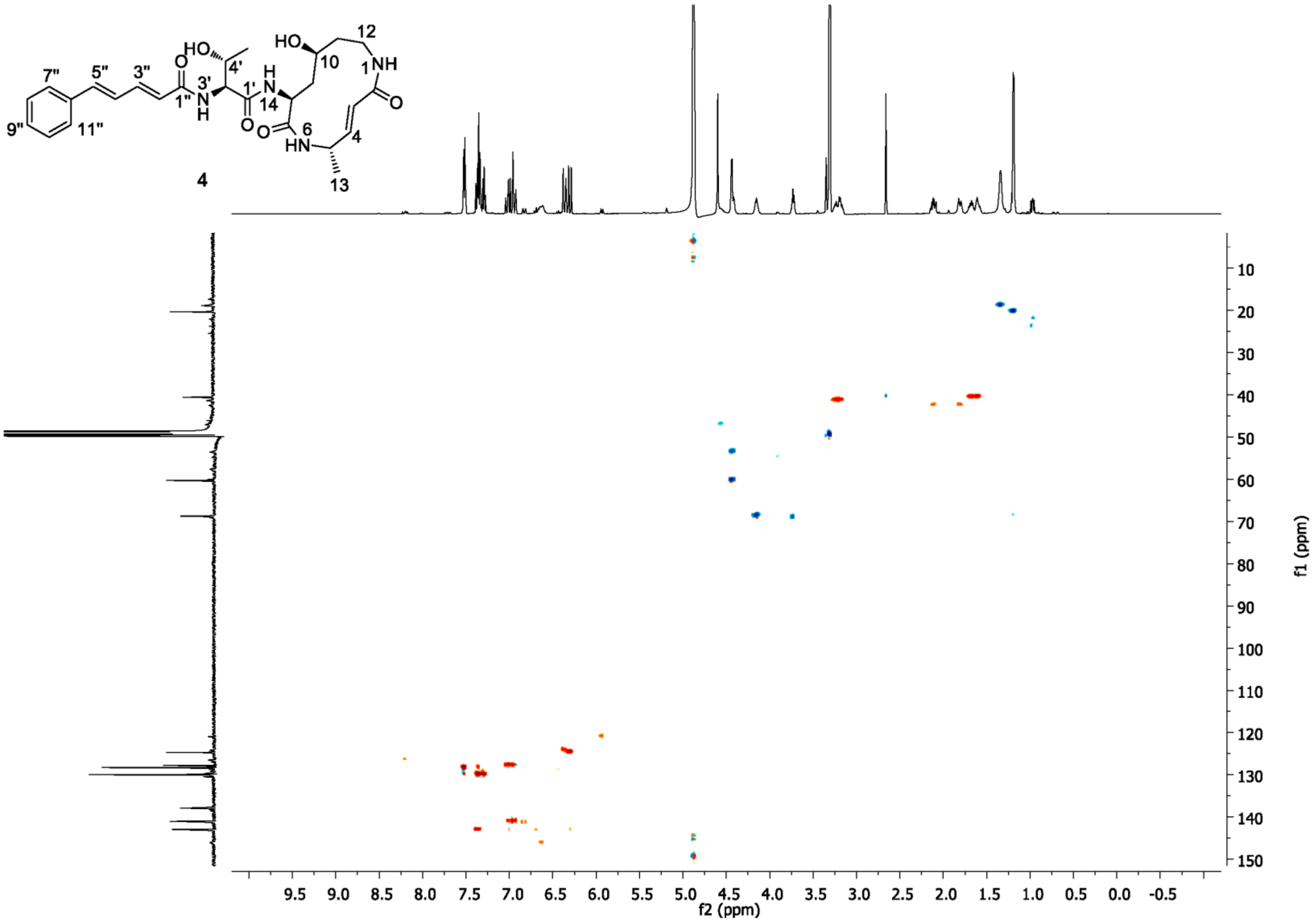

Figure S26. HSQC (methanol-d$_4$) spectrum of **4**.

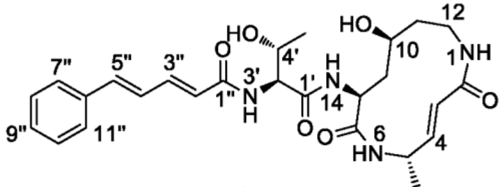

4

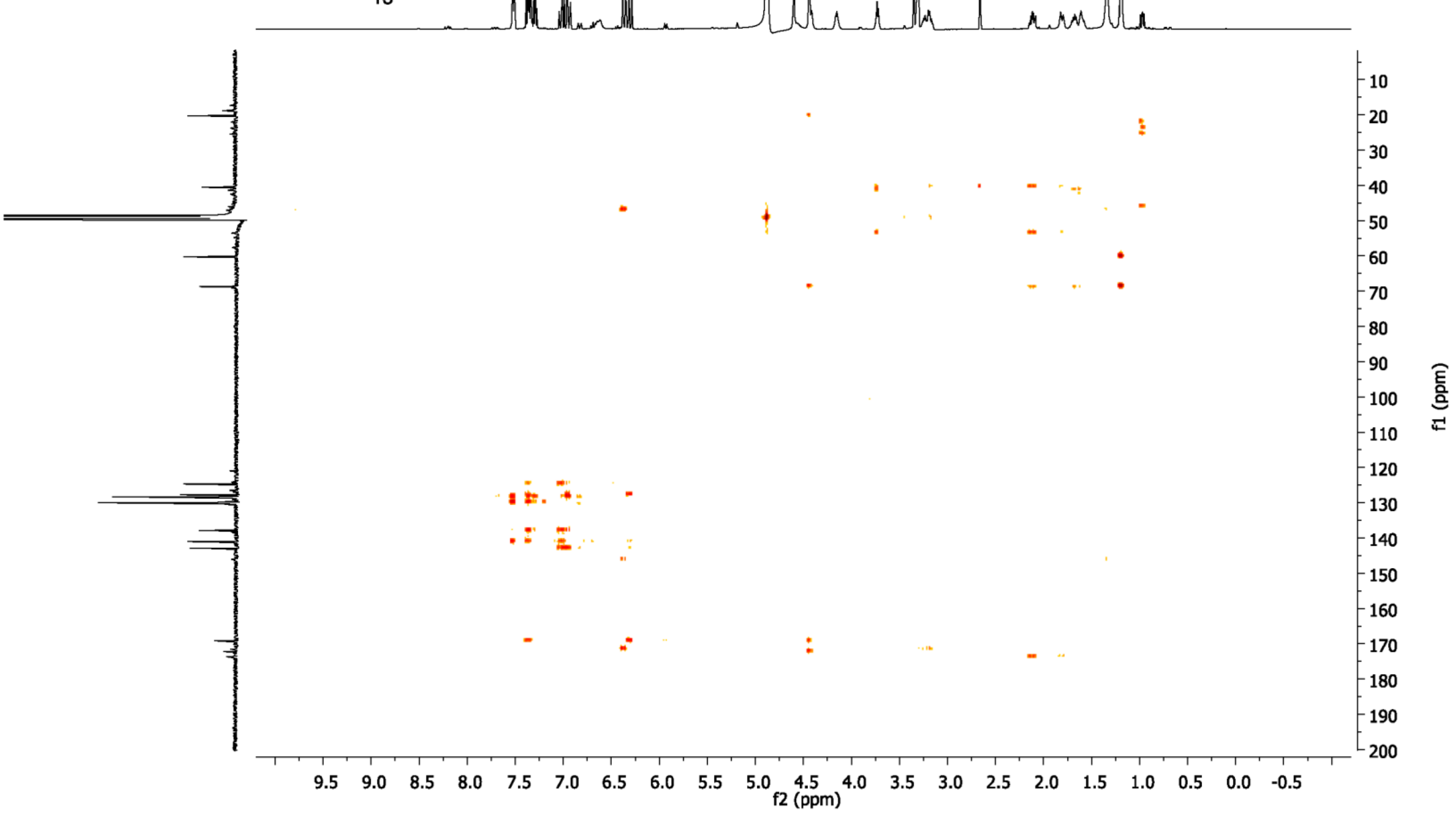

Figure S27. HMBC (methanol-d_4) spectrum of **4**.

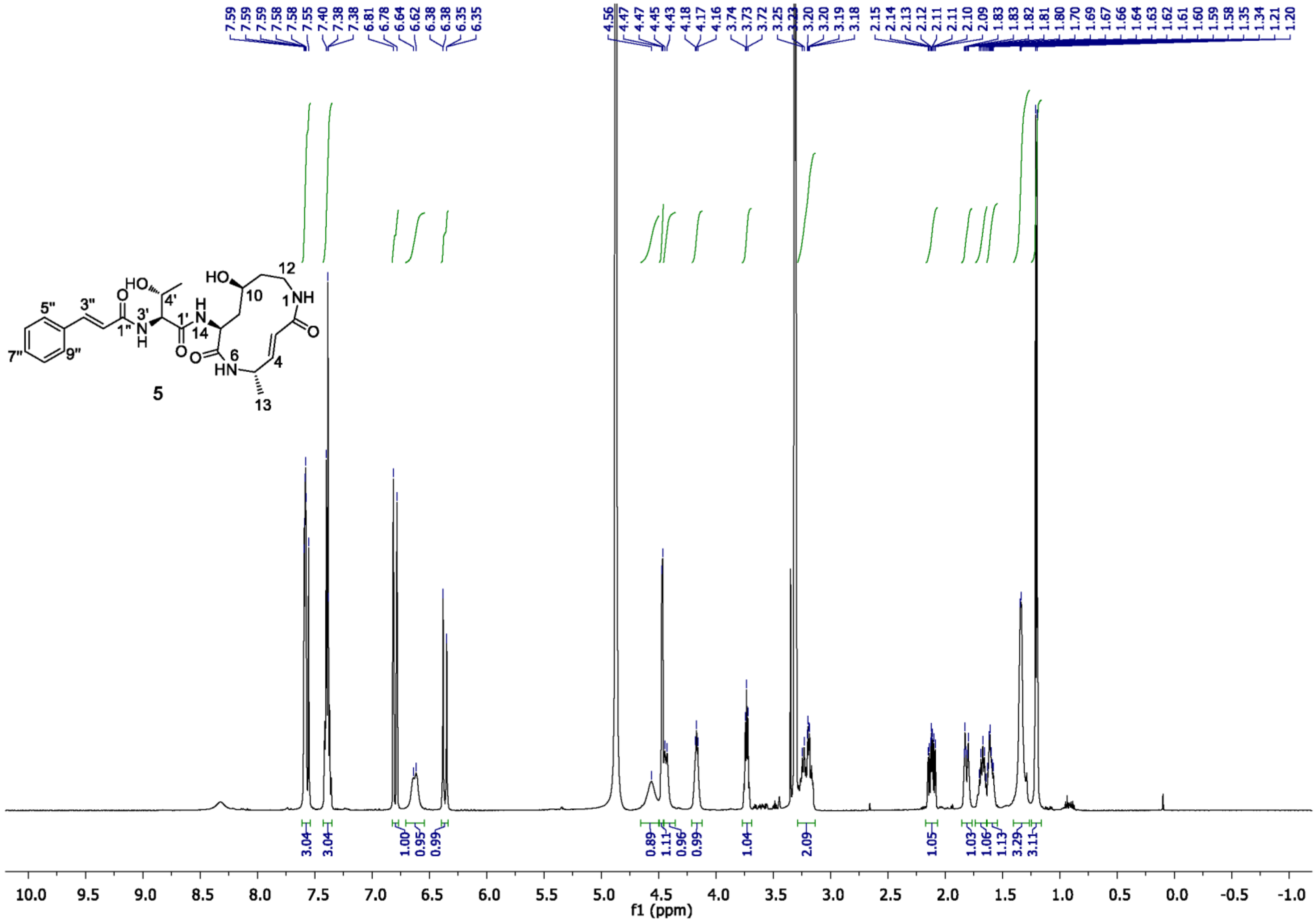

Figure S28. ^{1}H NMR (500 MHz, methanol-d4) spectrum of **5**.

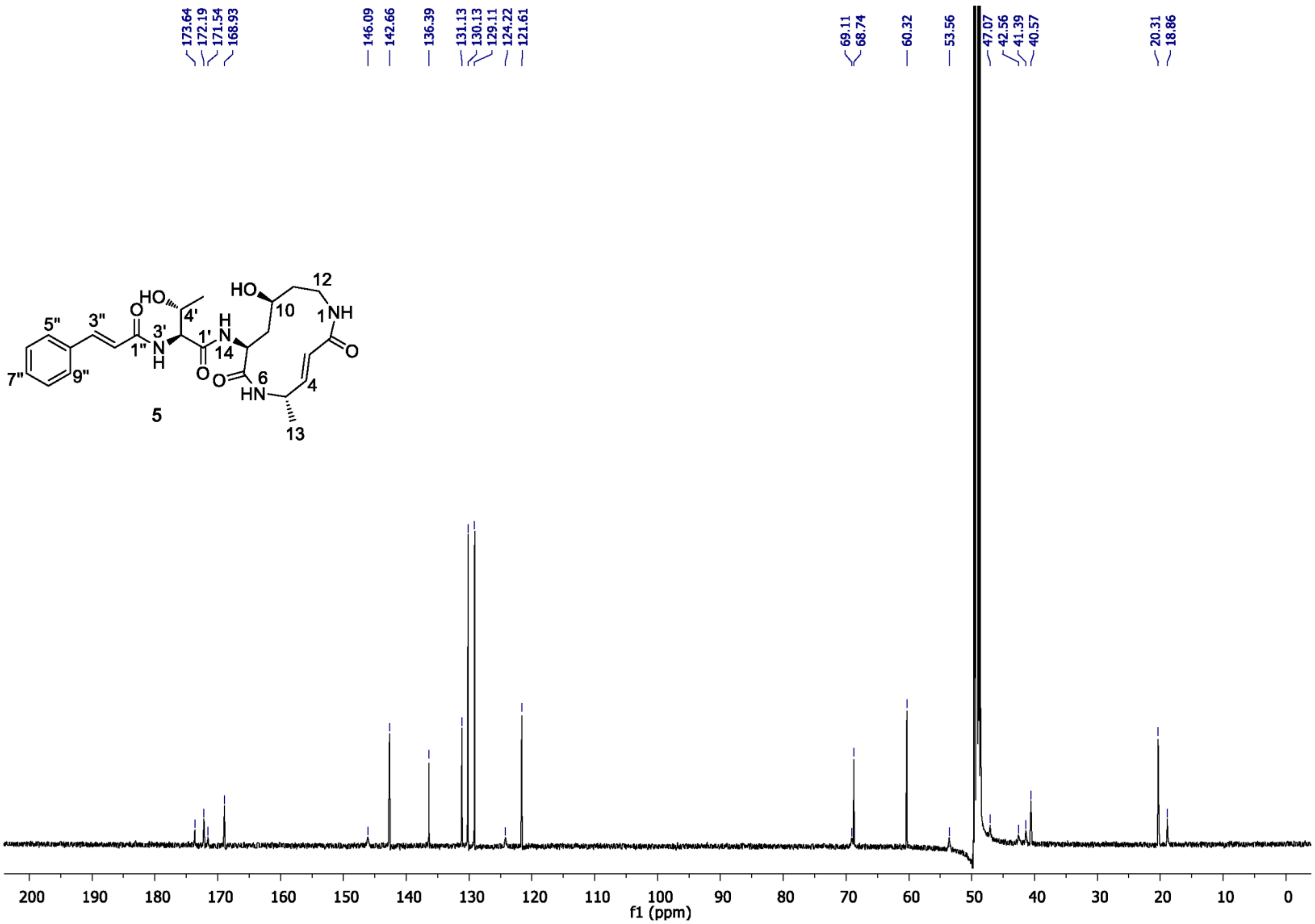

Figure S29. ^{13}C NMR (125 MHz, methanol-d$_4$) spectrum of **5**.

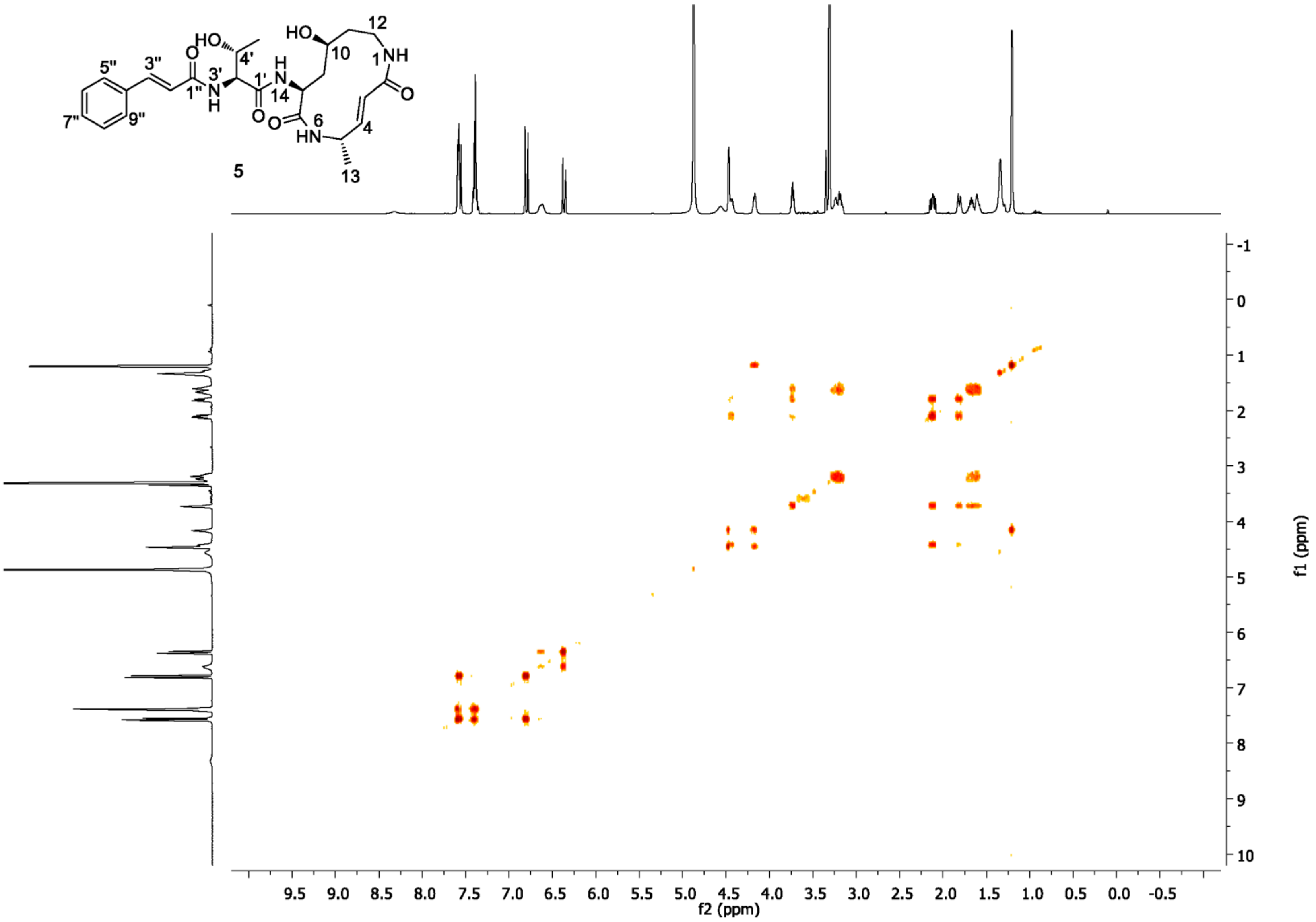

Figure S30. COSY (methanol-d4) spectrum of **5**.

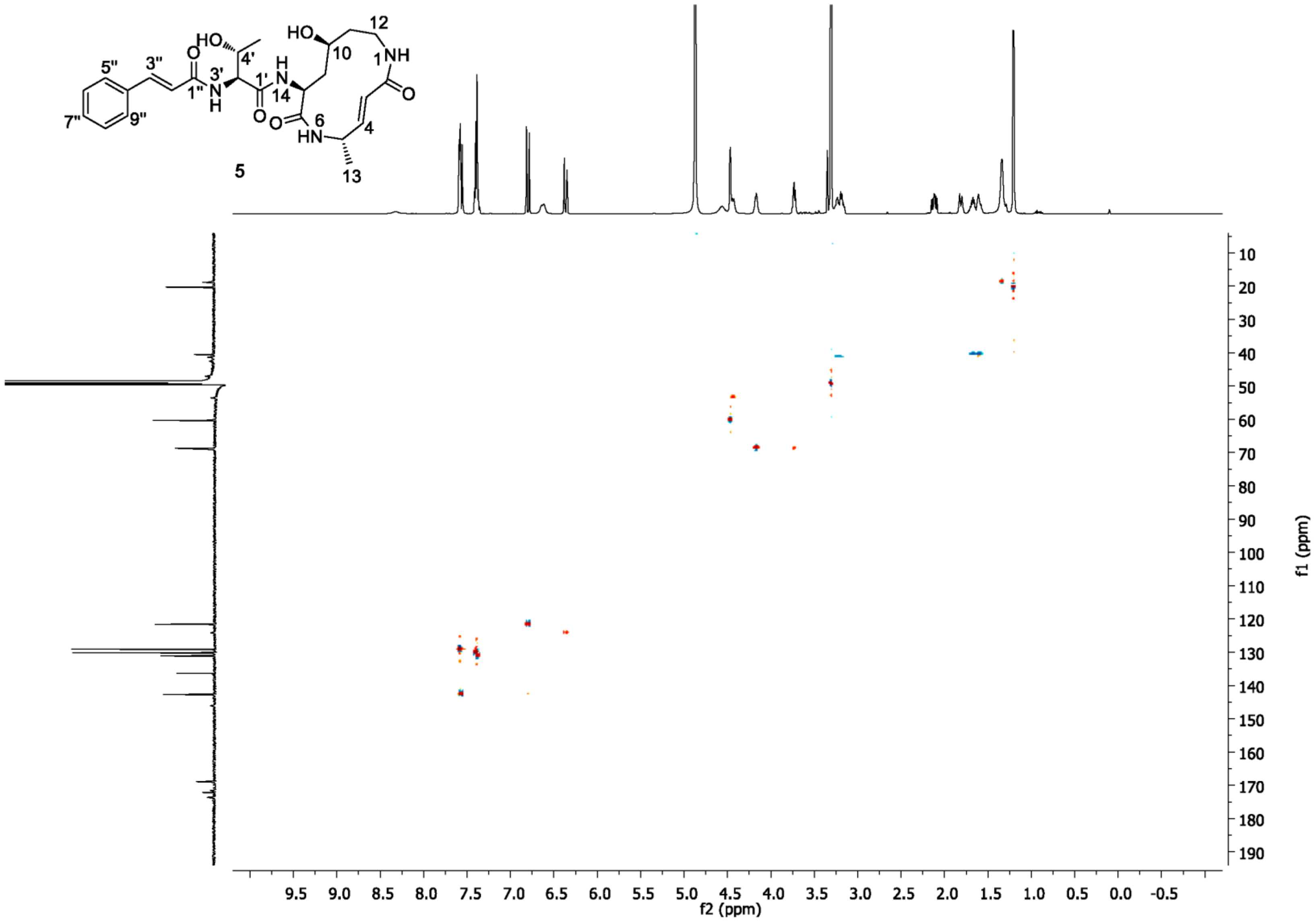

Figure S31. HSQC (methanol-d4) spectrum of **5**.

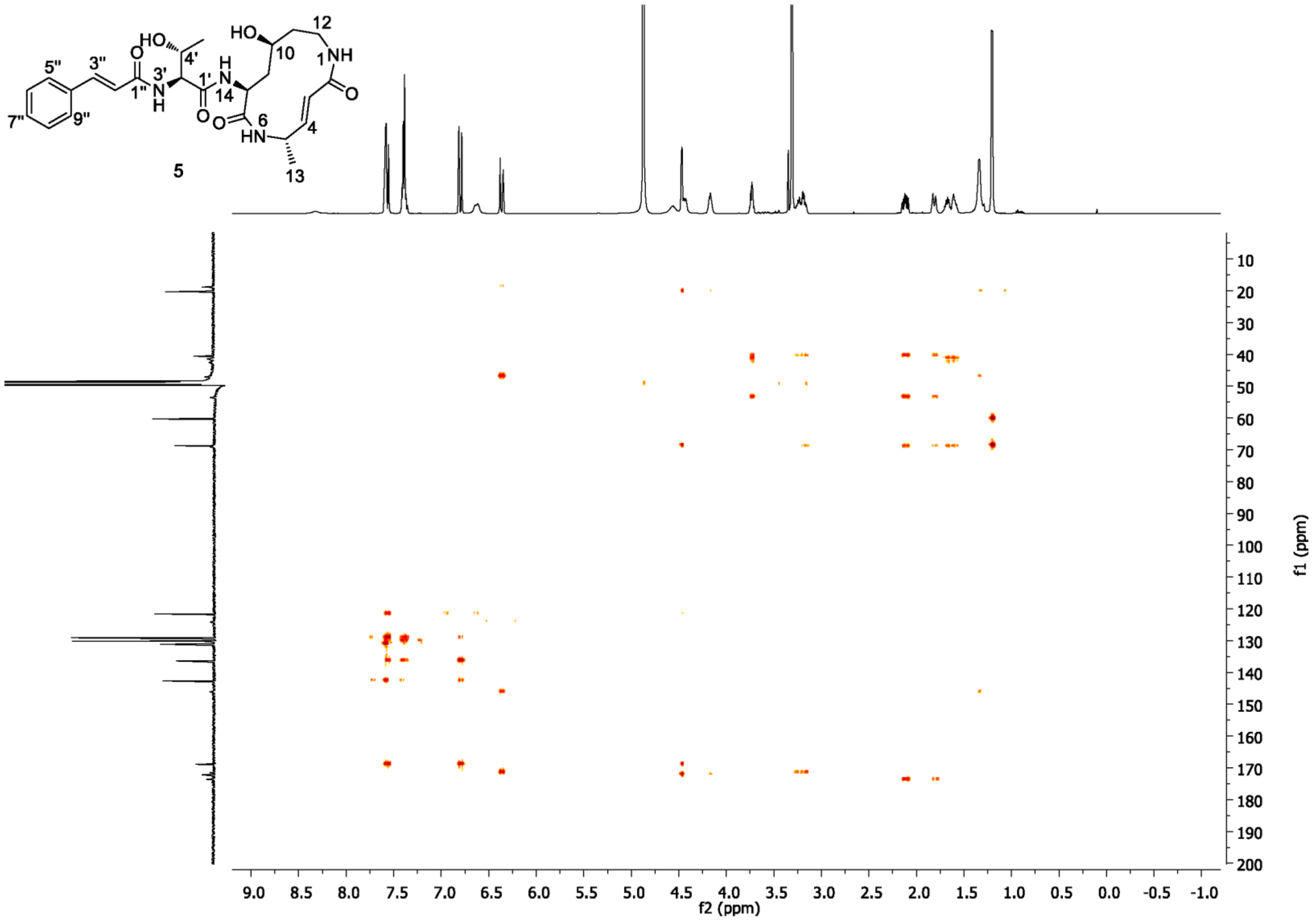

Figure S32. HMBC (methanol-d4) spectrum of **5**.

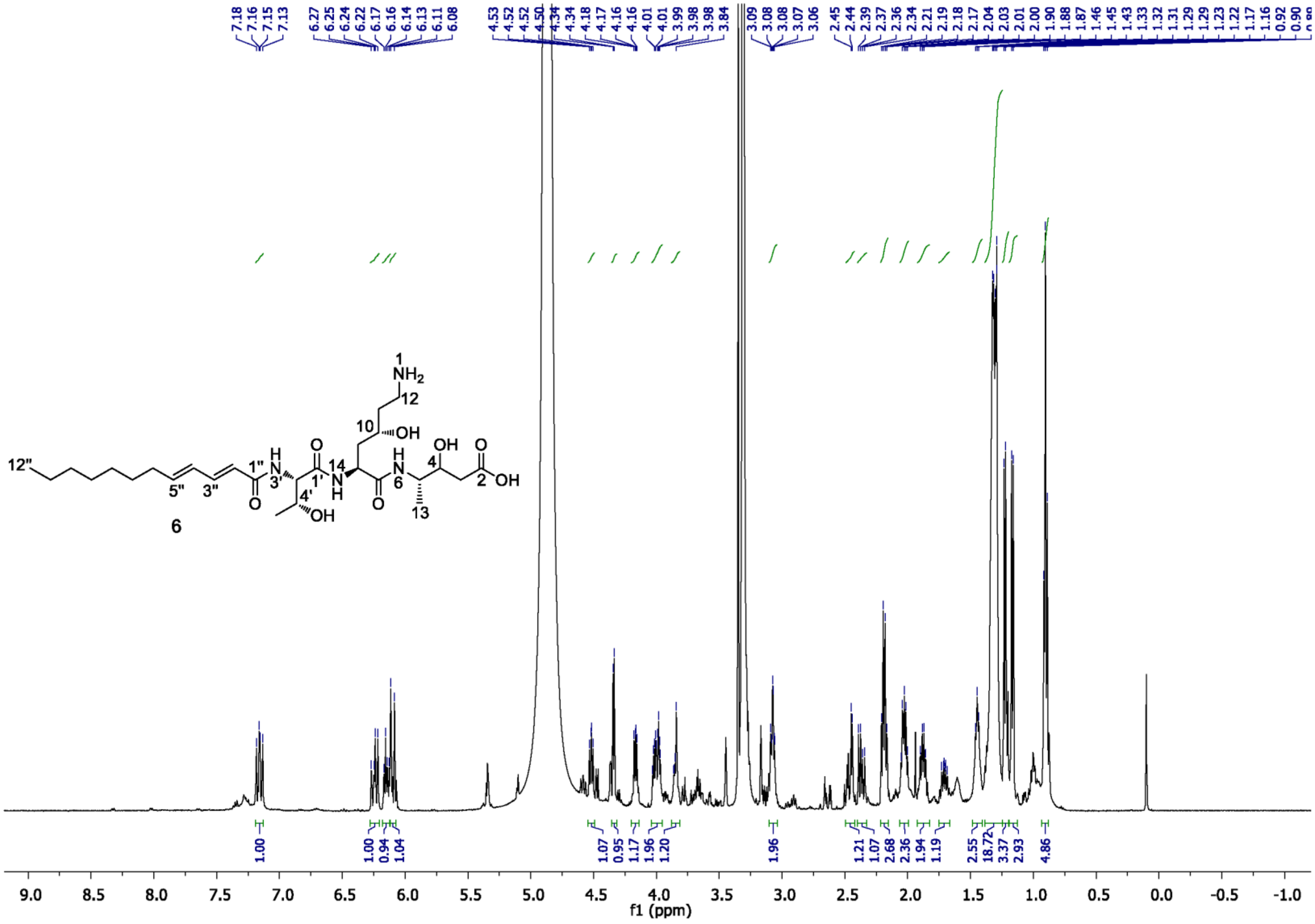

Figure S33. ^{1}H NMR (500 MHz, methanol-d_4) spectrum of **6**.

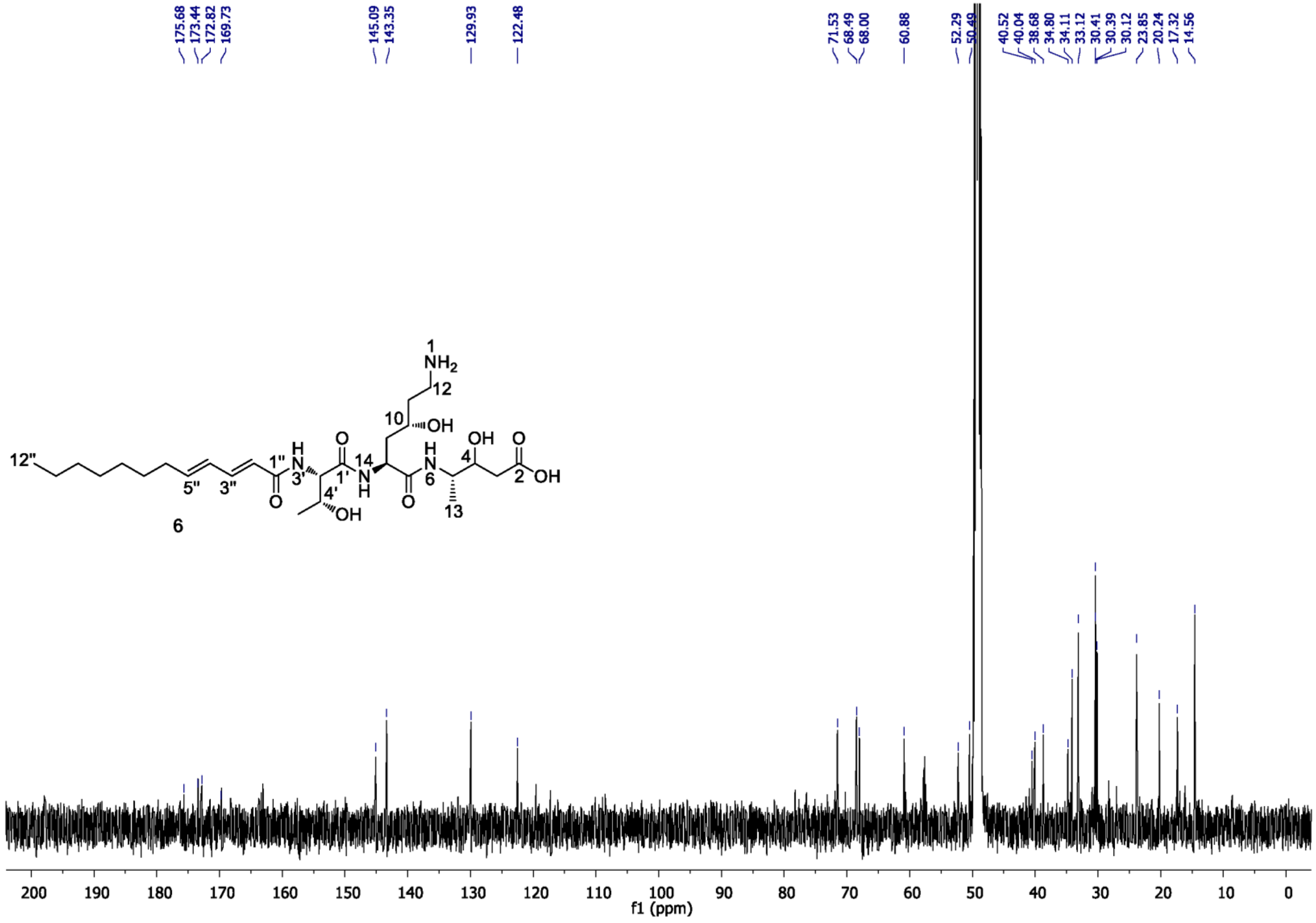

Figure S34. ^{13}C NMR (125 MHz, methanol-d_4) spectrum of **6**.

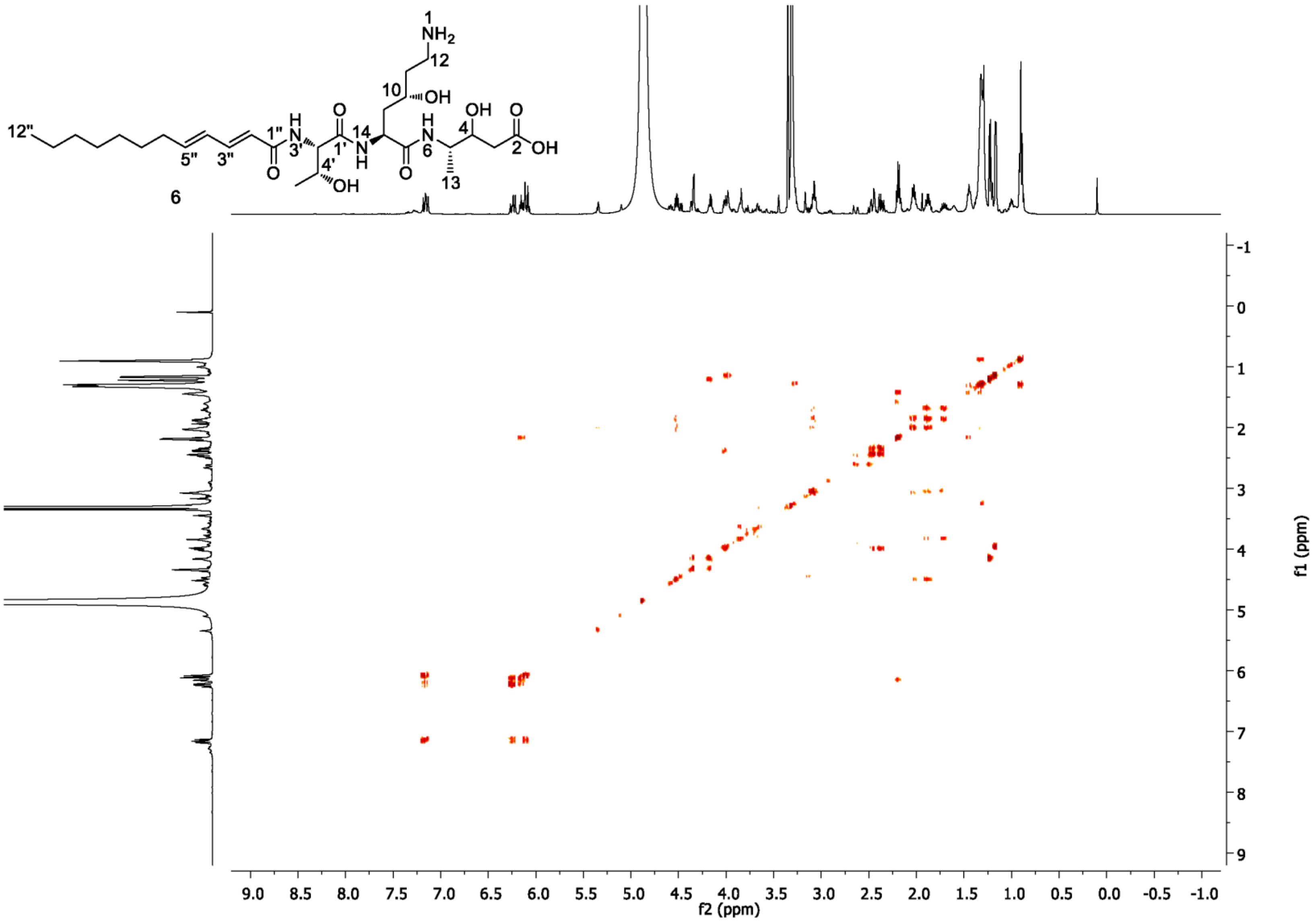

Figure S35. COSY (methanol-d4) spectrum of **6**.

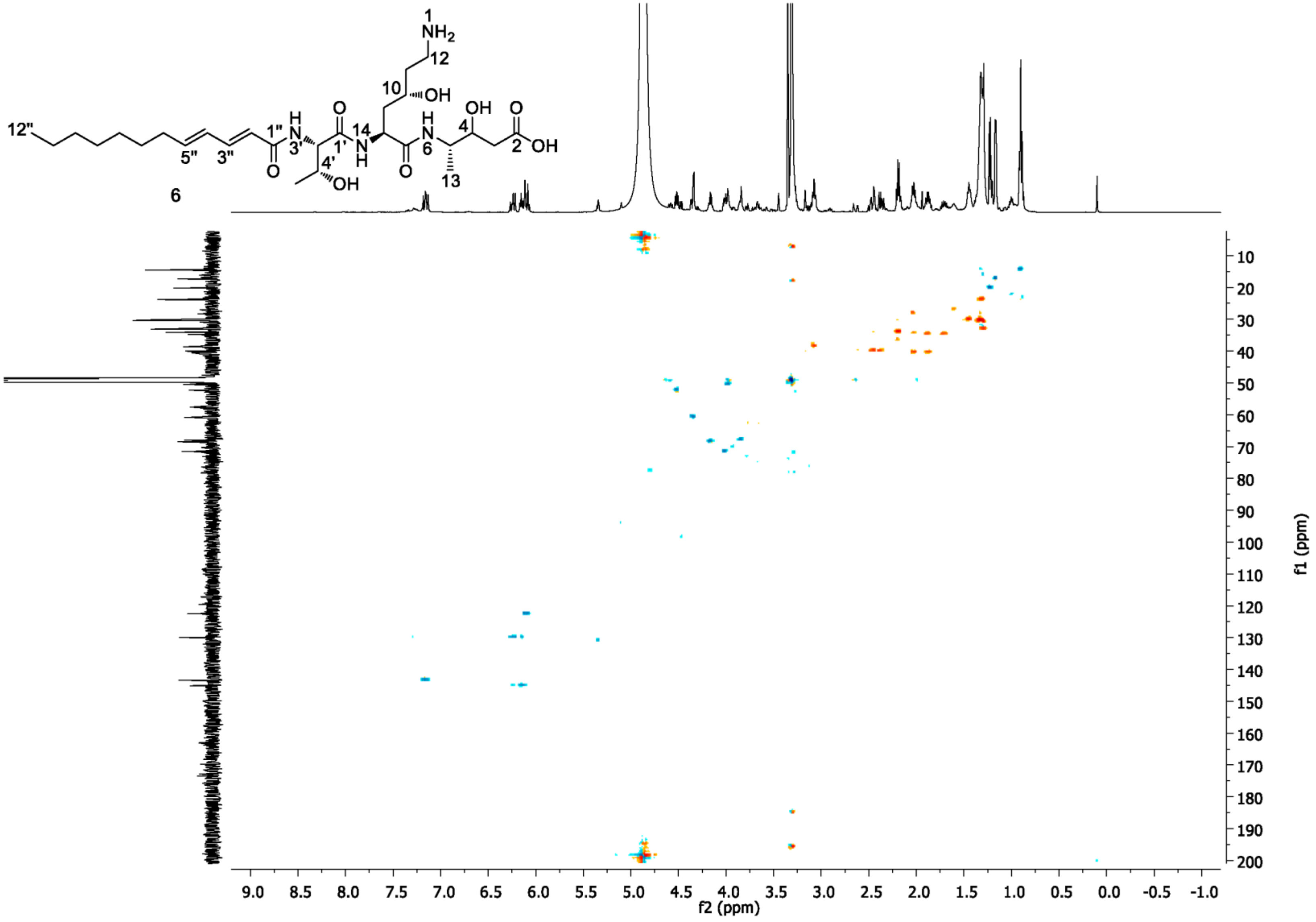

Figure S36. HSQC (methanol-d4) spectrum of **6**.

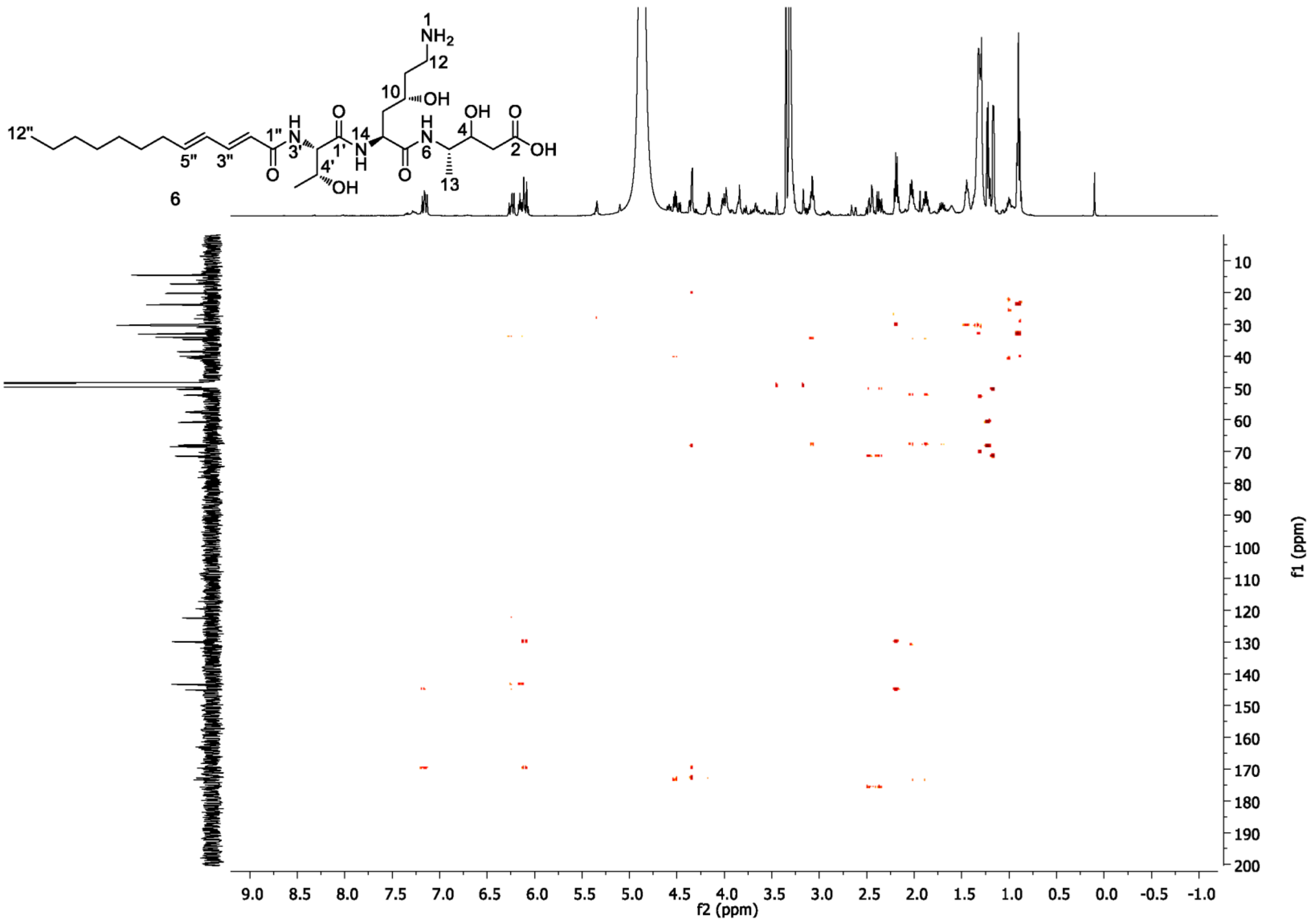

Figure S37. HMBC (methanol-d_4) spectrum of **6**.

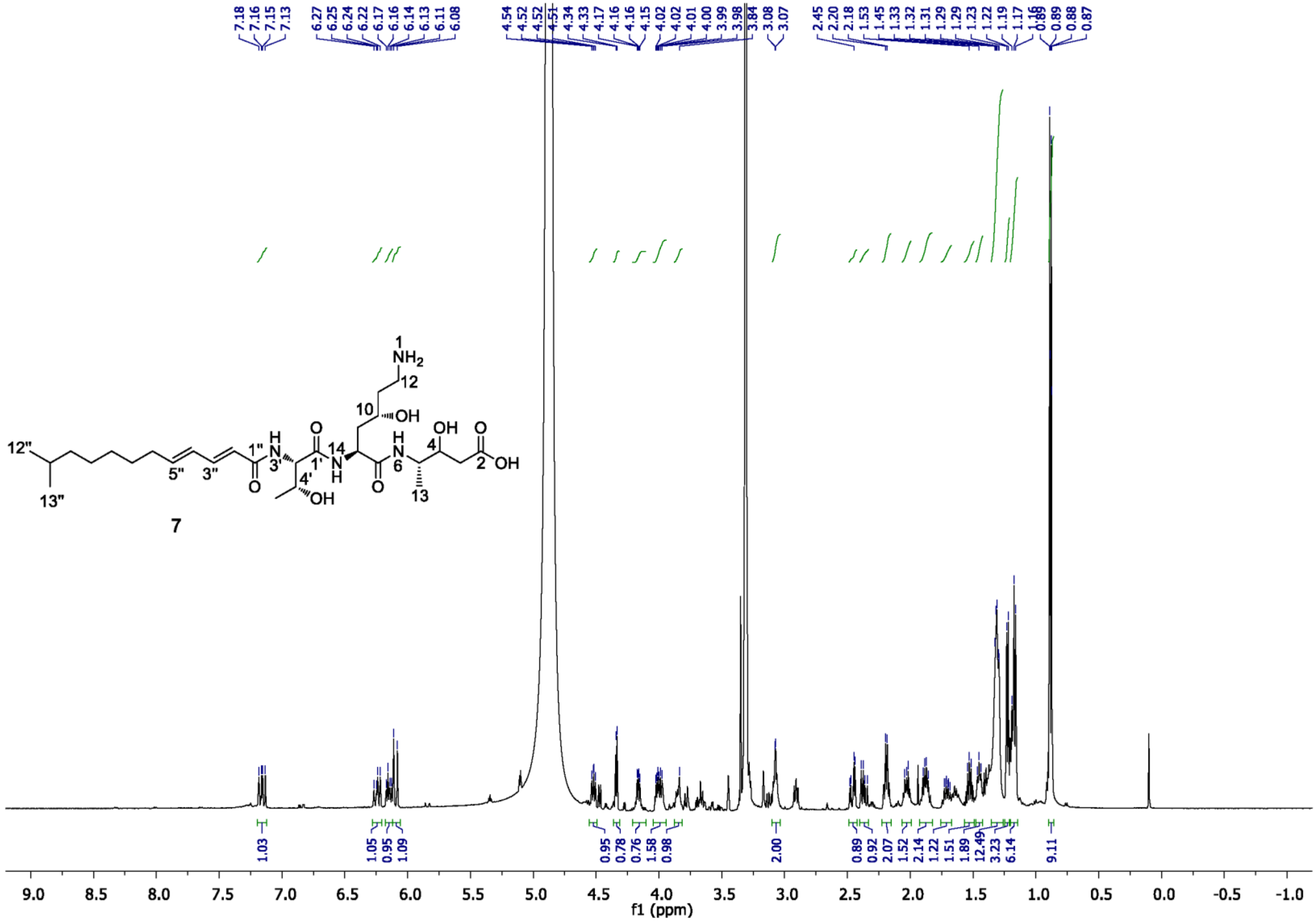

Figure S38. ^{1}H NMR (500 MHz, methanol-d$_4$) spectrum of **7**.

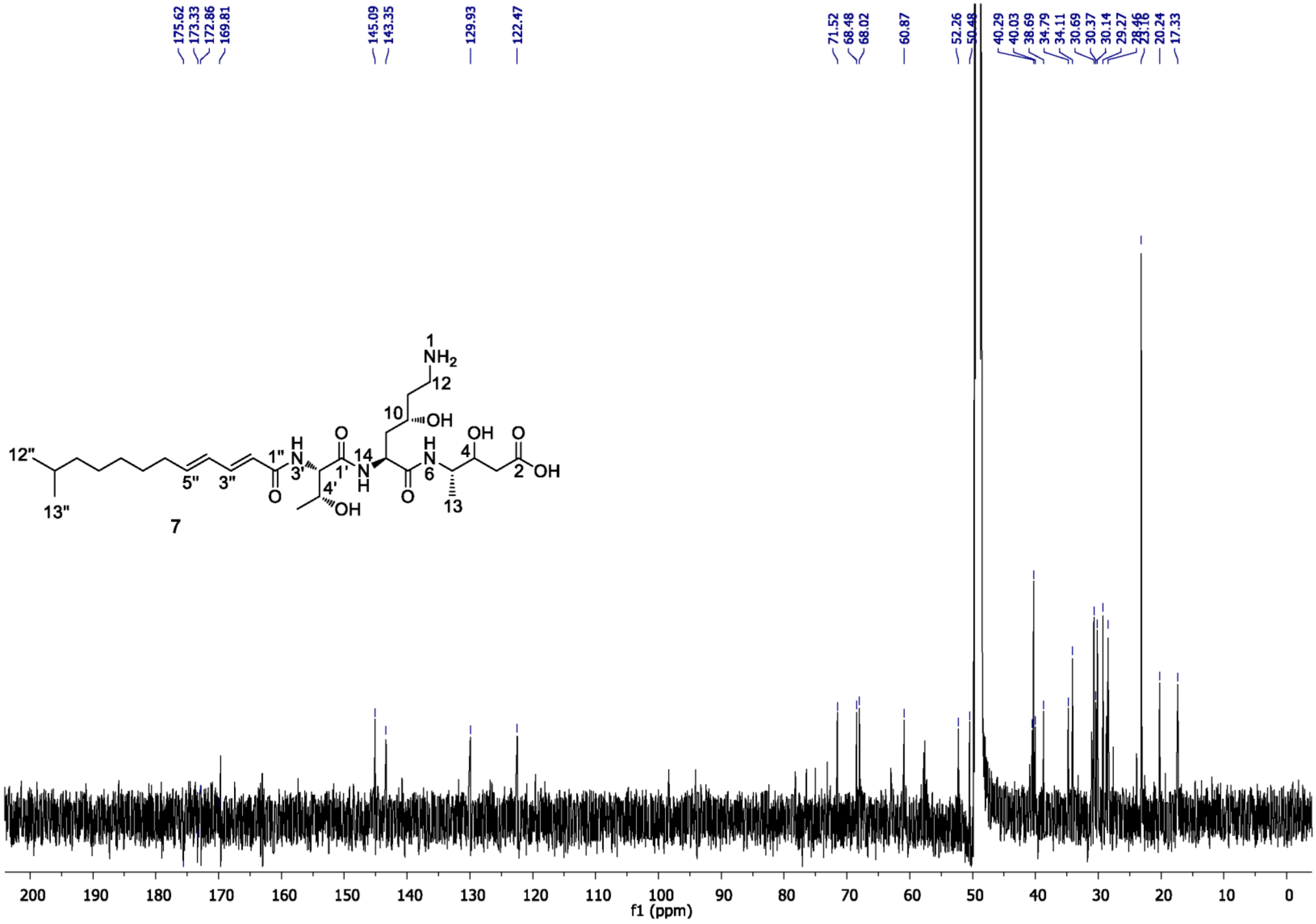

Figure S39. ^{13}C NMR (125 MHz, methanol-d_4) spectrum of **7**.

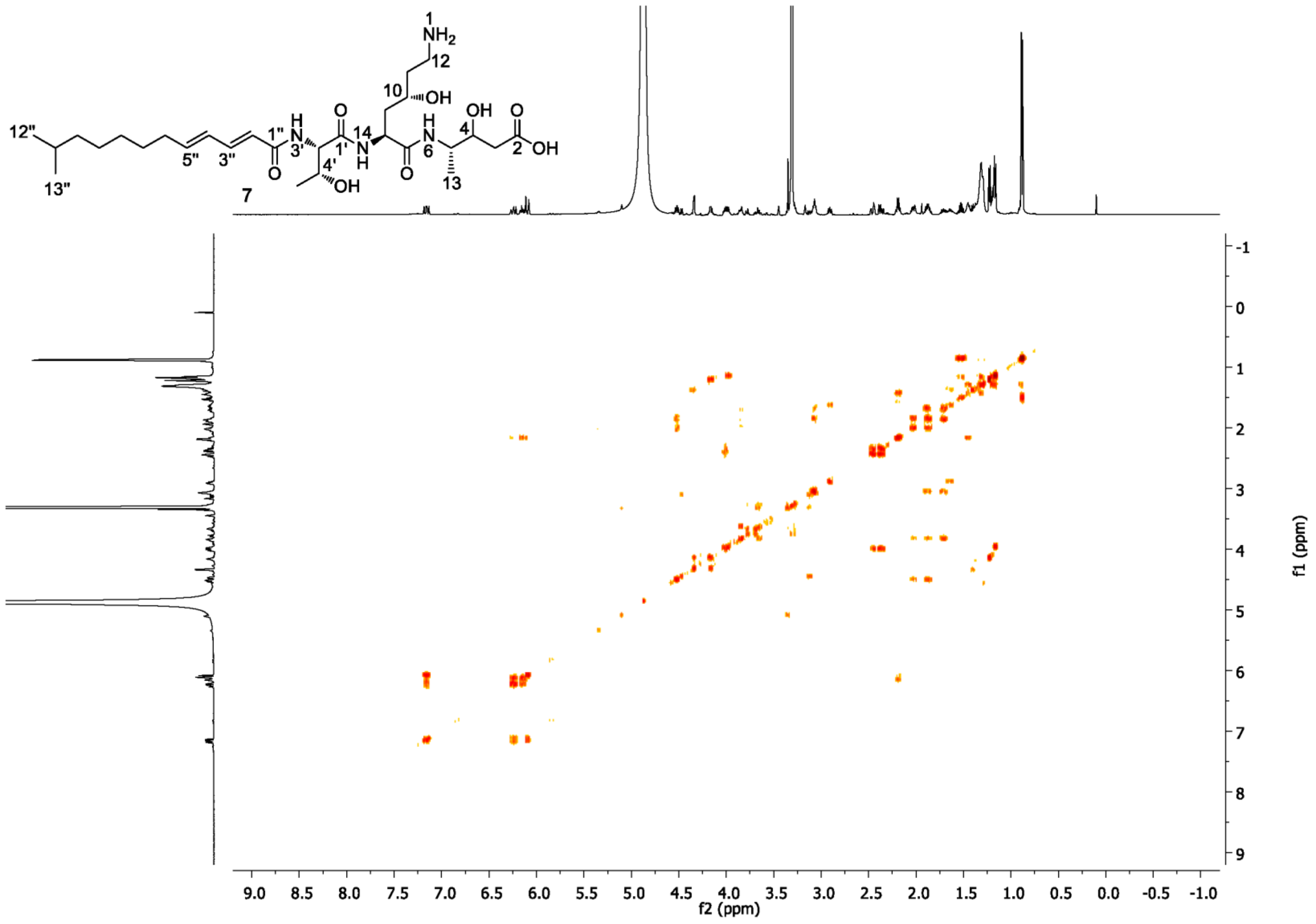

Figure S40. COSY (methanol-d$_4$) spectrum of **7**.

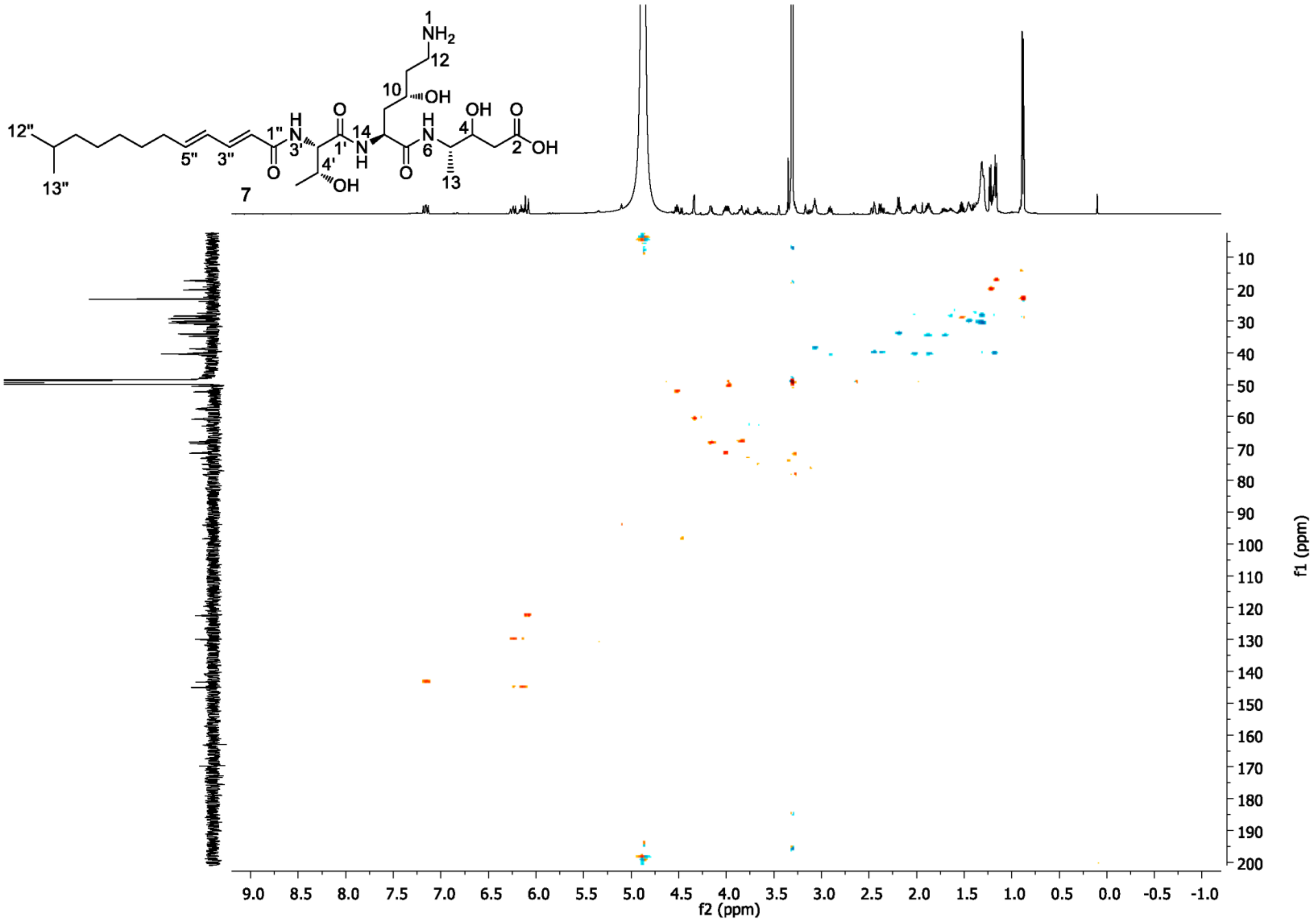

Figure S41. HSQC (methanol-d4) spectrum of **7**.

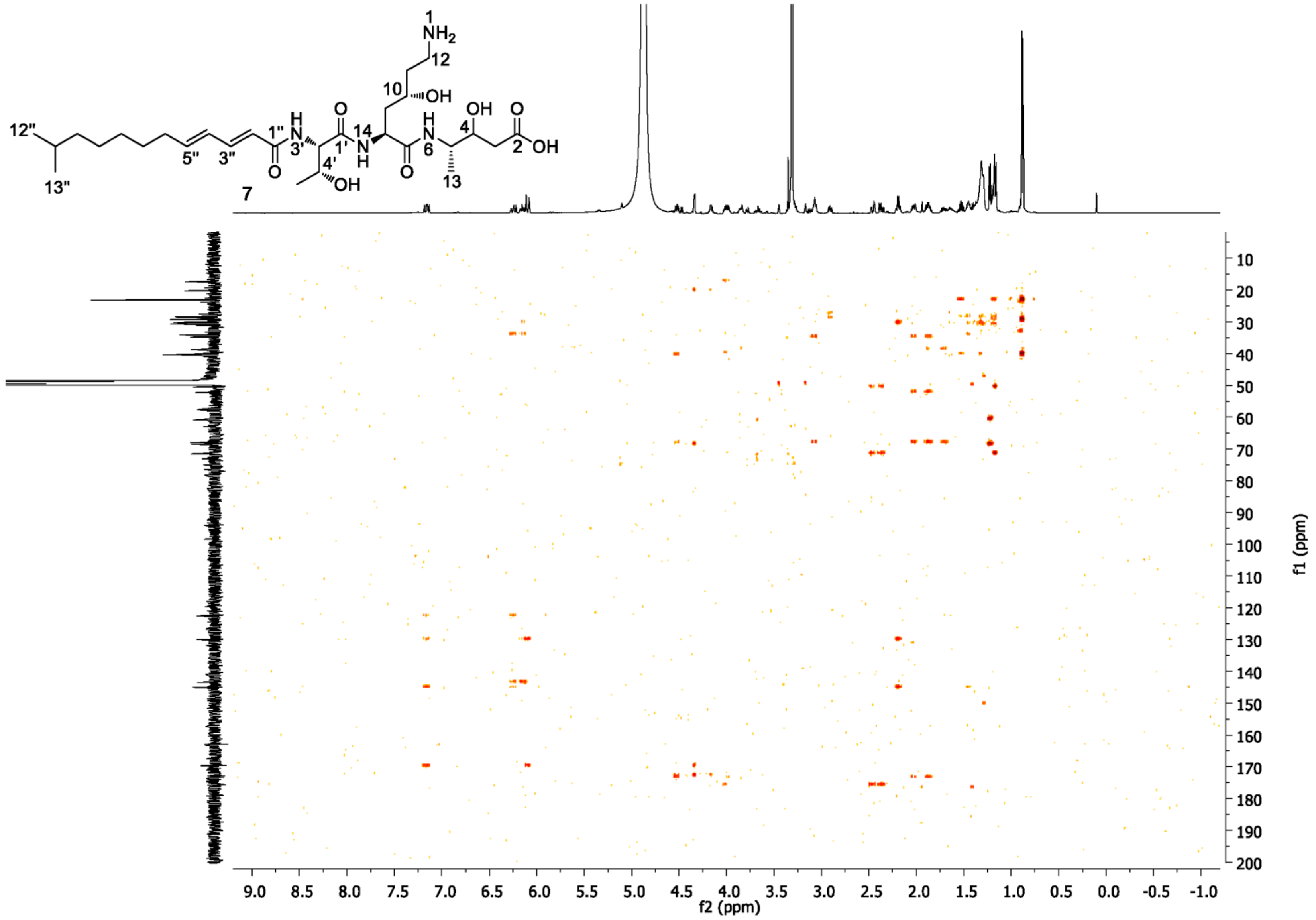

Figure S42. HMBC (methanol-d4) spectrum of **7**.

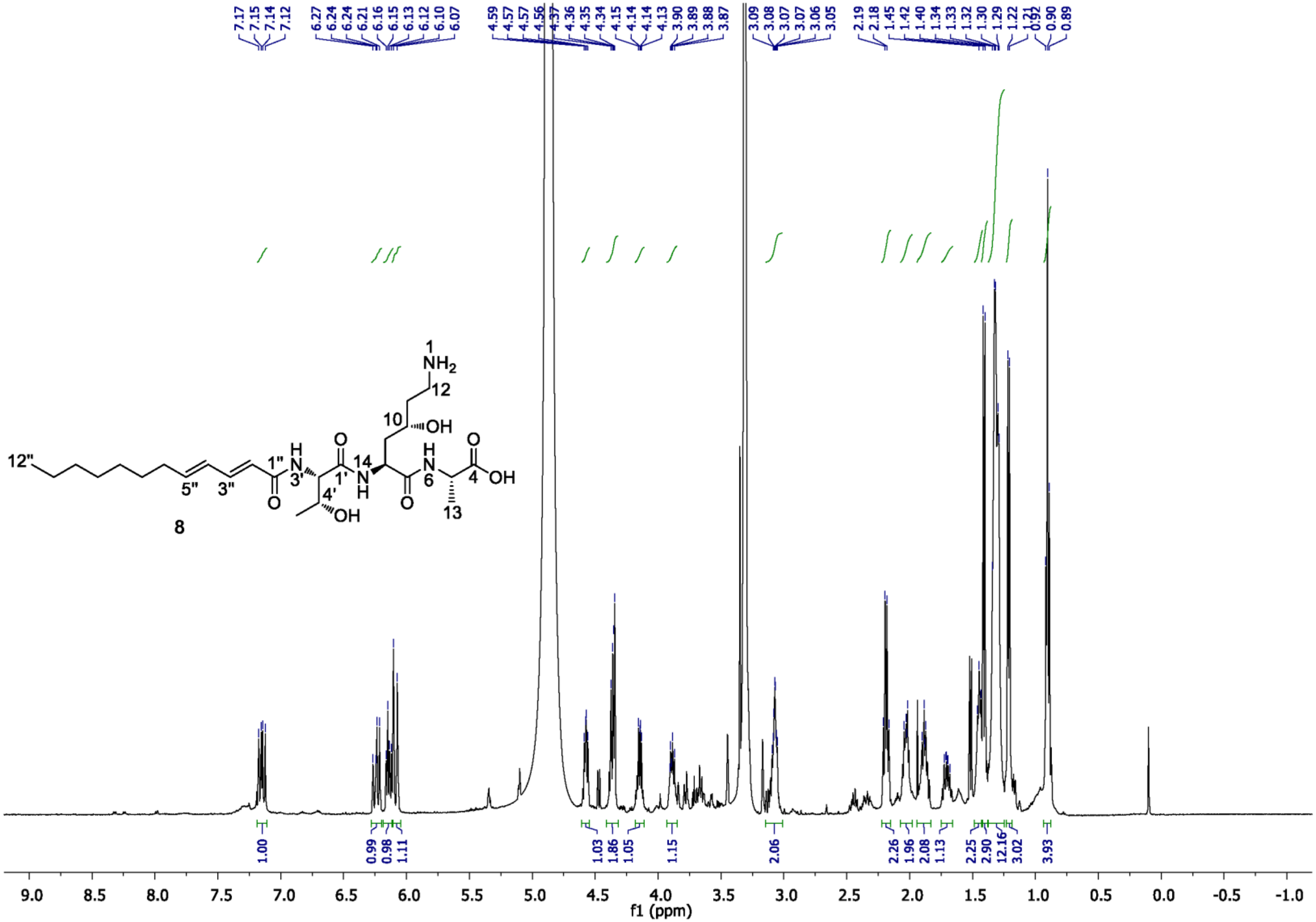

Figure S43. ^{1}H NMR (500 MHz, methanol-d_4) spectrum of **8**.

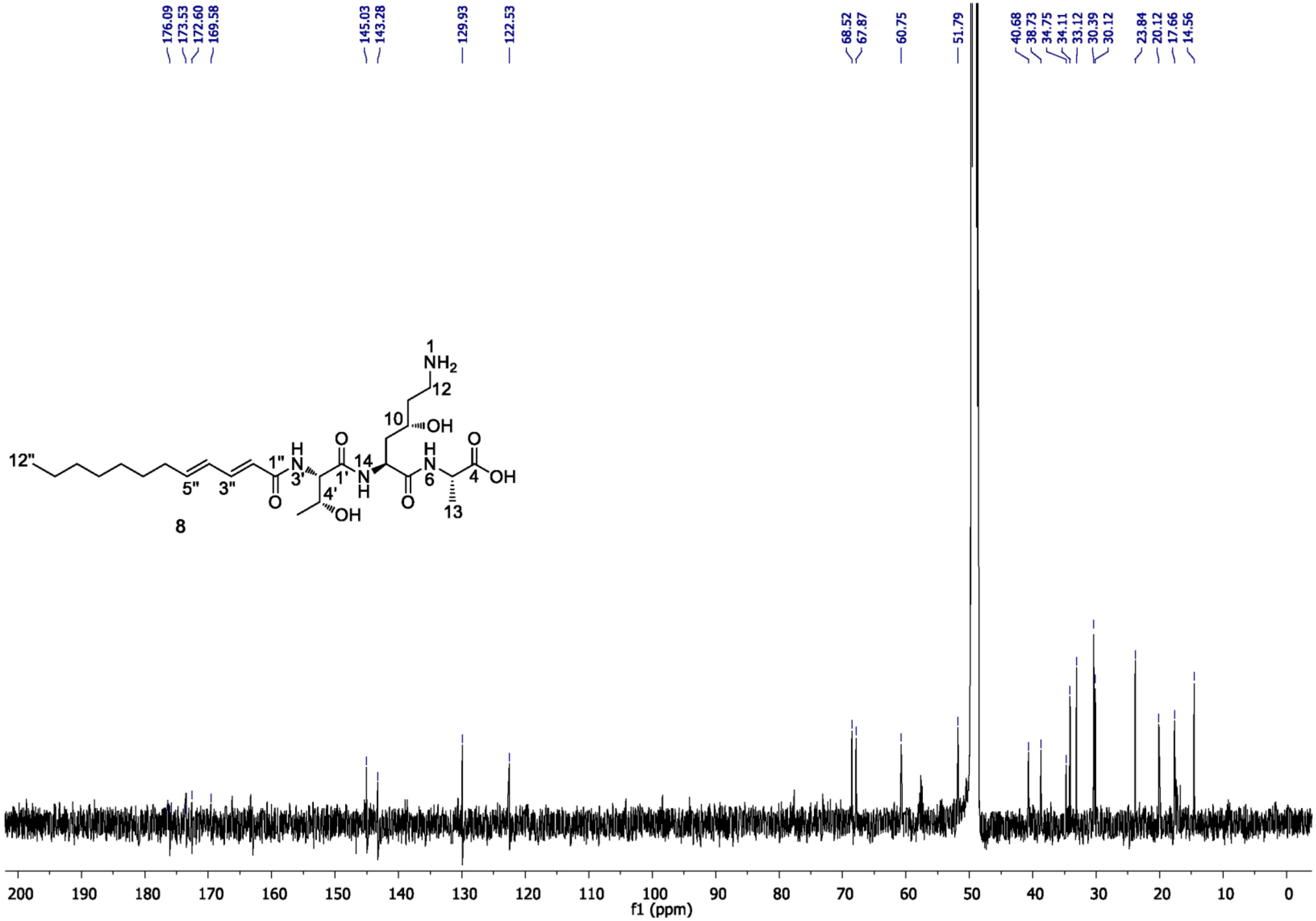

Figure S44. ^{13}C NMR (125 MHz, methanol-d$_4$) spectrum of **8**.

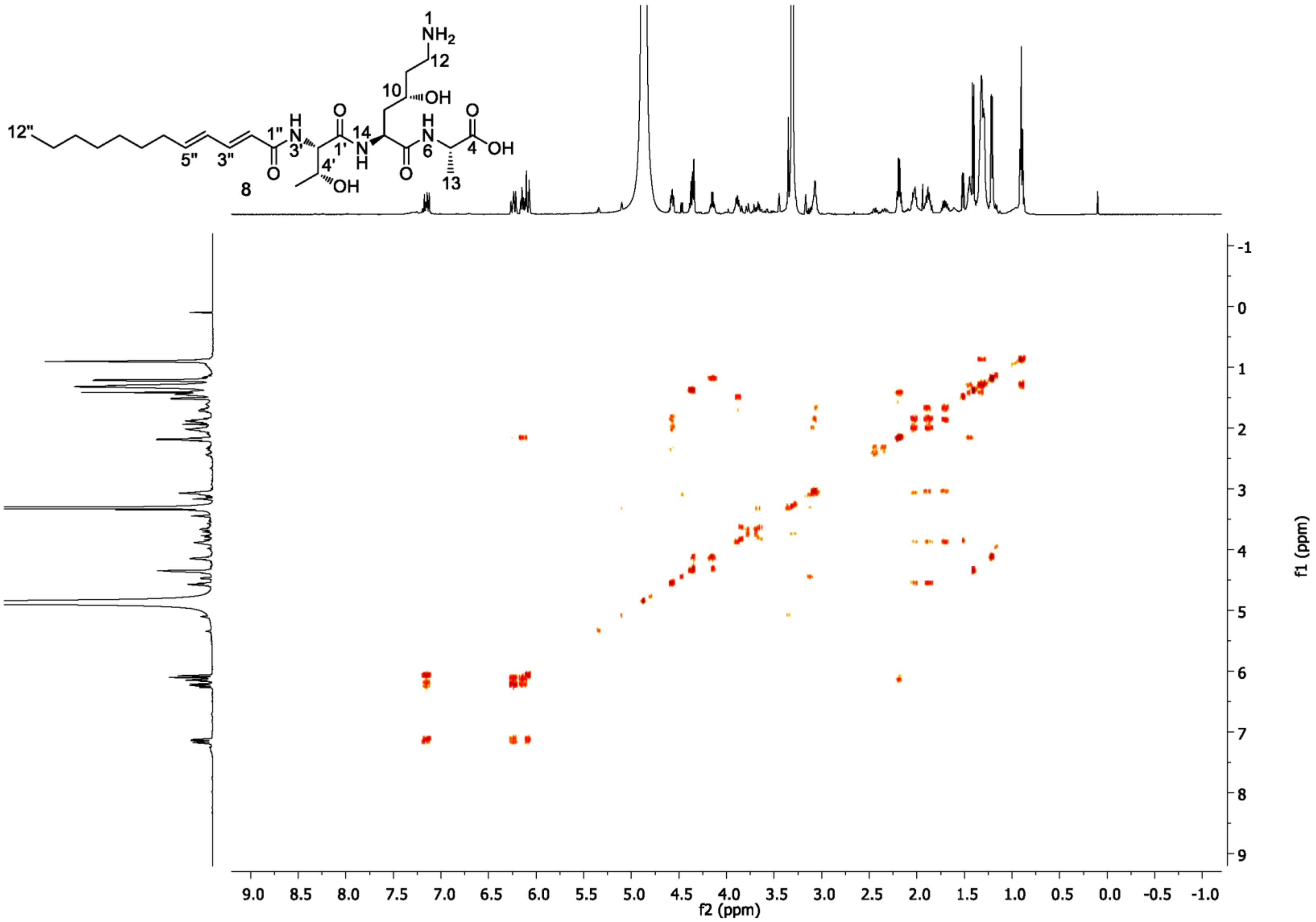

Figure S45. COSY (methanol-d4) spectrum of **8**.

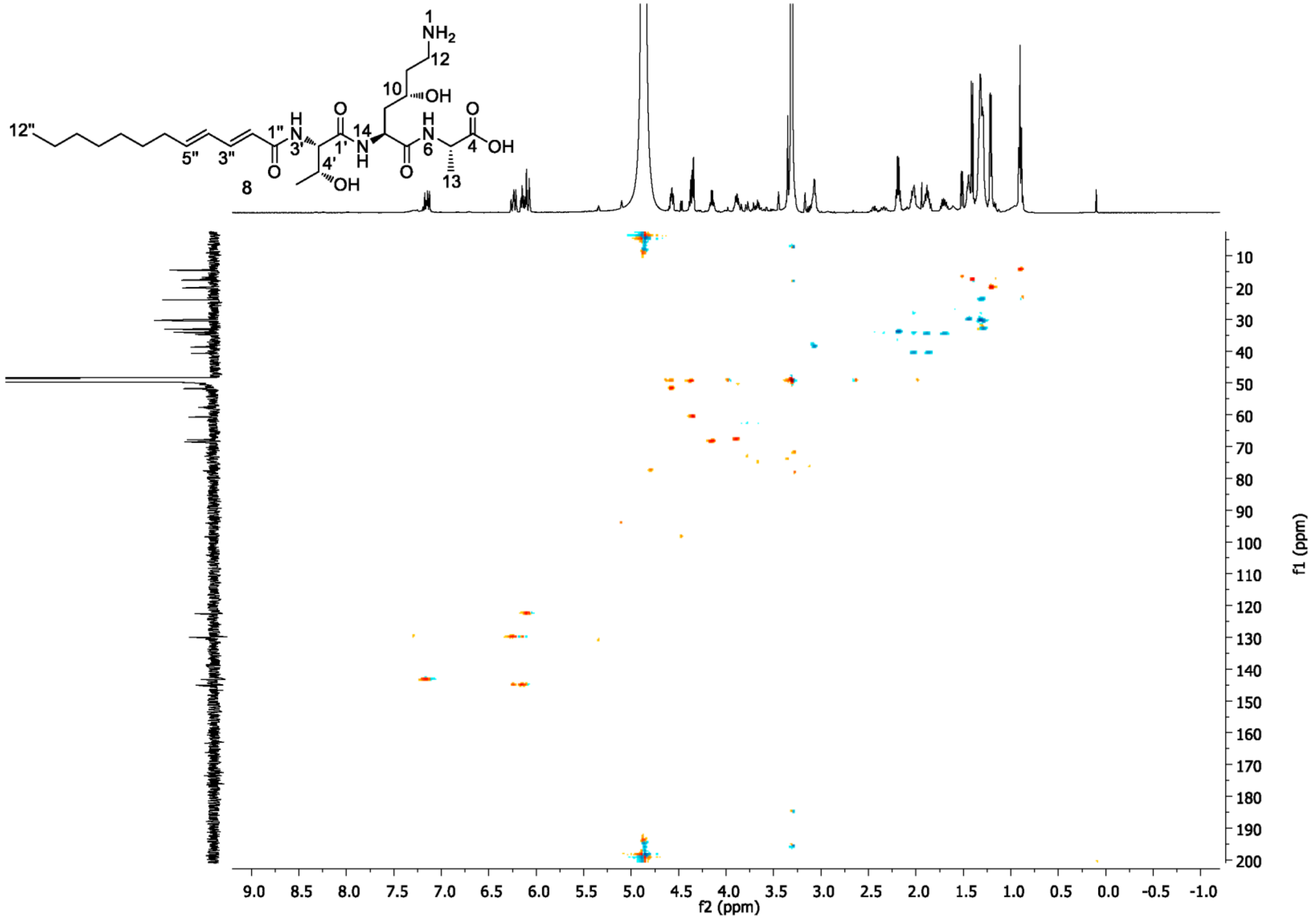

Figure S46. HSQC (methanol-d4) spectrum of **8**.

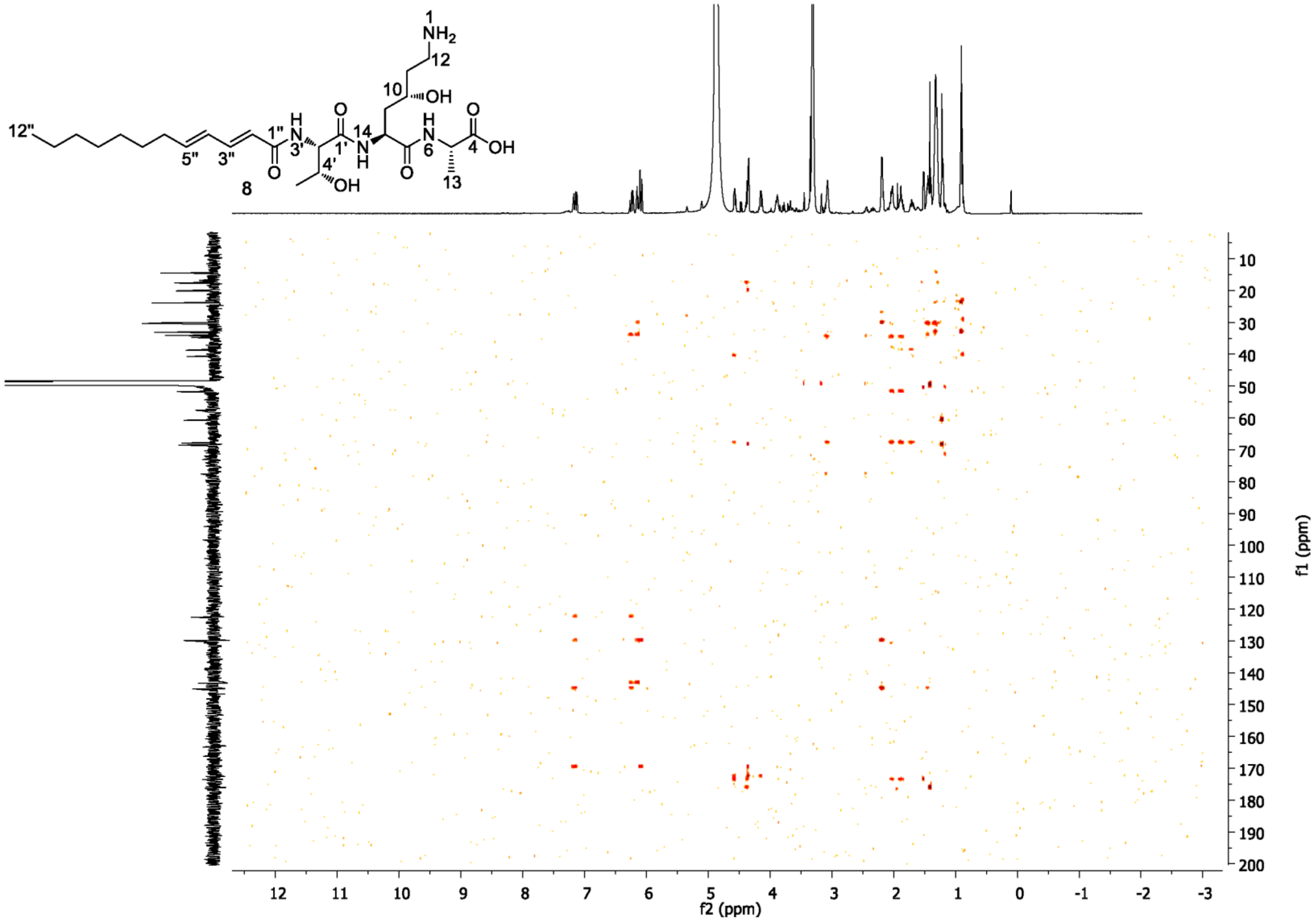

Figure S47. HMBC (methanol-d4) spectrum of **8**.

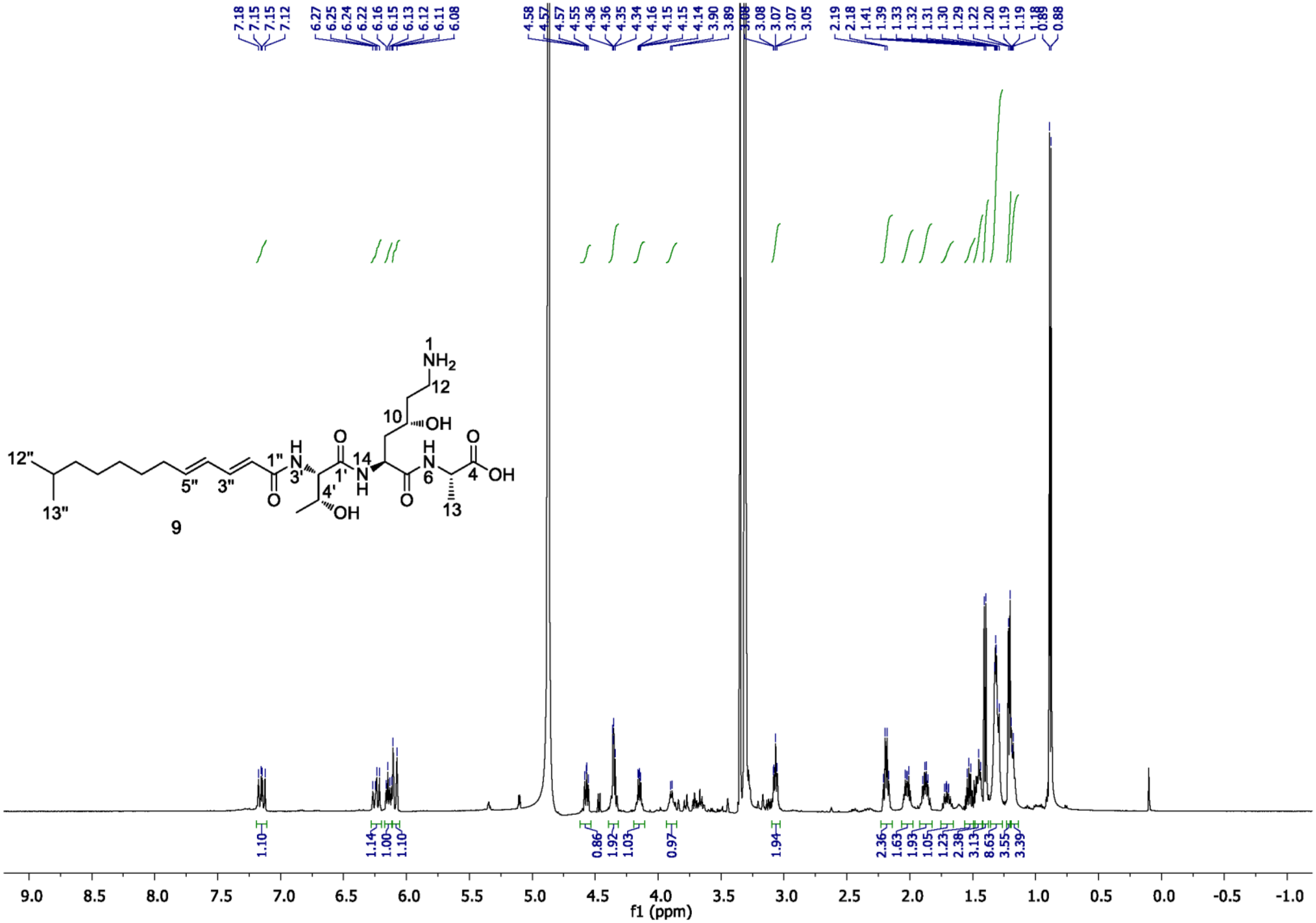

Figure S48. ^{1}H NMR (500 MHz, methanol-d$_4$) spectrum of **9**.

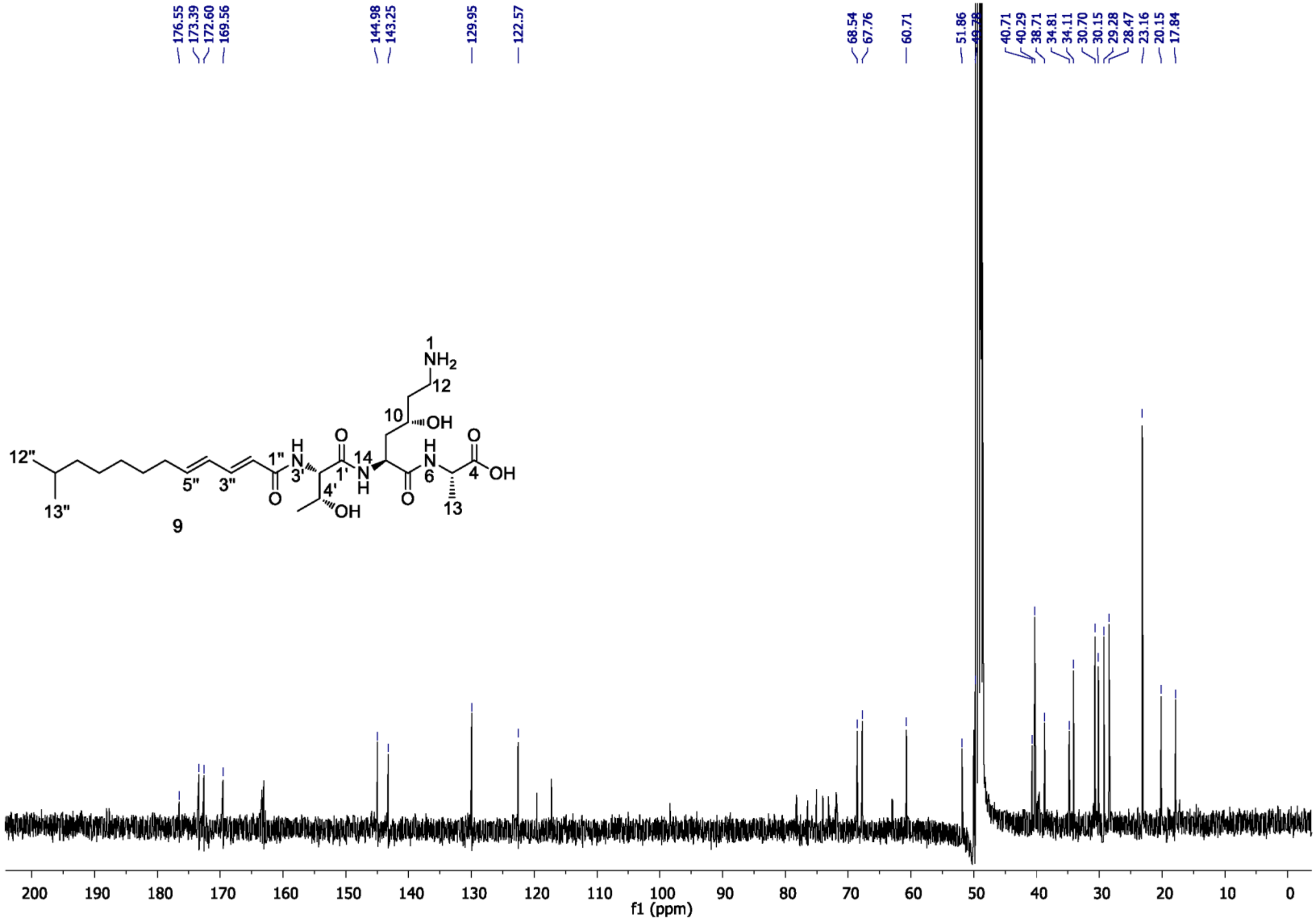

Figure S49. ^{13}C NMR (125 MHz, methanol-d$_4$) spectrum of **9**.

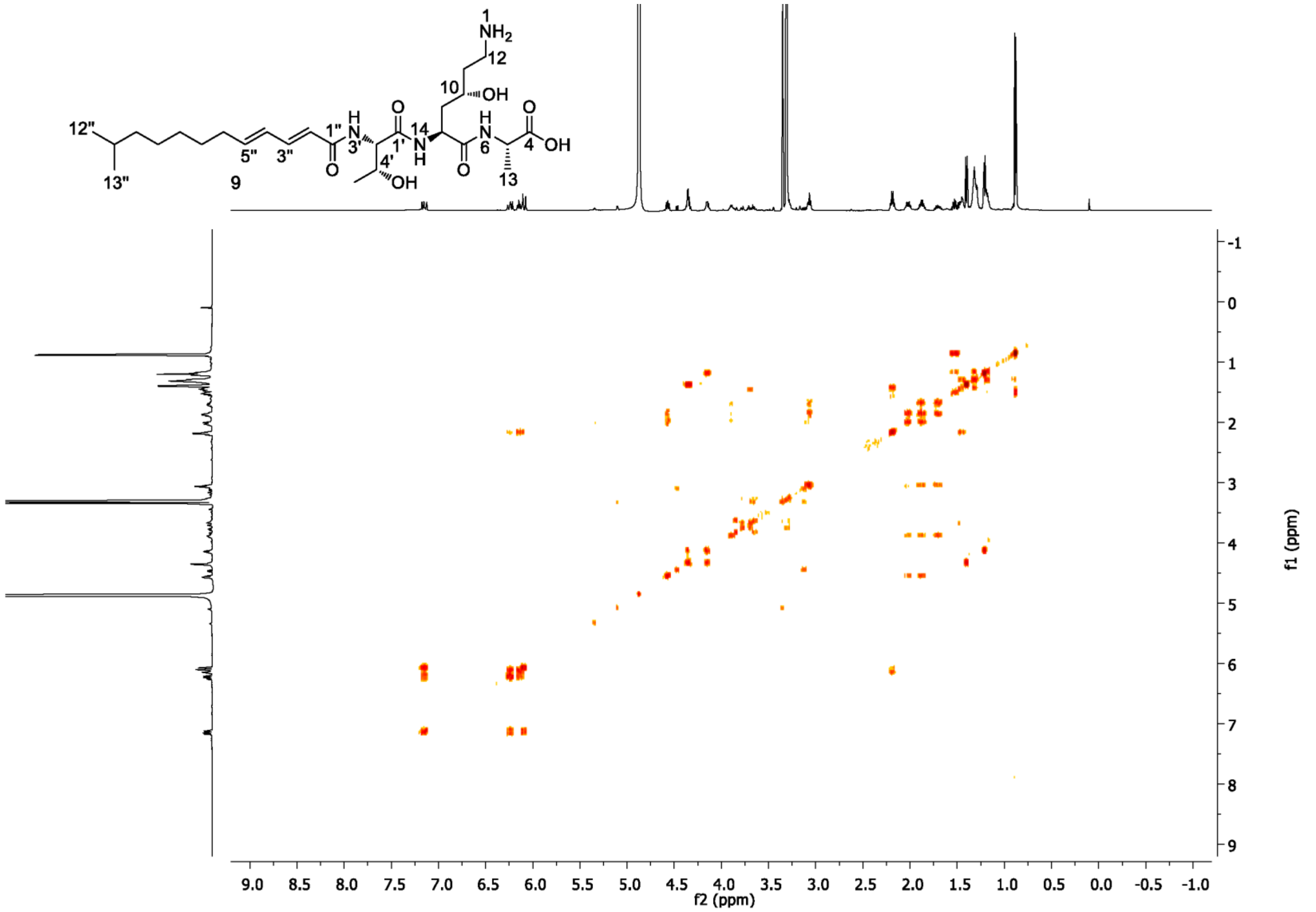

Figure S50. COSY (methanol-d4) spectrum of **9**.

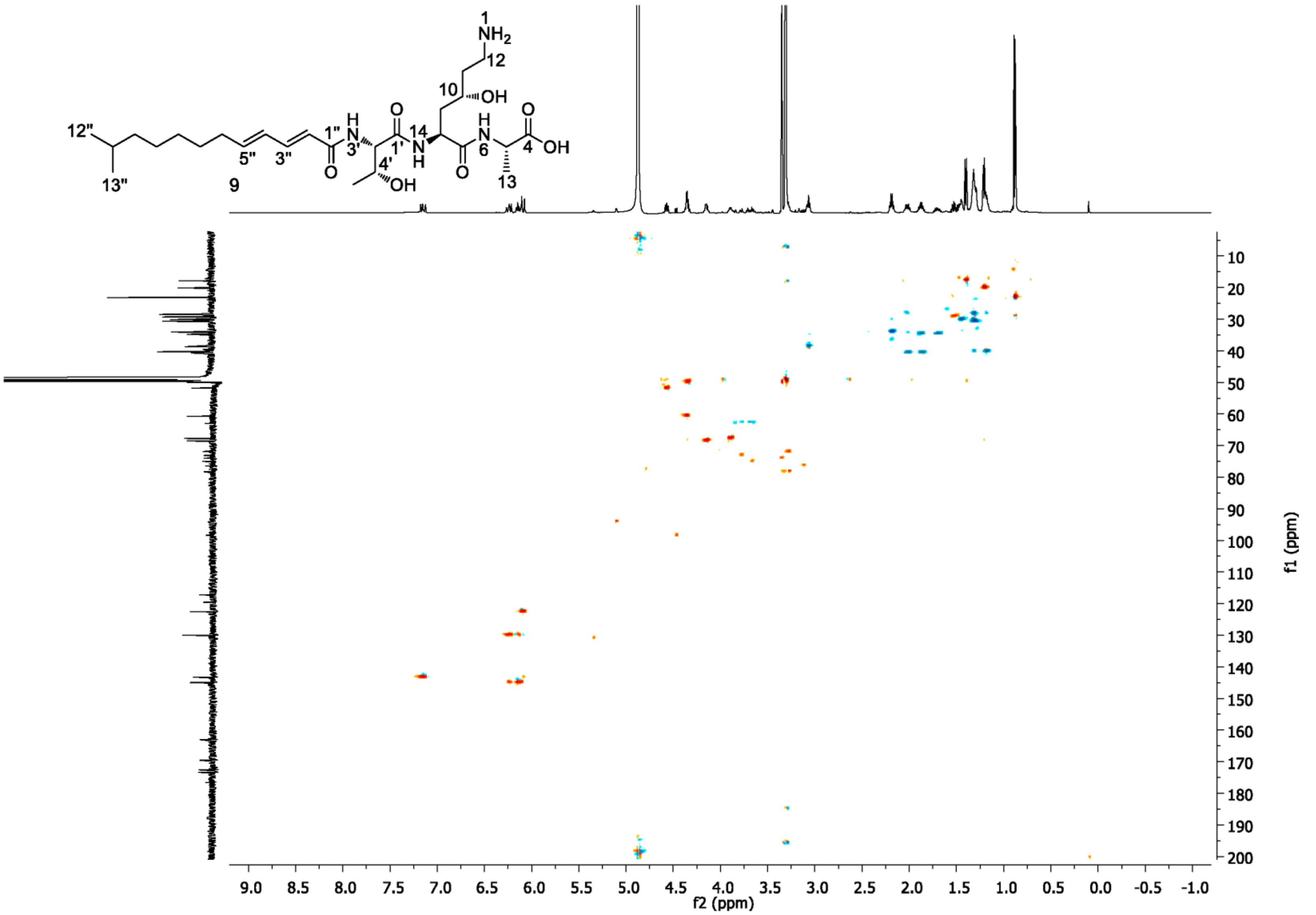

Figure S51. HSQC (methanol-d$_4$) spectrum of **9**.

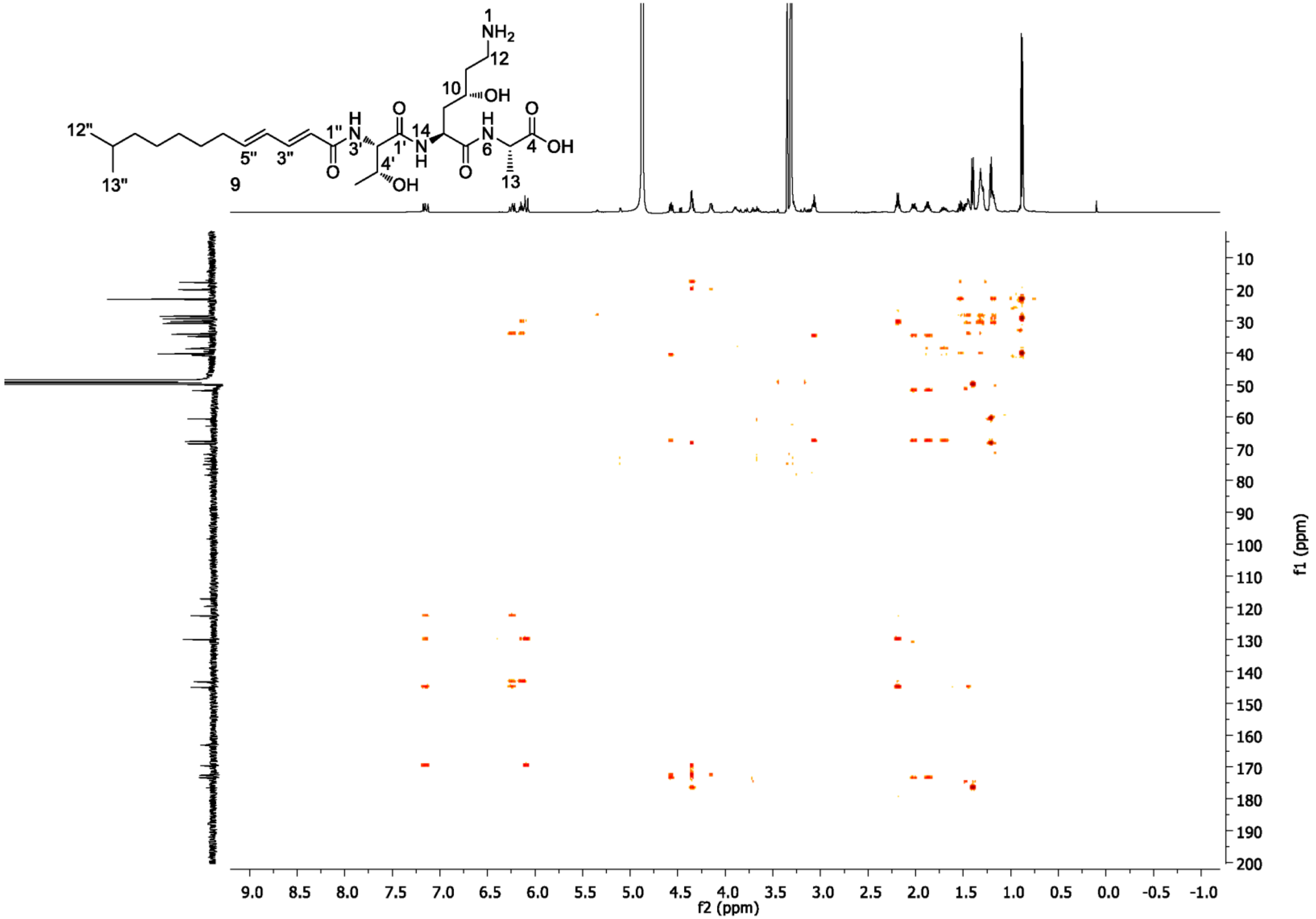

Figure S52. HMBC (methanol-d4) spectrum of **9**.

References

[1] E. Bode, A. O. Brachmann, C. Kegler, R. Simsek, C. Dauth, Q. Zhou, M. Kaiser, P. Klemmt, H. B. Bode, *ChemBioChem* **2015**, *16*, 1115–1119.

[2] L. Zhao, R. M. Awori, M. Kaiser, J. Groß, T. Opatz, H. B. Bode, *J. Nat. Prod.* **2019**, *82*, 3499–3503.

[3] N. J. Tobias, H. Wolff, B. Djahanschiri, F. Grundmann, M. Kronenwerth, Y.-M. Shi, S. Simonyi, P. Grün, D. Shapiro-Ilan, S. J. Pidot, *Nat. Microbiol.* **2017**, *2*, 1676–1685.

[4] M. Wang, J. J. Carver, V. V Phelan, L. M. Sanchez, N. Garg, Y. Peng, D. D. Nguyen, J. Watrous, C. A. Kapono, T. Luzzatto-Knaan, *Nat. Biotechnol.* **2016**, *34*, 828–837.

[5] X. Cai, V. L. Challinor, L. Zhao, D. Reimer, H. Adihou, P. Grün, M. Kaiser, H. B. Bode, *Org. Lett.* **2017**, *19*, 806–809.

[6] N. Gallastegui, M. Groll, *Methods Mol. Biol.* **2012**, *832*, 373–390.

[7] W. Kabsch, *Acta Crystallogr. Sect. D Biol. Crystallogr.* **2010**, *66*, 133–144.

[8] E. M. Huber, W. Heinemeyer, X. Li, C. S. Arendt, M. Hochstrasser, M. Groll, *Nat. Commun.* **2016**, *7*, 1–10.

[9] E. Potterton, P. Briggs, M. Turkenburg, E. Dodson, *Acta Crystallogr. Sect. D Biol. Crystallogr.* **2003**, *59*, 1131–1137.

[10] D. Turk, *Acta Crystallogr. Sect. D Biol. Crystallogr.* **2013**, *69*, 1342–1357.

[11] P. Emsley, B. Lohkamp, W. G. Scott, K. Cowtan, *Acta Crystallogr. Sect. D Biol. Crystallogr.* **2010**, *66*, 486–501.

[12] J. Lowe, D. Stock, B. Jap, P. Zwickl, W. Baumeister, R. Huber, *Science* **1995**, *268*, 533–539.

[13] I. Orhan, B. Şener, M. Kaiser, R. Brun, D. Tasdemir, *Mar. Drugs* **2010**, *8*, 47–58.

[14] M. Oka, K. Yaginuma, K. Numata, M. Konishi, T. Oki, H. Kawaguchi, *J. Antibiot.* **1988**, *41*, 1338–1350.

[15] M. L. Stein, P. Beck, M. Kaiser, R. Dudler, C. F. W. Becker, M. Groll, *Proc. Natl. Acad. Sci.* **2012**, *109*, 18367–18371.

[16] W. Kabsch, *Acta Crystallogr. Sect. D Biol. Crystallogr.* **2010**, *66*, 125–132.

[17] O. Schimming, F. Fleischhacker, F. I. Nollmann, H. B. Bode, *Chembiochem* **2014**, *15*, 1290–1294.

[18] S. R, P. U, Puhler A, *Nat. Biotechnol.* **1983**, *1*, 784–790.

[19] S. Thoma, M. Schobert, *FEMS Microbiol. Lett.* **2009**, *294*, 127–132.

[20] E. Duchaud, C. Rusniok, L. Frangeul, C. Buchrieser, A. Givaudan, S. Taourit, S. Bocs, C. Boursaux-Eude, M. Chandler, J.-F. Charles, *Nat. Biotechnol.* **2003**, *21*, 1307–1313.

[21] M. Fischer-Le Saux, V. Viallard, B. Brunel, P. Normand, N. E. Boemare, *Int. J. Syst. Evol. Microbiol.* **1999**, *49*, 1645–1656.

[22] K. A. J. Bozhüyük, F. Fleischhacker, A. Linck, F. Wesche, A. Tietze, C.-P. Niesert, H. B. Bode, *Nat. Chem.* **2018**, *10*, 275–281.

[23] S. A. Joyce, A. O. Brachmann, I. Glazer, L. Lango, G. Schwär, D. J. Clarke, H. B. Bode, *Angew. Chemie Int. Ed.* **2008**, *47*, 1942–1945.

[24] S. W. Fuchs, K. A. J. Bozhüyük, D. Kresovic, F. Grundmann, V. Dill, A. O. Brachmann, N. R. Waterfield, H. B. Bode, *Angew. Chemie Int. Ed.* **2013**, *52*, 4108–4112.
